# Bose–Einstein Condensation Beyond the Gross–Pitaevskii Regime

**DOI:** 10.1007/s00023-020-01004-1

**Published:** 2020-12-26

**Authors:** Arka Adhikari, Christian Brennecke, Benjamin Schlein

**Affiliations:** 1 Department of Mathematics, Harvard University, One Oxford Street, Cambridge, MA 02138 USA; 2 Institute of Mathematics, University of Zurich, Winterthurerstrasse 190, 8057 Zurich, Switzerland

## Abstract

We consider N bosons in a box with volume one, interacting through a two-body potential with scattering length of the order $N^{-1+\kappa }$, for $\kappa >0$. Assuming that $\kappa \in (0;1/43)$, we show that low-energy states exhibit Bose–Einstein condensation and we provide bounds on the expectation and on higher moments of the number of excitations.

## Introduction

We consider systems of $N\in {\mathbb {N}}$ bosons trapped in the box $\Lambda = [0;1]^3$ with periodic boundary conditions (the three-dimensional torus with volume one) and interacting through a repulsive potential with scattering length of the order $N^{-1+\kappa }$, for $\kappa \in (0; 1/43)$. We are interested in the limit of large *N*. The Hamilton operator has the form

1.1 $$\begin{aligned} H_N = \sum _{i=1}^N -\Delta _{x_i} + \sum _{1\le i<j\le N} N^{2-2\kappa } V (N^{1-\kappa }(x_i -x_j)) \end{aligned}$$

and acts on a dense subspace of $L^2_s (\Lambda ^N)$, the Hilbert space consisting of functions in $L^2 (\Lambda ^N)$ that are invariant with respect to permutations of the $N\in {\mathbb {N}}$ particles. Here, we assume the interaction potential $V \in L^3 ({\mathbb {R}}^3)$ to have compact support and to be nonnegative, ie. $V(x) \ge 0$ for almost all $x \in {\mathbb {R}}^3$.

For $\kappa = 0$, the Hamilton operator (1.1) describes bosons in the so-called Gross–Pitaevskii limit. This regime is frequently used to model trapped Bose gases observed in recent experiments. Another important regime is the thermodynamic limit, where *N* bosons interacting through a fixed potential *V* (independent of *N*) are trapped in the box $\Lambda _L = [0;L]^3$ and where the limits $N,L \rightarrow \infty$ are taken, keeping the density $\rho = N/ L^3$ fixed. After rescaling lengths (introducing new coordinates $x' = x / L$), the Hamilton operator of the Bose gas in the thermodynamic limit is given (up to a multiplicative constant) by (1.1), with $\kappa = 2/3$. Choosing $0< \kappa < 2/3$, we are interpolating therefore between the Gross–Pitaevskii and the thermodynamic limits.

The goal of this paper is to show that low-energy states of (1.1) exhibit Bose–Einstein condensation in the zero-momentum mode $\varphi _0 \in L^2 (\Lambda )$ defined by $\varphi _0 (x) = 1$ for all $x \in \Lambda$ and to give bounds on the number of excitations of the condensate. To achieve this goal, it is convenient to switch to an equivalent representation of the bosonic system, removing the condensate and focusing instead on its orthogonal excitations. To this end, we notice that every $\psi _N \in L^2_s (\Lambda ^N)$ can be uniquely decomposed as

$$\begin{aligned} \psi _N = \alpha _0 \varphi _0^{\otimes N} + \alpha _1 \otimes _s \varphi _0^{\otimes (N-1)} + \alpha _2 \otimes _s \varphi _0^{\otimes (N-2)} + \cdots + \alpha _N \end{aligned}$$

where $\otimes _s$ denotes the symmetric tensor product and $\alpha _j \in L^2_\perp (\Lambda )^{\otimes _s j}$ for all $j = 0, \ldots , N$, with $L^2_\perp (\Lambda )$ the orthogonal complement in $L^2 (\Lambda )$ of $\varphi _0$. This observation allows us to define a unitary map $U_N : L^2_s (\Lambda ^N) \rightarrow {\mathcal {F}}_+^{\le N} = \bigoplus _{j=0}^N L^2_\perp (\Lambda )^{\otimes _s j}$ by setting

1.2 $$\begin{aligned} U_N \psi _N = \{ \alpha _0, \alpha _1, \ldots , \alpha _N \}. \end{aligned}$$

The truncated Fock space ${\mathcal {F}}_+^{\le N} = \bigoplus _{j=0}^N L^2_\perp (\Lambda )^{\otimes _s j}$ is used to describe orthogonal excitations of the condensate (some properties of the map $U_N$ will be discussed in Sect. 2 below). On ${\mathcal {F}}_+^{\le N}$, we introduce the number of particles operator, defining $({\mathcal {N}}_+ \xi )^{(n)} = n \xi ^{(n)}$ for every $\xi = \{ \xi ^{(0)} , \ldots \xi ^{(N)} \} \in {\mathcal {F}}_+^{\le N}$.

We are now ready to state our main theorem, which provides estimates of the expectation and on higher moments of the number of orthogonal excitations of the Bose–Einstein condensate for low-energy states of (1.1).

### Theorem 1.1

Let $V\in L^3({\mathbb {R}}^3)$ be pointwise nonnegative and spherically symmetric. Let $\mathfrak {a}_0 > 0$ denote the scattering length of *V*. Let $H_N$ be defined as in (1.1) with $0< \kappa < 1/43$. Then, for every $\varepsilon > 0$, there exists a constant $C>0$ such that

1.3 $$\begin{aligned} \big | E_N -4\pi {\mathfrak {a}}_0 N^{1+\kappa }\big | \le C N^{43\kappa + \varepsilon } . \end{aligned}$$

for all $N \in {\mathbb {N}}$ large enough.

Let $\psi _N \in L^2_s (\Lambda ^N)$ with $\Vert \psi _N \Vert = 1$ and

1.4 $$\begin{aligned} \langle \psi _N, (H_N - E_N)^2 \psi _N \rangle \le \zeta ^2, \end{aligned}$$

for a $\zeta > 0$. Then, for every $\varepsilon > 0$ there exists a constant $C > 0$ such that

1.5 $$\begin{aligned} \langle U_N \psi _N , {\mathcal {N}}_+ \, U_N \psi _N \rangle \le C \left[ \zeta + \zeta ^2 N^{13\kappa + \varepsilon - 1} + N^{43 \kappa + 4\varepsilon } \right] \end{aligned}$$

for all $N \in {\mathbb {N}}$ large enough. If moreover $\psi _N = \chi (H_N \le E_N + \zeta ) \psi _N$, then for all $k \in {\mathbb {N}}$ and all $\varepsilon > 0$ there exists $C > 0$ such that

1.6 $$\begin{aligned} \langle U_N \psi _N , {\mathcal {N}}_+^k \, U_N \psi _N \rangle \le C \left[ N^{20\kappa + \varepsilon } \zeta ^2 + N^{44 \kappa + 2 \varepsilon } \right] ^{k} \end{aligned}$$

for all $N \in {\mathbb {N}}$ large enough.

The convergence $E_N/4\pi \mathfrak {a}_0 N^{1+\kappa } \rightarrow 1$, as $N \rightarrow \infty$, has been first established, for Bose gases trapped by an external potential, in [19] (the choice $\kappa > 0$ corresponds, in the terminology of [19], to the Thomas–Fermi limit).

It follows from (1.5) that the one-particle density matrix $\gamma _N = \mathrm{tr}_{2,\ldots , N} |\psi _N \rangle \langle \psi _N |$ associated with a normalized $\psi _N \in L^2_s (\Lambda ^N)$ satisfying (1.4) is such that

1.7 $$\begin{aligned} \begin{aligned} 1- \langle \varphi _0 , \gamma _N \varphi _0 \rangle = \;&\frac{1}{N} \left[ N - \langle \psi _N, a^* (\varphi _0) a(\varphi _0) \psi _N \rangle \right] \\ = \;&\frac{1}{N} \langle U_N \psi _N , {\mathcal {N}}_+ \, U_N \psi _N \rangle \\ \le \;&C \left[ \zeta N^{-1} + \zeta ^2 N^{13\kappa + \varepsilon - 2} + N^{43 \kappa + 4\varepsilon -1} \right] \end{aligned} \end{aligned}$$

as $N \rightarrow \infty$. Here, we used the formula $U_N a^* (\varphi _0) a(\varphi _0) U_N = N - {\mathcal {N}}_+$; see (2.5). Equation (1.7) implies that low-energy states of (1.1) exhibit complete Bose–Einstein condensation, **if** $\kappa < 1/43$.

We remark that the estimate (1.6) follows, in our analysis, from a stronger bound controlling not only the number but also the energy of the excitations of the condensate. As we will explain in Sect. 3, in order to estimate the energy of excitations in low-energy states, we first need to remove (at least part of) their correlations. If we choose, as we do in (1.6), $\psi _N \in L^2_s (\Lambda ^N)$ with $\Vert \psi _N \Vert = 1$ and $\psi _N = \chi (H_N \le E_N + \zeta ) \psi _N$, we can introduce the corresponding renormalized excitation vector $\xi _N = e^B U_N \psi _N \in {\mathcal {F}}_+^{\le N}$, with the antisymmetric operator *B* defined as in (3.21) (the unitary operator $e^B$ will be referred to as a generalized Bogoliubov transformation). We will show in Sect. 6 that for every $k \in {\mathbb {N}}$, there exists $C > 0$ such that

1.8 $$\begin{aligned} \langle \xi _N , ({\mathcal {H}}_N + 1) ( {\mathcal {N}}_+ + 1)^{2k} \xi _N \rangle \le C \left[ N^{20\kappa + \varepsilon } \zeta ^2 + N^{44 \kappa + 2 \varepsilon } \right] ^{2k+1} \end{aligned}$$

for all *N* large enough. Here ${\mathcal {H}}_N = {\mathcal {K}}+ {\mathcal {V}}_N$, where

1.9 $$\begin{aligned} {\mathcal {K}}= \sum _{p \in \Lambda _+^*} p^2 a_p^* a_p , \quad \text {and} \quad {\mathcal {V}}_N = \frac{1}{2N} \sum _{\begin{array}{c} p,q \in \Lambda _+^*, r \in \Lambda ^* : \\ r \not = -p, -q \end{array}} N^{\kappa } {\widehat{V}} (r/N^{1-\kappa }) a_{p+r}^* a_q^* a_{q+r} a_p \end{aligned}$$

are the kinetic and potential energy operators, restricted to ${\mathcal {F}}_+^{\le N}$. (Here, ${\widehat{V}}$ is the Fourier transform of the potential *V*, defined as in (2.4).) Equation (1.6) follows then from (1.8), because ${\mathcal {N}}_+$ commutes with ${\mathcal {H}}_N$, ${\mathcal {N}}_+ \le {\mathcal {K}}\le {\mathcal {H}}_N$ and because conjugation with the generalized Bogoliubov transformation $e^B$ does not change the number of particles substantially; see Lemma 3.2 (for $k \in {\mathbb {N}}$ even, we also use simple interpolation).

In the Gross–Pitaevskii regime corresponding to $\kappa = 0$ the convergence $\gamma _N \rightarrow |\varphi _0 \rangle \langle \varphi _0|$ has been first established in [16–18] and later, using a different approach, in [21].^1^ In this case (ie. $\kappa = 0$), the bounds (1.3), (1.5) and (1.6) with $\varepsilon = 0$ (which are optimal in their *N*-dependence) have been shown in [4]. Previously, they have been established in [2], under the additional assumption of small potential. A simpler proof of the results of [2], extended also to systems of bosons trapped by an external potential, has been recently given in [20]. The result of [4] was used in [3] to determine the second order corrections to the ground state energy and the low-energy excitation spectrum of the Bose gas in the Gross–Pitaevskii regime. Note that our approach in the present paper could be easily extended to the case $\kappa = 0$, leading to the same bounds obtained in [4]. We exclude the case $\kappa = 0$ because we would have to modify certain definitions, making the notation more complicated (for example, the sets $P_H$ in (3.14) and $P_L$ in (4.2) would have to be defined in terms of cutoffs independent of *N*).

The methods of [16–18] can also be extended to show Bose–Einstein condensation for low-energy states of (1.1), for some $\kappa > 0$. In fact, following the proof of [18, Theorem 5.1], it is possible to show that for a normalized $\psi _N \in L^2_s (\Lambda ^N)$ with $\Vert \psi _N \Vert =1$ and such that $\langle \psi _N, H_N \psi _N \rangle \le E_N + \zeta$, the expectation of the number of excitations is bounded by

1.10 $$\begin{aligned} \langle U_N \psi _N , {\mathcal {N}}_+ U_N \psi _N \rangle \le C \left[ N^{\frac{15 +20\kappa }{17}} + \zeta \right] \end{aligned}$$

which implies complete Bose–Einstein condensation for low-energy states, for all $\kappa < 1/10$. For sufficiently small $\kappa > 0$, Theorem 1.1 improves (1.10) because it gives a better rate^2^ (if $\kappa < 15/711$) and because, through (1.6), it also provides (under stronger conditions on $\psi _N$) bounds for higher moments of the number of excitations ${\mathcal {N}}_+$.

In [10], in a slightly different setting, the authors obtain a bound of the form (1.6) for $k=1$, for the choice $\kappa = 1/(55+1/3)$ (for normalized $\psi _N \in L^2_s (\Lambda ^N)$ that satisfy $\langle \psi _N, H_N\psi _N\rangle \le E_N + \zeta$). They use this result to show a lower bound on the ground state energy of the dilute Bose gas in the thermodynamic limit matching the prediction of Lee–Yang and Lee–Huang–Yang [13, 14].

After completion of our work, two more papers have appeared whose results are related with Theorem 1.1. Based on localization arguments from [8, 10], a bound for the expectation of ${\mathcal {N}}_+$ in low-energy states has been shown in [9], establishing Bose–Einstein condensation for all $\kappa < 2/5$ (as pointed out there, using a refined analysis similar to that of [10], the range of $\kappa$ can be slightly improved). On the other hand, following an approach similar to [2], but with substantial simplifications (partly due to the fact that the author works in the grand canonical, rather than the canonical, ensemble), a new proof of Bose–Einstein condensation was obtained in [11], in the Gross–Pitaevskii regime, under the assumption of small potential. There is hope that the approach of [11] can be extended beyond the Gross–Pitaevskii regime, providing a simplified proof of Theorem 1.1, potentially allowing for larger values of $\kappa$.

The derivation of the bounds (1.5), (1.6), (1.8) is crucial to resolve the low-energy spectrum of the Hamiltonian (1.1). The extension of estimates on the ground state energy and on the excitation spectrum obtained in [3] for the Gross–Pitaevskii limit, to regimes with $\kappa > 0$ small enough will be addressed in a separate paper [6], using the results of Theorem 1.1. With our techniques, it does not seem possible to obtain such precise information on the spectrum of (1.1) using only previously available bounds like (1.10).

Let us now briefly explain the strategy we use to prove Theorem 1.1. The first part of our analysis follows closely [4]. We start in Sect. 2 by introducing the excitation Hamiltonian ${\mathcal {L}}_N = U_N H_N U_N^*$, acting on the truncated Fock space ${\mathcal {F}}_+^{\le N}$; the result is given in (2.6), (2.7). The vacuum expectation $\langle \Omega , {\mathcal {L}}_N \Omega \rangle = N^{1+\kappa } {\widehat{V}} (0)/2$ is still very far from the correct ground state energy of ${\mathcal {L}}_N$ (and thus of $H_N$); the difference is of order $N^{1+\kappa }$. This is a consequence of the definition (1.2) of the unitary map $U_N$, whose action removes products of the condensate wave function $\varphi _0$, leaving however all correlations among particles in the wave functions $\alpha _j \in L^2_\perp (\Lambda )^{\otimes _s j}$, $j=1, \ldots , N$.

To factor out correlations, we introduce in Sect. 3 a renormalized excitation Hamiltonian ${\mathcal {G}}_N = e^{-B} {\mathcal {L}}_N e^B$, defined through unitary conjugation of ${\mathcal {L}}_N$ with a generalized Bogoliubov transformation $e^B$. The antisymmetric operator $B: {\mathcal {F}}_+^{\le N} \rightarrow {\mathcal {F}}_+^{\le N}$ is quadratic in the modified creation and annihilation operators $b_p, b_p^*$ defined, for every momentum $p \in \Lambda ^*_+ = 2\pi {\mathbb {Z}}^3 \backslash \{0 \}$, in (2.8) ($b^*_p$ creates a particle with momentum *p* annihilating, at the same time, a particle with momentum zero; in other words, $b_p^*$ creates an excitation, moving a particle out of the condensate). The properties of ${\mathcal {G}}_N$ are listed in Prop. 3.3. In particular, Proposition 3.3 implies that to leading order, $\langle \Omega , {\mathcal {G}}_N \Omega \rangle \simeq 4\pi \mathfrak {a}_0 N^{1+\kappa }$, if $\kappa$ is small enough.

Unfortunately, ${\mathcal {G}}_N$ is not coercive enough to prove directly that low-energy states exhibit condensation (in the sense that it is not clear how to estimate the difference between ${\mathcal {G}}_N$ and its vacuum expectation from below by the number of particle operator ${\mathcal {N}}_+$). For this reason, in Sect. 4, we define yet another renormalized excitation Hamiltonian ${\mathcal {J}}_N = e^{-A} {\mathcal {G}}_N e^A$, where now *A* is the antisymmetric operator (4.1), cubic in (modified) creation and annihilation operators (to be more precise, we only conjugate the main part of ${\mathcal {G}}_N$ with $e^A$; see (4.3)). Important properties of ${\mathcal {J}}_N$ are stated in Proposition 4.1. Up to negligible errors, the conjugation with $e^A$ completes the renormalization of quadratic and cubic terms; in (4.5), these terms have the same form they would have for particles interacting through a mean-field potential with Fourier transform $8\pi \mathfrak {a}_0 N^\kappa {\mathbf{1}} (|p| < N^\alpha )$, with a parameter $\alpha > 0$ that will be chosen small enough, depending on $\kappa$ (in other words, the renormalization procedure allows us to replace, in all quadratic and cubic terms, the original interaction with Fourier transform $N^{-1+\kappa } {\widehat{V}} (p/ N^{1-\kappa })$ decaying only for momenta $|p| > N^{1-\kappa }$, with a potential whose Fourier transform already decays on scales $N^{\alpha } \ll N^{1-\kappa }$).

The main problem with ${\mathcal {J}}_N$ is that its quartic terms (the restriction of the initial potential energy on the orthogonal complement of the condensate wave function) are still proportional to the local interaction with Fourier transform $N^{-1+\kappa } {\widehat{V}} (p / N^{1-\kappa })$.

One possibility to solve this problem is to neglect the original quartic terms (they are positive) and insert instead quartic terms proportional to the renormalized mean-field potential $8\pi \mathfrak {a}_0 N^\kappa \mathbf{1} (|p| < N^\alpha )$, so that Bose–Einstein condensation follows as it does for mean-field systems (see [22]). Since (with the notation ${\check{\chi }}$ for the inverse Fourier transform of the characteristic function on the ball of radius one)

$$\begin{aligned} \begin{aligned} \frac{8\pi \mathfrak {a}_0 N^\kappa }{N} \sum _{|r| < N^\alpha } a_{p+r}^* a_q^* a_{q+r} a_p&= 8\pi \mathfrak {a}_0 N^{3\alpha + \kappa -1} \int {\check{\chi }} (N^\alpha (x-y)) \check{a}_x^* \check{a}_y^* \check{a}_y \check{a}_x \, dx dy \\&\le C N^{3\alpha + \kappa - 1} {\mathcal {N}}_+^2 \end{aligned} \end{aligned}$$

and since we know from (1.10) that ${\mathcal {N}}_+ \lesssim N^{\frac{15 + 20 \kappa }{17}}$ in low-energy states, the insertion of the renormalized quartic terms produces an error that can be controlled by localization in the number of particles, if

$$\begin{aligned} 3 \alpha + \kappa - 1 + \frac{15 + 20 \kappa }{17} = 3 \alpha + \frac{37}{17} \kappa - \frac{2}{17} < 0 \end{aligned}$$

This strategy was used in [4] to prove Bose–Einstein condensation with optimal rate in the Gross–Pitaevskii regime $\kappa = 0$ (in this case, one can choose $\alpha = 0$).

Here, we follow a different approach. We perform a last renormalization step, conjugating ${\mathcal {J}}_N$ through a unitary operator $e^D$, with *D* quartic in creation and annihilation operators. This leads to a new Hamiltonian ${\mathcal {M}}_N= e^{-D} {\mathcal {J}}_N e^D$ (in fact, it is more convenient to conjugate only the main part of ${\mathcal {J}}_N$, ignoring small contributions that can be controlled by other means; see (5.5)), where the original interaction $N^{-1+\kappa } {\widehat{V}} (p/ N^{1-\kappa })$ is replaced by the mean-field potential $8\pi \mathfrak {a}_0 N^\kappa \mathbf{1 } (|p| < N^\alpha )$ in all relevant terms.^3^ Condensation can then be shown as it is done for mean-field systems, with no need for localization. This is the main novelty of our analysis, compared with [4]. In Sect. 5, we define the final Hamiltonian ${\mathcal {M}}_N$ and in Proposition 5.1 we bound it from below. The proof of Proposition 5.1, which is technically the main part of our paper, is deferred to Sect. 7. In Sect. 6, we combine the results of the previous sections to conclude the proof of Theorem 1.1.

The results we prove with our new technique are stronger than what we would obtain using the approach of [4] in the sense that they allow for larger values of $\kappa$ and better rates. More importantly, we believe that the approach we propose here is more natural and that it leaves more space for extensions. In particular, with the final quartic renormalization step, we map the original Hamilton operator (1.1), with an interaction varying on momenta of order $N^{1-\kappa }$, into a new Hamiltonian having the same form, but now with an interaction restricted to momenta smaller than $N^{\alpha }$. If $\alpha < 1-\kappa$, this leads to an effective regularization of the potential and it suggests that further improvements may be achieved by iteration; we plan to follow this strategy, which bears some similarities to the renormalization group analysis developed in [1], in future work.

In order to control errors arising from the quartic conjugation, it is important to use observables that were not employed in [4]. In particular, the expectation of the number of excitations with large momenta

$$\begin{aligned} {\mathcal {N}}_{\ge N^\gamma } = \sum _{p\in \Lambda ^*_+ : |p| \ge N^\gamma } a_p^* a_p \end{aligned}$$

and of its powers ${\mathcal {N}}^2_{\ge N^\gamma }, {\mathcal {N}}^3_{\ge N^\gamma }$, as well as the expectation of products of the form ${\mathcal {K}}_L {\mathcal {N}}_{\ge N^\gamma }$ and ${\mathcal {K}}_L {\mathcal {N}}_{\ge N^\gamma }^2$, involving the kinetic energy operator restricted to low momenta ${\mathcal {K}}_L$, will play a crucial role in our analysis. It will therefore be important to establish bounds for the growth of these observables through all steps of the renormalization procedure (Lemmas 4.2, 4.3, 7.1, 7.2). In Sect. 6, an important step in the proof of Theorem 1.1 will consist in controlling the expectation of these observables on low-energy states of the renormalized Hamiltonian ${\mathcal {G}}_N$.

## The Excitation Hamiltonian

We denote by ${\mathcal {F}}= \bigoplus _{n \ge 0} L^2 (\Lambda )^{\otimes _s n}$ the bosonic Fock space over the one-particle space $L^2 (\Lambda )$ and by $\Omega = \{ 1, 0, \ldots \}$ the vacuum vector. We can define the number of particles operator ${\mathcal {N}}$ by setting $({\mathcal {N}}\psi )^{(n)} = n \psi ^{(n)}$ for all $\psi = \{ \psi ^{(0)}, \psi ^{(1)}, \ldots \}$ in a dense subspace of ${\mathcal {F}}$. For every one-particle wave function $g \in L^2 (\Lambda )$, we define the creation operator $a^* (g)$ and its hermitian conjugate, the annihilation operator *a*(*g*), through

$$\begin{aligned} \begin{aligned} (a^* (g) \Psi )^{(n)} (x_1, \ldots , x_n)&= \frac{1}{\sqrt{n}} \sum _{j=1}^n g (x_j) \Psi ^{(n-1)} (x_1, \ldots , x_{j-1}, x_{j+1} , \ldots , x_n) \\ (a (g) \Psi )^{(n)} (x_1, \ldots , x_n)&= \sqrt{n+1} \int _\Lambda {\bar{g}} (x) \Psi ^{(n+1)} (x,x_1, \ldots , x_n) \, dx \end{aligned} \end{aligned}$$

Creation and annihilation operators are defined on the domain of ${\mathcal {N}}^{1/2}$, where they satisfy the bounds

$$\begin{aligned} \Vert a (f) \psi \Vert \le \Vert f \Vert \Vert {\mathcal {N}}^{1/2} \psi \Vert , \qquad \Vert a^* (f) \psi \Vert \le \Vert f \Vert \Vert ({\mathcal {N}}_+ + 1)^{1/2} \psi \Vert \end{aligned}$$

and the canonical commutation relations

2.1 $$\begin{aligned} {[} a (g), a^* (h) ] = \langle g,h \rangle , \quad [ a(g), a(h)] = [a^* (g), a^* (h) ] = 0 \end{aligned}$$

for all $g,h \in L^2 (\Lambda )$ ($\langle . , . \rangle$ denotes here the inner product on $L^2 (\Lambda )$). For $p \in \Lambda ^* = 2\pi {\mathbb {Z}}^3$, we define the plane wave $\varphi _p \in L^2 (\Lambda )$ through $\varphi _p (x) = e^{-i p \cdot x}$ for all $x \in \Lambda$, and the operators $a^*_p = a (\varphi _p)$ and $a_p = a (\varphi _p)$ creating and, respectively, annihilating a particle with momentum *p*. It is sometimes convenient to switch to position space, introducing operator valued distributions $\check{a}_x, \check{a}_x^*$ such that

$$\begin{aligned} a(f) = \int _\Lambda {\bar{f}} (x) \, \check{a}_x \, dx , \quad a^* (f) = \int _\Lambda f(x) \, \check{a}_x^* \, dx \end{aligned}$$

In terms of creation and annihilation operators, the number of particles operator can be written as

$$\begin{aligned} {\mathcal {N}}= \sum _{p \in \Lambda ^*} a_p^* a_p = \int a_x^* a_x \, dx \end{aligned}$$

We will describe excitations of the Bose–Einstein condensate on the truncated Fock space

$$\begin{aligned} {\mathcal {F}}_+^{\le N} = \bigoplus _{j=0}^N L_\perp ^2 (\Lambda )^{\otimes _s j} \end{aligned}$$

constructed over the orthogonal complement $L^2_\perp (\Lambda )$ of the condensate wave function $\varphi _0$. On ${\mathcal {F}}_+^{\le N}$, we denote the number of particles operator by ${\mathcal {N}}_+$. It is given by ${\mathcal {N}}_+ = \sum _{p \in \Lambda ^*_+} a_p^* a_p$, where $\Lambda ^*_+ = \Lambda ^* \backslash \{ 0 \} = 2\pi {\mathbb {Z}}^3 \backslash \{ 0 \}$ is the momentum space for excitations. Given $\Theta \ge 0$, we also introduce the restricted number of particles operators

2.2 $$\begin{aligned} {\mathcal {N}}_{\ge \Theta } = \sum _{p\in \Lambda _+^*: |p| \ge \Theta } a^*_p a_p, \end{aligned}$$

measuring the number of excitations with momentum larger or equal to $\Theta$, and ${\mathcal {N}}_{< \Theta } = {\mathcal {N}}_+ - {\mathcal {N}}_{\ge \Theta }$.

Consider the operator $U_N : L^2_s (\Lambda ^N) \rightarrow {\mathcal {F}}_+^{\le N}$ defined in (1.2). Identifying $\psi _N \in L^2_s (\Lambda ^N)$ with the Fock space vector $\{ 0, \ldots , 0, \psi _N, 0, \ldots \}$, we can also express $U_N$ in terms of creation and annihilation operators; we obtain

$$\begin{aligned} U_N = \bigoplus _{n=0}^N (1-|\varphi _0 \rangle \langle \varphi _0|)^{\otimes n} \frac{a(\varphi _0)^{N-n}}{\sqrt{(N-n)!}} \end{aligned}$$

It is then easy to check that $U_N^* : {\mathcal {F}}_{+}^{\le N} \rightarrow L^2_s (\Lambda ^N)$ is given by

$$\begin{aligned} U_N^* \, \{ \alpha ^{(0)}, \ldots , \alpha ^{(N)} \} = \sum _{n=0}^N \frac{a^* (\varphi _0)^{N-n}}{\sqrt{(N-n)!}} \, \alpha ^{(n)} \end{aligned}$$

and that $U_N^* U_N = 1$, ie. $U_N$ is unitary.

Using $U_N$, we can define the excitation Hamiltonian ${\mathcal {L}}_N := U_N H_N U_N^*$, acting on a dense subspace of ${\mathcal {F}}_+^{\le N}$. To compute ${\mathcal {L}}_N$, we first write the Hamiltonian (1.1) in momentum space, in terms of creation and annihilation operators. We find

2.3 $$\begin{aligned} H_N = \sum _{p \in \Lambda ^*} p^2 a_p^* a_p + \frac{1}{2N^{1-\kappa }} \sum _{p,q,r \in \Lambda ^*} {\widehat{V}} (r/N^{1-\kappa }) a_{p+r}^* a_q^* a_{p} a_{q+r} \end{aligned}$$

where

2.4 $$\begin{aligned} {\widehat{V}} (k) = \int _{{\mathbb {R}}^3} V (x) e^{-i k \cdot x} dx \end{aligned}$$

is the Fourier transform of *V*, defined for all $k \in {\mathbb {R}}^3$ (in fact, (1.1) is the restriction of (2.3) to the $N\in {\mathbb {N}}$-particle sector of the Fock space ${\mathcal {F}}$). We can now determine the excitation Hamiltonian ${\mathcal {L}}_N$ using the following rules, describing the action of the unitary operator $U_N$ on products of a creation and an annihilation operator (products of the form $a_p^* a_q$ can be thought of as operators mapping $L^2_s (\Lambda ^N)$ to itself). For any $p,q \in \Lambda ^*_+ = 2\pi {\mathbb {Z}}^3 \backslash \{ 0 \}$, we find (see [15]):

2.5 $$\begin{aligned} \begin{aligned} U_N \, a^*_0 a_0 \, U_N^*&= N- {\mathcal {N}}_+ \\ U_N \, a^*_p a_0 \, U_N^*&= a^*_p \sqrt{N-{\mathcal {N}}_+ } \\ U_N \, a^*_0 a_p \, U_N^*&= \sqrt{N-{\mathcal {N}}_+ } \, a_p \\ U_N \, a^*_p a_q \, U_N^*&= a^*_p a_q \end{aligned} \end{aligned}$$

We conclude that

2.6 $$\begin{aligned} {\mathcal {L}}_N = {\mathcal {L}}^{(0)}_{N} + {\mathcal {L}}^{(2)}_{N} + {\mathcal {L}}^{(3)}_{N} + {\mathcal {L}}^{(4)}_{N} \end{aligned}$$

with

2.7 $$\begin{aligned} \begin{aligned} {\mathcal {L}}_{N}^{(0)} =\;&\frac{N-1}{2N}N^{\kappa }{\widehat{V}} (0) (N-{\mathcal {N}}_+ ) + \frac{N^{\kappa }{\widehat{V}} (0)}{2N} {\mathcal {N}}_+ (N-{\mathcal {N}}_+ ) \\ {\mathcal {L}}^{(2)}_{N} =\;&\sum _{p \in \Lambda ^*_+} p^2 a_p^* a_p + \sum _{p \in \Lambda _+^*} N^{\kappa }{\widehat{V}} (p/N^{1-\kappa }) \left[ b_p^* b_p - \frac{1}{N} a_p^* a_p \right] \\&+ \frac{1}{2} \sum _{p \in \Lambda ^*_+} N^{\kappa }{\widehat{V}} (p/N^{1-\kappa }) \left[ b_p^* b_{-p}^* + b_p b_{-p} \right] \\ {\mathcal {L}}^{(3)}_{N} =\;&\frac{1}{\sqrt{N}} \sum _{p,q \in \Lambda _+^* : p+q \not = 0} N^{\kappa }{\widehat{V}} (p/N^{1-\kappa }) \left[ b^*_{p+q} a^*_{-p} a_q + a_q^* a_{-p} b_{p+q} \right] \\ {\mathcal {L}}^{(4)}_{N} =\;&\frac{1}{2N} \sum _{\begin{array}{c} p,q \in \Lambda _+^*, r \in \Lambda ^*: \\ r \not = -p,-q \end{array}} N^{\kappa }{\widehat{V}} (r/N^{1-\kappa }) a^*_{p+r} a^*_q a_p a_{q+r} \end{aligned} \end{aligned}$$

where we introduced generalized creation and annihilation operators

2.8 $$\begin{aligned} b^*_p = a^*_p \, \sqrt{\frac{N-{\mathcal {N}}_+}{N}} , \qquad \text {and } \quad b_p = \sqrt{\frac{N-{\mathcal {N}}_+}{N}} \, a_p \end{aligned}$$

for all $p \in \Lambda ^*_+$. Observe that by (2.5),

$$\begin{aligned} U_N^* b_p^* U_N = a^*_p \frac{a_0}{\sqrt{N}}, \qquad U_N^* b_p U_N = \frac{a_0^*}{\sqrt{N}} a_p \end{aligned}$$

In other words, $b_p^*$ creates a particle with momentum $p \in \Lambda ^*_+$ but, at the same time, it annihilates a particle from the condensate; it creates an excitation, preserving the total number of particles in the system. On states exhibiting complete Bose–Einstein condensation in the zero-momentum mode $\varphi _0$, we have $a_0 , a_0^* \simeq \sqrt{N}$ and we can therefore expect that $b_p^* \simeq a_p^*$ and that $b_p \simeq a_p$. Modified creation and annihilation operators satisfy the commutation relations

2.9 $$\begin{aligned} \begin{aligned} {[} b_p, b_q^* ]&= \left( 1 - \frac{{\mathcal {N}}_+}{N} \right) \delta _{p,q} - \frac{1}{N} a_q^* a_p \\ [ b_p, b_q ]&= [b_p^* , b_q^*] = 0 \end{aligned} \end{aligned}$$

Furthermore, we find

2.10 $$\begin{aligned} {[} b_p, a_q^* a_r ] = \delta _{pq} b_r, \qquad [b_p^*, a_q^* a_r] = - \delta _{pr} b_q^* \end{aligned}$$

for all $p,q,r \in \Lambda _+^*$; this implies in particular that $[b_p , {\mathcal {N}}_+] = b_p$, $[b_p^*, {\mathcal {N}}_+] = - b_p^*$. It is also useful to notice that the operators $b^*_p, b_p$, like the standard creation and annihilation operators $a_p^*, a_p$, can be bounded by the square root of the number of particles operators; we find

$$\begin{aligned} \begin{aligned} \Vert b_p \xi \Vert&\le \Big \Vert {\mathcal {N}}_+^{1/2} \Big ( \frac{N+1-{\mathcal {N}}_+}{N} \Big )^{1/2} \xi \Big \Vert \le \Vert {\mathcal {N}}_+^{1/2} \xi \Vert \\ \Vert b^*_p \xi \Vert&\le \Big \Vert ({\mathcal {N}}_+ +1)^{1/2} \Big ( \frac{N-{\mathcal {N}}_+ }{N} \Big )^{1/2} \xi \Big \Vert \le \Vert ({\mathcal {N}}_+ + 1)^{1/2} \xi \Vert \end{aligned} \end{aligned}$$

for all $\xi \in {\mathcal {F}}^{\le N}_+$. Since ${\mathcal {N}}_+ \le N$ on ${\mathcal {F}}_+^{\le N}$, the operators $b_p^* , b_p$ are bounded, with $\Vert b_p \Vert , \Vert b^*_p \Vert \le (N+1)^{1/2}$.

We can also define modified operator valued distributions

$$\begin{aligned} \check{b}_x = \sqrt{\frac{N-{\mathcal {N}}_+}{N}} \, \check{a}_x, \qquad \text {and } \quad \check{b}^*_x = \check{a}^*_x \, \sqrt{\frac{N-{\mathcal {N}}_+}{N}} \end{aligned}$$

in position space, for $x \in \Lambda$. The commutation relations (2.9) take the form

$$\begin{aligned} \begin{aligned} {[} \check{b}_x, \check{b}_y^* ]&= \left( 1 - \frac{{\mathcal {N}}_+}{N} \right) \delta (x-y) - \frac{1}{N} \check{a}_y^* \check{a}_x \\ {[} \check{b}_x, \check{b}_y ]&= [ \check{b}_x^* , \check{b}_y^*] = 0 \end{aligned} \end{aligned}$$

Moreover, (2.10) translates to

$$\begin{aligned} \begin{aligned} {[}\check{b}_x, \check{a}_y^* \check{a}_z]&=\delta (x-y)\check{b}_z, \qquad {[}\check{b}_x^*, \check{a}_y^* \check{a}_z] = -\delta (x-z) \check{b}_y^* \end{aligned} \end{aligned}$$

which also implies that $[ \check{b}_x, {\mathcal {N}}_+ ] = \check{b}_x$, $[ \check{b}_x^* , {\mathcal {N}}_+ ] = - \check{b}_x^*$.

## Renormalized Excitation Hamiltonian

Conjugation with $U_N$ extracts, from the original quartic interaction in (2.3), some constant and some quadratic contributions, collected in ${\mathcal {L}}^{(0)}_N$ and ${\mathcal {L}}^{(2)}_N$ in (2.7). For bosons described by the Hamiltonian (1.1), this is not enough; there are still large contributions to the energy that are hidden in ${\mathcal {L}}^{(3)}_N$ and ${\mathcal {L}}^{(4)}_N$.

To extract the missing energy, we have to take into account correlations. To this end, we consider the ground state solution $f_\ell$ of the Neumann problem

3.1 $$\begin{aligned} \left[ -\Delta + \frac{1}{2} V \right] f_{\ell } = \lambda _{\ell } f_\ell \end{aligned}$$

on the ball $|x| \le N^{1-\kappa }\ell$ (we omit the $N\in {\mathbb {N}}$-dependence in the notation for $f_\ell$ and for $\lambda _\ell$; notice that $\lambda _\ell$ scales as $N^{3\kappa -3}$), with the normalization $f_\ell (x) = 1$ if $|x| = N^{1-\kappa } \ell$. By scaling, we observe that $f_\ell (N^{1-\kappa }.)$ satisfies the equation

$$\begin{aligned} \left[ -\Delta + \frac{1}{2} N^{2-2\kappa } V (N^{1-\kappa }x) \right] f_\ell (N^{1-\kappa }x) = N^{2-2\kappa } \lambda _\ell f_\ell (N^{1-\kappa }x) \end{aligned}$$

on the ball $|x| \le \ell$. From now on, we fix some $0< \ell < 1/2$, so that the ball of radius $\ell$ is contained in the box $\Lambda = [-1/2 ; 1/2]^3$. We then extend $f_\ell (N^{1-\kappa }.)$ to $\Lambda$, by setting $f_{N} (x) = f_\ell (N^{1-\kappa }x)$, if $|x| \le \ell$ and $f_{N} (x) = 1$ for $x \in \Lambda$, with $|x| > \ell$. As a consequence,

3.2 $$\begin{aligned} \left[ -\Delta + \frac{1}{2}N^{2-2\kappa } V (N^{1-\kappa }.) \right] f_{N} = N^{2-2\kappa } \lambda _\ell f_{N} \chi _\ell , \end{aligned}$$

where $\chi _\ell$ denotes the characteristic function of the ball of radius $\ell$. The Fourier coefficients of the function $f_{N}$ are given by

3.3 $$\begin{aligned} {\widehat{f}}_{N} (p) = \int _\Lambda f_\ell (N^{1-\kappa }x) e^{-i p \cdot x} dx \end{aligned}$$

for all $p \in \Lambda ^*$. Next, we define $w_\ell (x) = 1- f_\ell (x)$ for $|x| \le N^{1-\kappa } \ell$ and $w_\ell (x) = 0$ for all $|x| > N^{1-\kappa } \ell$. Its rescaled version $w_{N} : \Lambda \rightarrow {\mathbb {R}}$ is defined through $w_{N} (x) = w_{\ell } (N^{1-\kappa }x)$ if $|x| \le \ell$ and $w_{N} (x) = 0$ if $x \in \Lambda$ with $|x| > \ell$. The Fourier coefficients of $w_{N}$ are given by

$$\begin{aligned} {\widehat{w}}_{N} (p) = \int _{\Lambda } w_\ell (N^{1-\kappa }x) e^{-i p \cdot x} dx = \frac{1}{N^{3-3\kappa }} {\widehat{w}}_\ell (p/N^{1-\kappa }), \end{aligned}$$

where

$$\begin{aligned} {\widehat{w}}_\ell (k) = \int _{{\mathbb {R}}^3} w_\ell (x) e^{-ik \cdot x} dx \end{aligned}$$

denotes the Fourier transform of the (compactly supported) function $w_\ell$. We find ${\widehat{f}}_{N} (p) = \delta _{p,0} - N^{3\kappa -3} {\widehat{w}}_\ell (p/N^{1-\kappa })$. From (3.2), we obtain

3.4 $$\begin{aligned} \begin{aligned}&- p^2 {\widehat{w}}_\ell (p/N^{1-\kappa }) + \frac{N^{2-2\kappa }}{2} \sum _{q \in \Lambda ^*} {\widehat{V}} ((p-q)/N^{1-\kappa }) {\widehat{f}}_{N} (q) \\&\quad = N^{5-5\kappa } \lambda _\ell \sum _{q \in \Lambda ^*} {\widehat{\chi }}_\ell (p-q) {\widehat{f}}_{N} (q). \end{aligned} \end{aligned}$$

The next lemma summarizes important properties of the functions $w_\ell$ and $f_\ell$. Its proof can be found in [4, Appendix A] (replacing $N\in {\mathbb {N}}$ by $N^{1-\kappa }$ and noting that still $N^{1-\kappa }\ell \gg 1$ for $N\in {\mathbb {N}}$ sufficiently large and fixed $\ell \in (0;1/2)$).

### Lemma 3.1

Let $V \in L^3 ({\mathbb {R}}^3)$ be nonnegative, compactly supported and spherically symmetric. Fix $\ell > 0$ and let $f_\ell$ denote the solution of (3.1). For $N\in {\mathbb {N}}$ large enough, the following properties hold true.

(i) We have
  3.5 $$\begin{aligned} \lambda _\ell = \frac{3\mathfrak {a}_0 }{ N^{3-3\kappa }\ell ^3} \left( 1 +{\mathcal {O}} \big (\mathfrak {a}_0 / \ell N^{1-\kappa }\big ) \right) . \end{aligned}$$
(ii) We have $0\le f_\ell , w_\ell \le 1$. Moreover there exists a constant $C > 0$ such that
  3.6 $$\begin{aligned} \left| \int V(x) f_\ell (x) dx - 8\pi \mathfrak {a}_0 \right| \le \frac{C \mathfrak {a}_0^2}{\ell N^{1-\kappa }}. \end{aligned}$$
(iii) There exists a constant $$C>0 $$ such that
  3.7 $$\begin{aligned} w_\ell (x)\le \frac{C}{|x|+1} \quad \text { and }\quad |\nabla w_\ell (x)|\le \frac{C }{x^2+1}. \end{aligned}$$
  for all $x \in {\mathbb {R}}^3$ and all $N\in {\mathbb {N}}$ large enough.
(iv) There exists a constant $C > 0$ such that
  $$\begin{aligned} |{\widehat{w}}_{N} (p)| \le \frac{C}{N^{1-\kappa } p^2} \end{aligned}$$
  for all $p \in {\mathbb {R}}^3$ and all $N\in {\mathbb {N}}$ large enough (such that $N^{1-\kappa } \ge \ell ^{-1}$).

We define $\eta : \Lambda ^* \rightarrow {\mathbb {R}}$ through

3.8 $$\begin{aligned} \eta _p = -N {\widehat{w}}_{N} (p) = - \frac{N^\kappa }{N^{2-2\kappa }} {\widehat{\omega }}_\ell (p/N^{1-\kappa }). \end{aligned}$$

In position space, this means that for $x\in \Lambda$, we have

3.9 $$\begin{aligned} \check{\eta }(x) = - N w_\ell ( N^{1-\kappa } x), \end{aligned}$$

so that we have in particular the $L^\infty$-bound

3.10 $$\begin{aligned} \Vert \check{\eta }\Vert _\infty \le CN. \end{aligned}$$

Lemma 3.1 also implies

3.11 $$\begin{aligned} |\eta _p| \le \frac{CN^{\kappa }}{|p|^2} \end{aligned}$$

for all $p \in \Lambda _+^*=2\pi {\mathbb {Z}}^3 \backslash \{0\}$, and for some constant $C>0$ independent of $N\in {\mathbb {N}}$ (for $N\in {\mathbb {N}}$ large enough). From (3.4), we find the relation

3.12 $$\begin{aligned} \begin{aligned} p^2 \eta _p + \frac{1}{2} N^{\kappa }({\widehat{V}} (./N^{1-\kappa }) *{\widehat{f}}_{N}) (p) = N^{3-2\kappa } \lambda _\ell ({\widehat{\chi }}_\ell * {\widehat{f}}_{N}) (p) \end{aligned} \end{aligned}$$

or equivalently, expressing the r.h.s. through the coefficients $\eta _p$,

3.13 $$\begin{aligned} \begin{aligned}&p^2 \eta _p + \frac{1}{2} N^{\kappa }{\widehat{V}} (p/N^{1-\kappa }) + \frac{1}{2N} \sum _{q \in \Lambda ^*} N^{\kappa }{\widehat{V}} ((p-q)/N^{1-\kappa }) \eta _q \\&\quad = N^{3-2\kappa } \lambda _\ell {\widehat{\chi }}_\ell (p) + N^{2-2\kappa } \lambda _\ell \sum _{q \in \Lambda ^*} {\widehat{\chi }}_\ell (p-q) \eta _q. \end{aligned} \end{aligned}$$

In our analysis, it is useful to restrict $\eta$ to high momenta. To this end, let $\alpha > 0$ and

3.14 $$\begin{aligned} P_{H}= \{p\in \Lambda _+^*: |p|\ge N^{\alpha }\}. \end{aligned}$$

We define $\eta _H\in \ell ^2(\Lambda _+^*)$ by

3.15 $$\begin{aligned} \eta _H (p)=\eta _p\, \chi (p \in P_H) = \eta _p \chi (|p| \ge N^{\alpha }) \,. \end{aligned}$$

Equation (3.11) implies that

3.16 $$\begin{aligned} \Vert \eta _H \Vert \le C N^{\kappa -\alpha /2} \end{aligned}$$

and we assume from now on that $\alpha > 2\kappa$ such that in particular

3.17 $$\begin{aligned} \lim _{N\rightarrow \infty }\Vert \eta _H \Vert = 0. \end{aligned}$$

Notice, on the other hand, that the $H^1$-norm of $\eta$ and $\eta _{H}$ diverge, as $N \rightarrow \infty$. From (3.9) and Lemma 3.1, part iii), we find

3.18 $$\begin{aligned} \sum _{p \in P_H} p^2 |\eta _p|^2 \le \sum _{p \in \Lambda _+^*} p^2 |\eta _p|^2 = \int |\nabla \check{\eta } (x)|^2 dx \le C N^{1+\kappa } \end{aligned}$$

for all $N \in {\mathbb {N}}$ large enough. We will mostly use the coefficients $\eta _p$ with $p\ne 0$. Sometimes, however, it will be useful to have an estimate on $\eta _0$ (because Eq. (3.13) involves $\eta _0$). From Lemma 3.1, part iii), we obtain

3.19 $$\begin{aligned} |\eta _0| \le N^{3\kappa -2} \int _{{\mathbb {R}}^3} w_\ell (x) dx \le C N^{\kappa }\ell ^2 \end{aligned}$$

It will also be useful to have bounds for the function $\check{\eta }_H : \Lambda \rightarrow {\mathbb {R}}$, having Fourier coefficients $\eta _H (p)$ as defined in (3.15). Writing $\eta _H (p) = \eta _p - \eta _p \chi (|p| \le N^{\alpha })$, we obtain

$$\begin{aligned} \check{\eta }_H (x) = \check{\eta } (x) - \sum _{\begin{array}{c} p \in \Lambda ^* :\\ |p| \le N^{\alpha } \end{array}} \eta _p e^{i p \cdot x} = -N w_\ell (N^{1-\kappa }x) - \sum _{\begin{array}{c} p \in \Lambda ^* :\\ |p| \le N^{\alpha } \end{array}} \eta _p e^{i p \cdot x} \end{aligned}$$

so that

3.20 $$\begin{aligned} |\check{\eta }_H (x)| \le C N + CN^{\kappa }\sum _{\begin{array}{c} p \in \Lambda ^* :\\ |p| \le N^{\alpha } \end{array}} |p|^{-2} \le C (N + N^{\alpha +\kappa }) \le C (N + N^{\alpha +\kappa }) \end{aligned}$$

for all $x \in \Lambda$, if $N \in {\mathbb {N}}$ is large enough.

With the coefficients (3.15), we define the antisymmetric operator

3.21 $$\begin{aligned} B = \frac{1}{2} \sum _{p \in P_H} \left( \eta _p b_p^* b_{-p}^* - {\bar{\eta }}_p b_{-p} b_p \right) \end{aligned}$$

and the generalized Bogoliubov transformation $e^B : {\mathcal {F}}_+^{\le N} \rightarrow {\mathcal {F}}_+^{\le N}$. A first important observation is that conjugation with this unitary operator does not change the number of particles by too much. The proof of the following Lemma can be found in [7, Lemma 3.1] (a similar result has been previously established in [22]).

### Lemma 3.2

Assume *B* is defined as in (3.21), with the coefficients $\eta _p$ as in (3.8), satisfying (3.17). For every $n \in {\mathbb {N}}$, there exists a constant $C > 0$ such that

3.22 $$\begin{aligned} e^{-B} ({\mathcal {N}}_+ +1)^{n} e^{B} \le C ({\mathcal {N}}_+ +1)^{n} \end{aligned}$$

as an operator inequality on ${\mathcal {F}}_+^{\le N}$. (The constant depends only on $\Vert \eta _H \Vert$ and on $n \in {\mathbb {N}}$.)

With the generalized Bogoliubov transformation $e^B$, we can now define the renormalized excitation Hamiltonian ${\mathcal {G}}_{N} : {\mathcal {F}}^{\le N}_+ \rightarrow {\mathcal {F}}^{\le N}_+$ by setting

3.23 $$\begin{aligned} {\mathcal {G}}_{N} = e^{-B} {\mathcal {L}}_N e^{B} = e^{-B} U_N H_N U_N^* e^{B}. \end{aligned}$$

In the next propositions, we collect important properties of ${\mathcal {G}}_{N}$. Recall the notation ${\mathcal {H}}_N = {\mathcal {K}}+ {\mathcal {V}}_N$, introduced in (1.9).

### Proposition 3.3

Let $V\in L^3({\mathbb {R}}^3)$ be compactly supported, pointwise nonnegative and spherically symmetric. Let ${\mathcal {G}}_N$ be defined as in (3.23). Assume that the exponent $\alpha$ introduced in (3.14) is such that

3.24 $$\begin{aligned} \alpha > 6 \kappa , \qquad 2\alpha + 3 \kappa < 1 \end{aligned}$$

Then,

3.25 $$\begin{aligned} {\mathcal {G}}_{N} = 4 \pi \mathfrak {a}_0 N^{1+\kappa } + {\mathcal {H}}_N + \theta _{{\mathcal {G}}_{N}} \end{aligned}$$

and there exists $C>0$ such that, for all $\delta > 0$ and all $N \in {\mathbb {N}}$ large enough, we have

3.26 $$\begin{aligned} \pm \theta _{{\mathcal {G}}_{N}} \le \delta {\mathcal {H}}_N + C \delta ^{-1}N^{\alpha +2\kappa } {\mathcal {N}}_+ + CN^{\alpha +2\kappa } \end{aligned}$$

and the improved lower bound

3.27 $$\begin{aligned} \theta _{{\mathcal {G}}_{N}} \ge - \delta {\mathcal {H}}_N - C \delta ^{-1} N^{\kappa } {\mathcal {N}}_+ - C N^{\alpha +2\kappa }. \end{aligned}$$

Furthermore, for $\beta >0$, denote by ${\mathcal {G}}_N^{\text {eff}}$ the excitation Hamiltonian

3.28 $$\begin{aligned} \begin{aligned} {\mathcal {G}}^{\text {eff}}_{N} =&\;4\pi \mathfrak {a}_0 N^{\kappa } (N-{\mathcal {N}}_+) + \big [{{\widehat{V}}}(0)-4\pi \mathfrak {a}_0\big ]N^{\kappa }{\mathcal {N}}_+\frac{(N-{\mathcal {N}}_+)}{N}\\&+ N^{\kappa }{{\widehat{V}}}(0) \sum _{p\in P_H^c} a^*_pa_p (1-{\mathcal {N}}_+/N) + 4\pi \mathfrak {a}_0 N^{\kappa }\sum _{p\in P_H^c} \big [ b^*_p b^*_{-p} + b_p b_{-p} \big ] \\&+ \frac{1}{\sqrt{N}}\sum _{\begin{array}{c} p,q\in \Lambda _+^*: |q|\le N^{\beta },\\ p+q\ne 0 \end{array}} N^{\kappa }{{\widehat{V}}}(p/N^{1-\kappa })\big [ b^*_{p+q}a^*_{-p}a_q+ h.c. \big ]+{\mathcal {H}}_N \end{aligned} \end{aligned}$$

Then, there exists $C > 0$ such that ${\mathcal {E}}_{{\mathcal {G}}_{N}} = {\mathcal {G}}_{N} - {\mathcal {G}}^{\text {eff}}_{N}$ is bounded by

3.29 $$\begin{aligned} \pm {\mathcal {E}}_{{\mathcal {G}}_{N}} \le C (N^{3\kappa -\alpha /2} + N^{\alpha +3\kappa /2-1/2} + N^{\kappa /2-\beta }) {\mathcal {H}}_N + CN^{\alpha +2\kappa } \end{aligned}$$

for all $N\in {\mathbb {N}}$ sufficiently large.

Furthermore, there exists a constant $C > 0$ such that

3.30 $$\begin{aligned} \begin{aligned} \pm i[{\mathcal {N}}_{\ge cN^{\gamma }}, {\mathcal {G}}_{N}], \;\pm i [{\mathcal {N}}_{< cN^{\gamma }}, {\mathcal {G}}_{N}]&\le C ( N^{\kappa +\alpha /2 -\gamma } +N^{\kappa +\gamma /2 } ) ({\mathcal {H}}_N+1) \end{aligned} \end{aligned}$$

for all $\alpha \ge \gamma >0$, $c>0$ fixed (independent of $N\in {\mathbb {N}}$) and $N\in {\mathbb {N}}$ large enough.

Finally, for every $k\in {\mathbb {N}}$, there exists a constant $C>0$ such that

3.31 $$\begin{aligned} \pm {\text {ad}}^{(k)}_{i{\mathcal {N}}_+}({\mathcal {G}}_N) = \pm \big [ i{\mathcal {N}}_+, \ldots \big [i{\mathcal {N}}_+, {\mathcal {G}}_N\big ]\ldots \big ]\le C N^{\kappa +\alpha /6 } ({\mathcal {H}}_N+1). \end{aligned}$$

The proof of Proposition 3.3 is similar to the proof of [4, Prop. 4.2] and [3, Prop. 3.2], with the appropriate modifications dictated by the different scaling of the interaction. The main novelty in Proposition 3.3 is the bound (3.30) involving commutators of the restricted number of particles operator ${\mathcal {N}}_{\ge c N^\gamma }$. This can be obtained similarly to the bounds for ${\mathcal {E}}_{{\mathcal {G}}_N}$ and for $i [ {\mathcal {N}}_+ , {\mathcal {G}}_N ]$, because we have a full expansion of the operator ${\mathcal {G}}_N$ in a sum of terms whose commutators with ${\mathcal {N}}_+$ and with ${\mathcal {N}}_{\ge cN^\gamma }$ retains essentially the same form. In the version of this paper that is posted on the arXiv, we give a complete proof of Proposition 3.3 in “Appendix A”, adapting the arguments of [4, Prop. 4.2], [3, Prop. 3.2].

## Cubic Renormalization

From Eq. (3.28), we observe that the cubic terms in ${\mathcal {G}}^\text {eff}_N$ still depend on the original interaction, which decays slowly in momentum (in contrast to the quadratic terms in the second line of (3.28), where the sum is now restricted to $P_H^c = \{ p \in \Lambda ^*_+ : |p| < N^\alpha \}$).

To renormalize the cubic terms in (3.28), we are going to conjugate ${\mathcal {G}}^\text {eff}_N$ with a unitary operator $e^A$, where the antisymmetric operator $A:{\mathcal {F}}_+^{\le N}\rightarrow {\mathcal {F}}_+^{\le N}$ is defined by

4.1 $$\begin{aligned} \begin{aligned} A&= A_1-A_1^*, \text {with } A_1=\frac{1}{\sqrt{N}} \sum _{r\in P_{H}, p \in P_{L}} \eta _r b^*_{r+p}a^*_{-r}a_p . \end{aligned} \end{aligned}$$

The high-momentum set $P_H = \{ p \in \Lambda ^*_+ : |p| \ge N^\alpha \}$ is as in (3.14). The low-momentum set $P_L$ is defined by

4.2 $$\begin{aligned} P_L = \{ p \in \Lambda _+^* : |p| \le N^{\beta } \} \end{aligned}$$

with exponent $\beta >0$, that will be chosen as in (3.28).

Using the unitary operator $e^A$, we define ${\mathcal {J}}_N:{\mathcal {F}}_+^{\le N}\rightarrow {\mathcal {F}}_+^{\le N}$ by

4.3 $$\begin{aligned} {\mathcal {J}}_N = e^{-A} {\mathcal {G}}_N^{\text {eff}}e^A. \end{aligned}$$

Observe here that we only conjugate the main part ${\mathcal {G}}_N^\text {eff}$ of the renormalized excitation Hamiltonian ${\mathcal {G}}_N$; this makes the analysis a bit simpler (the difference ${\mathcal {G}}_N - {\mathcal {G}}_N^\text {eff}$ is small and can be estimated before applying the cubic conjugation).

The next proposition summarizes important properties of ${\mathcal {J}}_N$; it can be shown very similarly to [4, Prop. 5.2], of course with the appropriate changes of the scaling of the interaction. In the version of this paper that is posted on the arXiv, we give a complete proof of Proposition 4.1 in “Appendix B”, adapting the arguments of [4, Prop. 5.2].

### Proposition 4.1

Suppose the exponents $\alpha$ and $\beta$ are such that

4.4 $$\begin{aligned} i) \; \alpha> 3 \beta + 2\kappa , \quad ii) \; 3\alpha /2 + 2 \kappa< 1, \quad iii) \; \alpha< 5 \beta , \quad iv) \; \beta > 3\kappa /2, \quad v) \; \beta < 1/2 \end{aligned}$$

Let ${\mathcal {J}}_N$ be defined as in (4.3), let

4.5 $$\begin{aligned} \begin{aligned} {\mathcal {J}}_{N}^{\text {eff}} =&\;4\pi \mathfrak {a}_0 N^{1+\kappa } - 4\pi \mathfrak {a}_0 N^{\kappa }{\mathcal {N}}_+^2/N + 8\pi {\mathfrak {a}}_0 N^{\kappa } \sum _{p\in P_H^c} \Big [ b^*_pb_p + \frac{1}{2} b^*_p b^*_{-p} + \frac{1}{2} b_p b_{-p} \Big ] \\&+ \frac{8\pi {\mathfrak {a}}_0N^{\kappa }}{\sqrt{N}}\sum _{\begin{array}{c} p \in P_H^c,q\in P_L: \\ p+q\ne 0 \end{array}} \big [ b^*_{p+q}a^*_{-p}a_q+ h.c. \big ]+{\mathcal {H}}_N, \end{aligned} \end{aligned}$$

and set $\mu = \max (3\alpha /2 +2\kappa -1, 3\kappa /2-\beta )$ ($\mu < 0$ follows from (4.4)). Then, there exists a constant $C > 0$ such that the self-adjoint operator ${\mathcal {E}}_{{\mathcal {J}}_{N}} = {\mathcal {J}}_N - {\mathcal {J}}_N^{\text {eff} }$ satisfies the operator inequality

4.6 $$\begin{aligned} \pm e^A{\mathcal {E}}_{{\mathcal {J}}_N}e^{-A} \le C(N^{-\beta /2}+ N^{\mu } ){\mathcal {K}}+ CN^{\mu }{\mathcal {V}}_N + CN^{\mu -\kappa }{\mathcal {N}}_+ +CN^{\alpha +2\kappa }(1+ N^{\alpha +\beta /2-1}) \end{aligned}$$

in ${\mathcal {F}}_+^{\le N}$ for all $N\in {\mathbb {N}}$ sufficiently large.

The bounds for ${\mathcal {J}}_N$ given in Proposition 4.1 are still not enough to show Theorem 1.1. As we will discuss in the next section, the main problem is the quartic interaction term, contained in ${\mathcal {H}}_N$, which still depends on the singular interaction potential (in all other terms on the r.h.s. of (4.5), the singular potential has been replaced by the regular mean-field type potential, with Fourier transform $8\pi \mathfrak {a}_0 N^\kappa \mathbf{1 }_{P_H^c} (p)$, supported on momenta $|p| < N^\alpha$). To renormalize the quartic interaction, we will have to conjugate ${\mathcal {J}}^\text {eff}_N$ with yet another unitary operator, this time quartic in creation and annihilation operators. This last conjugation (which will be performed in the next section) will produce error terms. These errors will controlled in terms of the observables ${\mathcal {N}}_+$, ${\mathcal {K}}$ and ${\mathcal {V}}_N$ (as in (4.6)) but also, as we stressed at the end of Sect. 1, in terms of observables having the form ${\mathcal {N}}_{\ge N^\gamma }$ (the number of excitations having momentum larger or equal to $N^\gamma$), ${\mathcal {N}}^2_{\ge N^\gamma }$, ${\mathcal {N}}_{\ge N^\gamma }^3$, ${\mathcal {K}}_{\le N^\gamma }$ (the kinetic energy of excitations with momentum below $N^\gamma$), ${\mathcal {K}}_L {\mathcal {N}}_{\ge N^\gamma }$. For this reason, we need to control the action of $e^A$ on all these observables.

First of all, we bound the action of the cubic phase on the restricted number of particles operators ${\mathcal {N}}_{\ge \theta } = \sum _{p \in \Lambda ^*_+ : |p| \ge \theta } a_p^* a_p$. We will make use of the pull-through formula $a_p {\mathcal {N}}_{\ge \theta } = ({\mathcal {N}}_{\ge \theta } + \mathbf{1 }_{[\theta , \infty )} (p)) a_p$, which in particular implies that

4.7 $$\begin{aligned} \begin{aligned} \Vert ({\mathcal {N}}_{\ge \theta } +1)^{1/2} a_p \xi \Vert&\le C \Vert a_p ({\mathcal {N}}_{\ge \theta } +1)^{1/2}\xi \Vert , \\ \Vert ({\mathcal {N}}_{\ge \theta } +1)^{-1/2} a_p \xi \Vert&\le C \Vert a_p ({\mathcal {N}}_{\ge \theta } +1)^{-1/2}\xi \Vert \, . \end{aligned} \end{aligned}$$

### Lemma 4.2

Assume the exponents $\alpha , \beta$ satisfy (4.4) (in fact, here it is enough to assume that $\alpha > 2\kappa$). Let $k\in {\mathbb {N}}_0$, $m=0,1,2$, $0 < \gamma \le \alpha$, $c \ge 0$ (and $c < 1$ if $\gamma =\alpha$). Then, there exists a constant $C>0$ such that the operator inequalities

4.8 $$\begin{aligned} \begin{aligned} e^{-sA} ({\mathcal {N}}_+ + 1)^{k }({\mathcal {N}}_{\ge c N^{\gamma }}+1)^m e^{sA} \le&\; C({\mathcal {N}}_+ + 1)^{k } ({\mathcal {N}}_{\ge cN^{\gamma }}+1)^m \end{aligned} \end{aligned}$$

for all $s\in [-1;1]$ and all $N\in {\mathbb {N}}$.

### Proof

The case $m=0$ follows from $m=1$. We start therefore with the case $m=1$. For $\xi \in {\mathcal {F}}_+^{\le N}$, we define the function $\varphi _\xi :{\mathbb {R}}\rightarrow {\mathbb {R}}$ by

$$\begin{aligned} \varphi _\xi (s) = \langle \xi , e^{-sA} ({\mathcal {N}}_+ + 1)^{k }({\mathcal {N}}_{\ge c N^{\gamma }}+1) e^{sA} \xi \rangle \end{aligned}$$

which has derivative

4.9 $$\begin{aligned} \begin{aligned} \partial _s \varphi _\xi (s)&=2 \mathrm{Re}\,\langle e^{sA} \xi , ({\mathcal {N}}_+ + 1)^{k}\big [{\mathcal {N}}_{\ge c N^{\gamma }}, A_1 \big ] e^{sA} \xi \rangle \\&\quad + 2\mathrm{Re}\,\langle e^{sA} \xi , \big [({\mathcal {N}}_+ + 1)^{k}, A_1 \big ]({\mathcal {N}}_{\ge c N^{\gamma }}+1) e^{sA} \xi \rangle , \end{aligned} \end{aligned}$$

where $A_1$ as in (4.1). By the assumptions on $\gamma$ and *c*, we have $N^\alpha \ge N^{\alpha }-N^{\beta } \ge cN^{\gamma }$ for $N\in {\mathbb {N}}$ large enough. This implies in particular that

$$\begin{aligned} {[}{\mathcal {N}}_{\ge cN^{\gamma } }, b^*_{p+r}] = b^*_{p+r},  [{\mathcal {N}}_{\ge cN^{\gamma } }, a^*_{-r}] = a^*_{-r},  [{\mathcal {N}}_{\ge cN^\gamma }, a_p] = \chi (|p|\ge cN^\gamma ) a_p \end{aligned}$$

for $r\in P_H$ and $p\in P_L$, by (2.1) and (2.10). We then obtain

4.10 $$\begin{aligned} \begin{aligned} \big [{\mathcal {N}}_{\ge c N^{\gamma }}, A_1 \big ]&= \frac{2}{\sqrt{N}} \sum _{\begin{array}{c} r\in P_{H}, p \in P_{L} \end{array}} \eta _r b^*_{r+p}a^*_{-r}a_p - \frac{1}{\sqrt{N}} \sum _{\begin{array}{c} r\in P_{H}, p \in P_{L},\\ |p| \ge cN^{\gamma } \end{array}} \eta _r b^*_{r+p}a^*_{-r}a_p \end{aligned} \end{aligned}$$

as well as

4.11 $$\begin{aligned} \begin{aligned} \big [({\mathcal {N}}_++1)^k, A_1 \big ]&= \frac{k}{\sqrt{N}} \sum _{r\in P_{H}, p \in P_{L}} \eta _r b^*_{r+p}a^*_{-r}a_p ({\mathcal {N}}_++\Theta ({\mathcal {N}}_+)+1)^{k-1}, \end{aligned} \end{aligned}$$

for some function $\Theta : {\mathbb {N}}\rightarrow (0;1)$ by the mean value theorem. Using the pull-through formula ${\mathcal {N}}_+ a^*_p = a^*_p ({\mathcal {N}}_++1)$ and Cauchy–Schwarz, we estimate

$$\begin{aligned} \begin{aligned}&\bigg | \frac{1}{\sqrt{N}} \sum _{\begin{array}{c} r\in P_{H}, p \in P_{L} \end{array}} \eta _r \langle e^{sA}\xi , ({\mathcal {N}}_+ + 1)^{k} b^*_{r+p}a^*_{-r}a_p e^{sA}\xi \rangle \bigg | \\&\quad \le \frac{1}{\sqrt{N}} \bigg ( \sum _{\begin{array}{c} r\in P_{H}, p \in P_{L} \end{array}} \Vert ({\mathcal {N}}_{\ge cN^{\gamma } }+1)^{-1/2} a_{r+p}a_{-r} ({\mathcal {N}}_+ + 1)^{k/2}e^{sA}\xi \Vert ^2 \bigg )^{1/2}\\&\qquad \times \bigg ( \sum _{\begin{array}{c} r\in P_{H}, p \in P_{L} \end{array}} \eta _r^2\Vert ({\mathcal {N}}_{\ge cN^{\gamma } }+1)^{1/2} a_p ({\mathcal {N}}_+ + 1)^{k/2} e^{sA}\xi \Vert ^2\bigg )^{1/2} \end{aligned}\end{aligned}$$

With the operator inequality ${\mathcal {N}}_{\ge cN^{\gamma } } \ge {\mathcal {N}}_{\ge N^{\alpha }}$ and with (4.7), we find that

4.12 $$\begin{aligned} \begin{aligned}&\bigg | \frac{1}{\sqrt{N}} \sum _{\begin{array}{c} r\in P_{H}, p \in P_{L} \end{array}} \eta _r \langle e^{sA}\xi , ({\mathcal {N}}_+ + 1)^{k} b^*_{r+p}a^*_{-r}a_p e^{sA}\xi \rangle \bigg | \\&\quad \le \frac{C}{\sqrt{N}} \bigg ( \sum _{\begin{array}{c} r\in P_{H}, p \in P_{L}:|p+r|\ge cN^{\gamma } \end{array}} \Vert a_{p+r} ({\mathcal {N}}_{\ge cN^{\gamma } }+1)^{-1/2} a_{-r} ({\mathcal {N}}_+ + 1)^{k/2}e^{sA}\xi \Vert ^2 \bigg )^{1/2}\\&\qquad \times \Vert \eta _H\Vert \bigg ( \sum _{\begin{array}{c} p \in P_{L} \end{array} } \Vert a_p({\mathcal {N}}_{\ge cN^{\gamma } }+1)^{1/2} ({\mathcal {N}}_+ + 1)^{k/2} e^{sA}\xi \Vert ^2\bigg )^{1/2} \\&\quad \le \frac{CN^{\kappa -\alpha /2}}{\sqrt{N}} \Vert ({\mathcal {N}}_{\ge N^{\alpha } }+1)^{1/2} ({\mathcal {N}}_+ + 1)^{k/2} e^{sA}\xi \Vert \Vert ({\mathcal {N}}_{\ge cN^{\gamma } }+1)^{1/2} ({\mathcal {N}}_+ + 1)^{(k+1)/2} e^{sA}\xi \Vert \\&\quad \le CN^{\kappa -\alpha /2}\Vert ({\mathcal {N}}_{\ge cN^{\gamma } }+1)^{1/2} ({\mathcal {N}}_+ + 1)^{k/2} e^{sA}\xi \Vert ^2 = CN^{\kappa -\alpha /2} \varphi _\xi (s). \end{aligned} \end{aligned}$$

The same arguments show that

4.13 $$\begin{aligned} \begin{aligned}&\bigg | \frac{1}{\sqrt{N}} \sum _{\begin{array}{c} r\in P_{H}, p \in P_{L},\\ |p|\ge cN^{\gamma } \end{array}} \eta _r \langle e^{sA}\xi , ({\mathcal {N}}_+ + 1)^{k} b^*_{r+p}a^*_{-r}a_p e^{sA}\xi \rangle \bigg | \\&\quad \le \frac{C}{\sqrt{N}} \bigg ( \sum _{\begin{array}{c} r\in P_{H}, p \in P_{L}:|p+r|\ge cN^{\gamma } \end{array}} \Vert a_{p+r} ({\mathcal {N}}_{\ge cN^{\gamma } }+1)^{-1/2} a_{-r} ({\mathcal {N}}_+ + 1)^{k/2}e^{sA}\xi \Vert ^2 \bigg )^{1/2}\\&\qquad \times \Vert \eta _H\Vert \bigg ( \sum _{\begin{array}{c} p \in P_{L} \end{array} } \Vert a_p({\mathcal {N}}_{\ge cN^{\gamma } }+1)^{1/2} ({\mathcal {N}}_+ + 1)^{k/2} e^{sA}\xi \Vert ^2\bigg )^{1/2} \\&\quad \le CN^{\kappa -\alpha /2} \varphi _\xi (s). \end{aligned} \end{aligned}$$

Finally, we have that

4.14 $$\begin{aligned} \begin{aligned}&\bigg | \frac{k}{\sqrt{N}} \sum _{r\in P_{H}, p \in P_{L}} \eta _r \langle e^{sA}\xi ,b^*_{r+p}a^*_{-r}a_p ({\mathcal {N}}_++\Theta ({\mathcal {N}}_+)+1)^{k-1}({\mathcal {N}}_{\ge cN^{\gamma }} +1)e^{sA}\xi \rangle \bigg | \\&\quad \le \frac{C}{\sqrt{N}} \bigg ( \sum _{\begin{array}{c} r\in P_{H}, p \in P_{L}:|p+r|\ge cN^{\gamma } \end{array}} \Vert a_{r+p}a_{-r} ({\mathcal {N}}_+ + 1)^{(k-1)/2}e^{sA}\xi \Vert ^2 \bigg )^{1/2}\\&\quad \quad \times \bigg ( \sum _{\begin{array}{c} r\in P_{H}, p \in P_{L} \end{array}} \eta _r^2\Vert a_p ({\mathcal {N}}_+ + 1)^{(k-1)/2}({\mathcal {N}}_{\ge cN^{\gamma }} +1) e^{sA}\xi \Vert ^2\bigg )^{1/2}\\&\quad \le CN^{\kappa -\alpha /2} \Vert ({\mathcal {N}}_{\ge cN^{\gamma } }+1)^{1/2} ({\mathcal {N}}_+ + 1)^{k/2} e^{sA}\xi \Vert ^2 = CN^{\kappa -\alpha /2} \varphi _\xi (s). \end{aligned}\end{aligned}$$

Recalling (4.9), (4.10) and that $\alpha \ge 2\kappa$, the bounds (4.12) to (4.14) show that

$$\begin{aligned} \partial _s\varphi _\xi (s) \le CN^{\kappa -\alpha /2} \varphi _\xi (s) \le C \varphi _\xi (s). \end{aligned}$$

Since the bounds are independent of $\xi \in {\mathcal {F}}_+^{\le N}$ and the same bounds hold true replacing *A* by $-A$ in the definition of $\varphi _\xi$, the first inequality in (4.8) follows by Gronwall’s Lemma.

To prove (4.8) with $m=2$, we proceed similarly. Given $\xi \in {\mathcal {F}}_+^{\le N}$, we define the function $\psi _\xi :{\mathbb {R}}\rightarrow {\mathbb {R}}$ by

$$\begin{aligned} \psi _\xi (s) = \langle \xi , e^{-sA} ({\mathcal {N}}_+ + 1)^{k }({\mathcal {N}}_{\ge c N^{\gamma }}+1)^2 e^{sA} \xi \rangle . \end{aligned}$$

Its derivative is equal to

4.15 $$\begin{aligned} \begin{aligned} \partial _s \psi _\xi (s)&= 2\mathrm{Re}\,\langle e^{sA} \xi , ({\mathcal {N}}_+ + 1)^{k}\big [({\mathcal {N}}_{\ge c N^{\gamma }}+1)^2, A_1\big ] e^{sA} \xi \rangle \\&\quad + 2\mathrm{Re}\,\langle e^{sA} \xi , \big [({\mathcal {N}}_+ + 1)^{k}, A_1 \big ]({\mathcal {N}}_{\ge c N^{\gamma }}+1)^2 e^{sA} \xi \rangle \\&= 2\mathrm{Re}\,\langle e^{sA} \xi , ({\mathcal {N}}_+ + 1)^{k}\big [{\mathcal {N}}_{\ge c N^{\gamma }}, \big [{\mathcal {N}}_{\ge c N^{\gamma }}, A_1\big ]\big ] e^{sA} \xi \rangle \\&\quad +4\mathrm{Re}\,\langle e^{sA} \xi , ({\mathcal {N}}_+ + 1)^{k}\big [{\mathcal {N}}_{\ge c N^{\gamma }}, A_1\big ]({\mathcal {N}}_{\ge c N^{\gamma }}+1) e^{sA} \xi \rangle \\&\quad + 2\mathrm{Re}\,\langle e^{sA} \xi , \big [({\mathcal {N}}_+ + 1)^{k}, A_1 \big ]({\mathcal {N}}_{\ge c N^{\gamma }}+1)^2 e^{sA} \xi \rangle . \end{aligned}\end{aligned}$$

Comparing the contribution containing the double commutator in the last line on the r.h.s. of the last equation with (4.10) and using once again that $N^\alpha \ge N^{\alpha }-N^{\beta } \ge cN^{\gamma }$ for $N\in {\mathbb {N}}$ large enough, we observe that

4.16 $$\begin{aligned} \begin{aligned} \big [{\mathcal {N}}_{\ge c N^{\gamma }}, \big [{\mathcal {N}}_{\ge c N^{\gamma }}, A_1 \big ] \big ]&= \frac{4}{\sqrt{N}} \sum _{\begin{array}{c} r\in P_{H}, p \in P_{L} \end{array}} \eta _r b^*_{r+p}a^*_{-r}a_p - \frac{3}{\sqrt{N}} \sum _{\begin{array}{c} r\in P_{H}, p \in P_{L},\\ |p| \ge cN^{\gamma } \end{array}} \eta _r b^*_{r+p}a^*_{-r}a_p. \end{aligned} \end{aligned}$$

Hence, the bounds (4.12) and (4.13) prove that

$$\begin{aligned} \big | \langle e^{sA} \xi , ({\mathcal {N}}_+ + 1)^{k}\big [{\mathcal {N}}_{\ge c N^{\gamma }}, \big [{\mathcal {N}}_{\ge c N^{\gamma }}, A_1\big ]\big ] e^{sA} \xi \rangle \big |\le C\varphi _\xi (s) \le C \psi _\xi (s). \end{aligned}$$

To bound the second contribution on the r.h.s. in (4.15), we recall (4.10) and we estimate

$$\begin{aligned} \begin{aligned}&\bigg | \frac{1}{\sqrt{N}} \sum _{\begin{array}{c} r\in P_{H}, p \in P_{L} \end{array}} \eta _r \langle e^{sA}\xi , ({\mathcal {N}}_+ + 1)^{k} b^*_{r+p}a^*_{-r}a_p ({\mathcal {N}}_{\ge cN^\gamma } +1) e^{sA}\xi \rangle \bigg | \\&\quad \quad +\bigg | \frac{1}{\sqrt{N}} \sum _{\begin{array}{c} r\in P_{H}, p \in P_{L},\\ |p|\ge cN^{\gamma } \end{array}} \eta _r \langle e^{sA}\xi , ({\mathcal {N}}_+ + 1)^{k} b^*_{r+p}a^*_{-r}a_p ({\mathcal {N}}_{\ge cN^\gamma } +1) e^{sA}\xi \rangle \bigg | \\&\quad \le \frac{C}{\sqrt{N}} \bigg ( \sum _{\begin{array}{c} r\in P_{H}, p \in P_{L}:|p+r|\ge cN^{\gamma } \end{array}} \Vert a_{p+r} a_{-r} ({\mathcal {N}}_+ + 1)^{k/2}e^{sA}\xi \Vert ^2 \bigg )^{1/2}\\&\qquad \times \Vert \eta _H\Vert \bigg ( \sum _{\begin{array}{c} p \in P_{L} \end{array} } \Vert a_p ({\mathcal {N}}_+ + 1)^{k/2} ({\mathcal {N}}_{\ge cN^\gamma } +1) e^{sA}\xi \Vert ^2\bigg )^{1/2} \\&\quad \le CN^{\kappa -\alpha /2}\Vert ({\mathcal {N}}_{\ge cN^{\gamma } }+1) ({\mathcal {N}}_+ + 1)^{k/2} e^{sA}\xi \Vert ^2 = CN^{\kappa -\alpha /2} \psi _\xi (s) \end{aligned} \end{aligned}$$

Finally, the last contribution in (4.15) can be bounded as in (4.14), using (4.11). We have

$$\begin{aligned} \begin{aligned}&\bigg | \frac{k}{\sqrt{N}} \sum _{r\in P_{H}, p \in P_{L}} \eta _r \langle e^{sA}\xi ,b^*_{r+p}a^*_{-r}a_p ({\mathcal {N}}_++\Theta ({\mathcal {N}}_+)+1)^{k-1}({\mathcal {N}}_{\ge cN^{\gamma }} +1)^2e^{sA}\xi \rangle \bigg | \\&\quad \le \frac{C}{\sqrt{N}} \bigg ( \sum _{\begin{array}{c} r\in P_{H}, p \in P_{L}:|p+r|\ge cN^{\gamma } \end{array}} \Vert a_{r+p}a_{-r} ({\mathcal {N}}_+ + 1)^{k/2}e^{sA}\xi \Vert ^2 \bigg )^{1/2}\\&\qquad \times \bigg ( \sum _{\begin{array}{c} r\in P_{H}, p \in P_{L} \end{array}} \eta _r^2\Vert a_p ({\mathcal {N}}_+ + 1)^{(k-2)/2}({\mathcal {N}}_{\ge cN^{\gamma }} +1)^2 e^{sA}\xi \Vert ^2\bigg )^{1/2}\\&\quad \le CN^{\kappa -\alpha /2} \Vert ({\mathcal {N}}_{\ge cN^{\gamma } }+1) ({\mathcal {N}}_+ + 1)^{k/2} e^{sA}\xi \Vert ^2 = CN^{\kappa -\alpha /2} \psi _\xi (s), \end{aligned} \end{aligned}$$

where, in the last step, we used that ${\mathcal {N}}_{\ge cN^{\gamma }}\le {\mathcal {N}}_+$. In conclusion, we have proved that

$$\begin{aligned} \partial _s \psi _\xi (s)\le CN^{\kappa -\alpha /2} \psi _\xi (s)\le C \psi _\xi (s). \end{aligned}$$

Since the bounds are independent of $\xi \in {\mathcal {F}}_+^{\le N}$ and the same bounds hold true replacing $-A$ by *A* in the definition $\psi _\xi$, Gronwall’s lemma implies the last inequality in (4.8). $\square$

We denote the kinetic energy restricted to low momenta by

4.17 $$\begin{aligned} \begin{aligned} {\mathcal {K}}_{\le cN^{\gamma }} = \sum _{p\in \Lambda _+^*: |p|\le cN^{\gamma }} p^2a^*_pa_p. \end{aligned} \end{aligned}$$

We will need the following estimates for the growth of the restricted kinetic energy.

### Lemma 4.3

Assume the exponents $\alpha ,\beta$ satisfy (4.4) (here we only need $\alpha \ge 2\kappa$ and $\alpha > \beta$). Let $0 < \gamma _1, \gamma _2 \le \alpha$, and $c_1, c_2 \ge 0$ (and also $c_j < 1$, if $\gamma _j = \alpha$, for $j=1,2$). Then, there exists a constant $C>0$ such that the operator inequalities

4.18 $$\begin{aligned} \begin{aligned} e^{-sA} {\mathcal {K}}_{\le c_1 N^{\gamma _1}} e^{sA} \le&\; {\mathcal {K}}_{\le c_1 N^{\gamma _1}} + N^{2\beta +2\kappa -\alpha -1} ({\mathcal {N}}_{\ge \frac{1}{2} N^{\alpha }} +1)^2,\\ e^{-sA} {\mathcal {K}}_{\le c_1 N^{\gamma _1}} ({\mathcal {N}}_{\ge c_2 N^{\gamma _2}}+1)e^{sA} \le&\; {\mathcal {K}}_{\le c_1 N^{\gamma _1}}({\mathcal {N}}_{\ge c_2 N^{\gamma _2}} +1) \\&+ N^{2\beta +2\kappa -\alpha -1} ({\mathcal {N}}_{\ge c_2 N^{\gamma _2}} +1)^2({\mathcal {N}}_{\ge \frac{1}{2} N^{\alpha }} +1)\\ \end{aligned} \end{aligned}$$

for all $s\in [-1;1]$ and all $N\in {\mathbb {N}}$ sufficiently large.

### Proof

Like the previous Lemma 4.2, this is an application of Gronwall’s lemma. Let us start to prove the first inequality in (4.18). Fix $\xi \in {\mathcal {F}}_+^{\le N}$ and define $\varphi _\xi :{\mathbb {R}}\rightarrow {\mathbb {R}}$ by $\varphi _\xi (s) = \langle \xi , e^{-sA} {\mathcal {K}}_{\le c_1 N^{\gamma _1}} e^{sA}\xi \rangle$ such that

$$\begin{aligned}\begin{aligned} \partial _s \varphi _\xi (s)&= 2\mathrm{Re}\,\langle \xi , e^{-sA} [ {\mathcal {K}}_{\le c_1 N^{\gamma _1}} , A_1 ] e^{sA}\xi \rangle .\\ \end{aligned}\end{aligned}$$

We notice first that

$$\begin{aligned} \big [ {\mathcal {K}}_{\le c_1 N^{\gamma _1}}, b^*_{p+r} \big ] = \big [ {\mathcal {K}}_{\le c_1 N^{\gamma _1}}, a^*_{-r} \big ]=0 \end{aligned}$$

if $r\in P_H$ and $p\in P_L$, because $|r|, |p+r| \ge N^{\alpha }- N^{\beta } > c_1 N^{\gamma _1}$ for all $N\in {\mathbb {N}}$.

Using the commutation relations (2.1), we then compute

4.19 $$\begin{aligned} \begin{aligned} {[} {\mathcal {K}}_{\le c_1 N^{\gamma _1}} , A_1 ]&= - \frac{1}{\sqrt{N}} \sum _{\begin{array}{c} r\in P_{H}, p \in P_{L}: |p|\le c_1 N^{\gamma _1} \end{array}} p^2 \eta _r b^*_{r+p}a^*_{-r}a_p. \end{aligned} \end{aligned}$$

With (4.19) and $|p|\le N^{\beta }$ for $p\in P_L$, we then find that

4.20 $$\begin{aligned} \begin{aligned}&\big | \langle \xi , e^{-sA} [ {\mathcal {K}}_{\le c_1 N^{\gamma _1}}, A_1 ] e^{sA}\xi \rangle \big | \\&\quad \le \frac{CN^{\beta }}{\sqrt{N}} \sum _{\begin{array}{c} r\in P_{H}, p \in P_{L}: |p|\le c_1 N^{\gamma _1} \end{array}} |p| |\eta _r| \Vert a_{r+p}a_{-r} e^{sA}\xi \Vert \Vert a_pe^{sA}\xi \Vert \\&\quad \le \frac{CN^{\beta +\kappa -\alpha /2}}{\sqrt{N}} \Vert ({\mathcal {N}}_{\ge \frac{1}{2} N^\alpha }+1) e^{sA}\xi \Vert \Vert {\mathcal {K}}_{\le c_1 N^{\gamma _1}}^{1/2}e^{sA}\xi \Vert . \end{aligned} \end{aligned}$$

Finally, using Lemma 4.2 (with $c=\frac{1}{2}$, $\gamma =\alpha$ and $N\in {\mathbb {N}}$ sufficiently large), we conclude

$$\begin{aligned} \begin{aligned} \partial _s\varphi _\xi (s)&\le CN^{\beta +\kappa -\alpha /2-1/2} \Vert ({\mathcal {N}}_{\ge \frac{1}{2} N^\alpha }+1) e^{sA}\xi \Vert \Vert {\mathcal {K}}_{\le c_1 N^{\gamma _1}}^{1/2}e^{sA}\xi \Vert \\&\le CN^{2\beta +2\kappa -\alpha -1} \langle \xi , ({\mathcal {N}}_{\ge \frac{1}{2} N^\alpha }+1)^2 \xi \rangle + C \varphi _\xi (s). \end{aligned} \end{aligned}$$

This proves the first inequality in (4.18), by Gronwall’s lemma.

Next, let us prove the second inequality in (4.18). We define $\psi _\xi :{\mathbb {R}}\rightarrow {\mathbb {R}}$ by

$$\begin{aligned} \psi _\xi (s) = \langle \xi , e^{-sA} {\mathcal {K}}_{\le c_1 N^{\gamma _1}} ({\mathcal {N}}_{\ge c_2 N^{\gamma _2}}+1) e^{sA}\xi \rangle , \end{aligned}$$

and we compute

$$\begin{aligned} \begin{aligned} \partial _s \psi _\xi (s)&= 2\mathrm{Re}\,\langle \xi , e^{-sA} \big [ {\mathcal {K}}_{\le c_1 N^{\gamma _1}}, A_1\big ] ({\mathcal {N}}_{\ge c_2 N^{\gamma _2}}+1) e^{sA}\xi \rangle \\&\quad + 2\mathrm{Re}\,\langle \xi , e^{-sA} {\mathcal {K}}_{\le c_1 N^{\gamma _1}} \big [ {\mathcal {N}}_{\ge c_2 N^{\gamma _2}}, A_1 \big ] e^{sA}\xi \rangle . \end{aligned}\end{aligned}$$

First, we proceed as in (4.20) and obtain with (4.7) that

4.21 $$\begin{aligned} \begin{aligned}&\big | \langle \xi , e^{-sA} [ {\mathcal {K}}_{\le c_1 N^{\gamma _1}}, A_1 ]({\mathcal {N}}_{\ge c_2 N^{\gamma _2}}+1) e^{sA}\xi \rangle \big | \\&\quad \le \frac{CN^{\beta }}{\sqrt{N}} \sum _{\begin{array}{c} r\in P_{H}, p \in P_{L}:\\ |p|\le c_1 N^{\gamma _1} \end{array}} |p| |\eta _r| \Vert a_{r+p}({\mathcal {N}}_{\ge c_2 N^{\gamma _2}}+1)^{1/2} a_{-r} e^{sA}\xi \Vert \Vert a_p ({\mathcal {N}}_{\ge c_2 N^{\gamma _2}}+1)^{1/2}e^{sA}\xi \Vert \\&\quad \le \frac{CN^{\beta +\kappa -\alpha /2}}{\sqrt{N}} \Vert ({\mathcal {N}}_{\ge c_2 N^{\gamma _2} }+1)({\mathcal {N}}_{\ge \frac{1}{2} N^\alpha }+1)^{1/2}e^{sD}\xi \Vert \Vert {\mathcal {K}}_{\le c_1 N^{\gamma _1}}^{1/2}({\mathcal {N}}_{\ge c_2 N^{\gamma _2}}+1)^{1/2}e^{sA}\xi \Vert . \end{aligned} \end{aligned}$$

Equation (4.21) and Lemma 4.2 then imply

4.22 $$\begin{aligned} \begin{aligned}&\big | \langle \xi , e^{-sA} [ {\mathcal {K}}_{\le c_1 N^{\gamma _1}}, A_1 ]({\mathcal {N}}_{\ge c_2 N^{\gamma _2}}+1) e^{sA}\xi \rangle \big | \\&\quad \le CN^{2\beta +2\kappa -\alpha -1} \langle \xi , ({\mathcal {N}}_{\ge c_2 N^{\gamma _2} }+1)^2({\mathcal {N}}_{\ge \frac{1}{2} N^\alpha }+1)\xi \rangle + C\psi _\xi (s). \end{aligned} \end{aligned}$$

Next, we recall the identity in (4.10) and that

$$\begin{aligned} \big [ {\mathcal {K}}_{\le c_1 N^{\gamma _1}}, b^*_{p+r} \big ] = \big [ {\mathcal {K}}_{\le c_1 N^{\gamma _1}}, a^*_{-r} \big ]=0 \end{aligned}$$

whenever $$ r\in P_H, p \in P_L$$ and $$N\in {\mathbb {N}}$$, by assumption on $$ c_1$$ and $$\gamma _1$$. We then estimate

4.23 $$\begin{aligned} \begin{aligned}&\big | \langle \xi , e^{-sA} {\mathcal {K}}_{\le c_1 N^{\gamma _1}}\big [ {\mathcal {N}}_{\ge c_2 N^{\gamma _2}}, A_1\big ] e^{sA}\xi \rangle \big | \\&\quad \le \frac{C}{\sqrt{N}} \sum _{\begin{array}{c} r\in P_{H}, p \in P_{L},\\ v\in \Lambda _+^*: |v|\le c_1N^{\gamma _1} \end{array}} |v|^2 |\eta _r| \Vert a_{r+p}({\mathcal {N}}_{\ge c_2 N^{\gamma _2} }+1)^{-1/2} a_{-r} a_v e^{sD}\xi \Vert \\&\qquad \times \Vert a_p ({\mathcal {N}}_{\ge c_2 N^{\gamma _2} }+1)^{1/2}a_v e^{sD}\xi \Vert \\&\quad \le C N^{\kappa - \alpha /2} \langle e^{sA}\xi , {\mathcal {K}}_{\le c_1 N^{\gamma _1}} ({\mathcal {N}}_{\ge c_2 N^{\gamma _2}}+1) e^{sA}\xi \rangle \le C \psi _\xi (s). \end{aligned} \end{aligned}$$

Hence, putting (4.22) and (4.23) together, we have proved that

$$\begin{aligned} \partial _s \psi _\xi (s) \le CN^{2\beta +2\kappa -\alpha -1} \langle \xi , ({\mathcal {N}}_{\ge c_2 N^{\gamma _2} }+1)^2({\mathcal {N}}_{\ge \frac{1}{2} N^\alpha }+1)\xi \rangle +C \psi _\xi (s). \end{aligned}$$

This implies the second bound in (4.18), by Gronwall’s lemma. $\square$

Next, we seek a bound for the growth of the potential energy operator. To this end, we first compute the commutator of ${\mathcal {V}}_N$ with the antisymmetric operator *A*. We introduce here the shorthand notation for the low-momentum part of the kinetic energy

4.24 $$\begin{aligned} {\mathcal {K}}_L= \sum _{p\in \Lambda _+^*: |p|\le N^{\beta } } p^2 a^*_pa_p = \sum _{p\in P_L } p^2 a^*_pa_p. \end{aligned}$$

### Proposition 4.4

Assume the exponents $\alpha ,\beta$ satisfy (4.4). There exists a constant $C>0$ such that

4.25 $$\begin{aligned} \begin{aligned} {[}{\mathcal {V}}_N, A]&= \frac{1}{\sqrt{N}} \sum _{\begin{array}{c} u\in \Lambda _+^*, p\in P_L:\\ p+u\ne 0 \end{array}} N^{\kappa } ({\widehat{V}}(./N^{1-\kappa })*\eta /N)(u) \big [ b^*_{p+u} a^*_{-u}a_p +h.c. \big ] + {\mathcal {E}}_{[{\mathcal {V}}_N,A]} \end{aligned} \end{aligned}$$

where the self-adjoint operator ${\mathcal {E}}_{[{\mathcal {V}}_N,A]}$ satisfies

4.26 $$\begin{aligned} \begin{aligned} \pm {\mathcal {E}}_{[{\mathcal {V}}_N,A]}&\;\le \delta {\mathcal {V}}_N + \delta ^{-1}CN^{\kappa -2\beta -1} {\mathcal {K}}_L ({\mathcal {N}}_{\ge \frac{1}{2} N^{\alpha }}+1)+ \delta ^{-1}C N^{2\alpha +3\kappa -2} {\mathcal {N}}_+\\&\quad + \delta ^{-1}CN^{\kappa -1} ({\mathcal {N}}_{\ge \frac{1}{2} N^{\alpha }}+1)^2 \end{aligned} \end{aligned}$$

for all $\delta >0$ and for all $N\in {\mathbb {N}}$ sufficiently large.

### Proof

From (4.1), we have

$$\begin{aligned}{}[{\mathcal {V}}_N, A] = [{\mathcal {V}}_N, A_1] +\text {h.c.} \end{aligned}$$

Following [4, Prop. 8.1], we find

4.27 $$\begin{aligned} \begin{aligned} {[}{\mathcal {V}}_N, A_1]+\text {h.c.} =&\; \frac{1}{\sqrt{N}} \sum ^*_{u\in \Lambda _+^*, v\in P_L} N^{\kappa } ({\widehat{V}}(./N^{1-\kappa })*\eta /N)(u) b^*_{u+v} a^*_{-u}a_v \\&+ \Theta _{1}+\Theta _{2}+\Theta _{3}+\Theta _{4} +\text {h.c.}, \end{aligned}\end{aligned}$$

where

4.28 $$\begin{aligned} \begin{aligned} \Theta _{1}&= - \frac{1}{N^{3/2}}\sum ^*_{\begin{array}{c} u \in \Lambda ^*, v \in P_L, \\ r \in P_H^c\cup \{0\} \end{array}} N^{\kappa } {\widehat{V}}((u-r)/N^{1-\kappa })\eta _r b^*_{u+v} a_{-u}^* a_v,\\ \Theta _{2}&= \frac{1}{N^{3/2}}\sum ^*_{\begin{array}{c} u\in \Lambda ^*,p\in \Lambda _+^*,\\ r\in P_{H} , v\in P_{L} \end{array}} N^{\kappa } {\widehat{V}} (u/N^{1-\kappa }) \eta _r b_{p+u}^* a_{v+r-u}^*a_{-r}^*a_{p}a_{v} ,\\ \Theta _{3}&= \frac{1}{N^{3/2}}\sum ^*_{\begin{array}{c} u\in \Lambda ^*,p\in \Lambda _+^*,\\ r\in P_{H}, v\in P_{L} \end{array}}N^{\kappa } {\widehat{V}} (u/N^{1-\kappa }) \eta _r b_{v+r}^* a_{p+u}^*a_{-r-u}^*a_{p}a_{v} ,\\ \Theta _{4}&= -\frac{1}{N^{3/2}}\sum ^*_{\begin{array}{c} u\in \Lambda ^*,p\in \Lambda _+^*,\\ r\in P_{H} , v\in P_{L} \end{array}}N^{\kappa } {\widehat{V}} (u/N^{1-\kappa }) \eta _r b_{v+r}^* a_{-r}^*a_{p+u}^*a_{p}a_{v+u} . \end{aligned} \end{aligned}$$

Here and in the following, the notation $\sum ^*$ indicates that we only sum over those momenta for which the arguments of the creation and annihilation operators are nonzero. The first term on the r.h.s. of (4.27) appears explicitly in (4.25), so let us estimate next the size of the operators $\Theta _{1}$ to $\Theta _{4}$, defined in (4.28). The bounds can be obtained similarly as in the proof of [4, Prop. 8.1].

Consider first $\Theta _{1}$. For $\xi \in {\mathcal {F}}_+^{\le N}$, we switch to position space and find

4.29 $$\begin{aligned} |\langle \xi , \Theta _{1}\xi \rangle |&\le \; \frac{1}{N^{1/2}} \sum _{ r\in P_H^c} |\eta _r|\bigg ( \int _{\Lambda ^2}dxdy\; N^{2-2\kappa }V(N^{1-\kappa }(x-y)) \Vert \check{b}_x \check{a}_y \xi \Vert ^2 \bigg )^{1/2}\nonumber \\&\quad \times \bigg ( \int _{\Lambda ^2}dxdy\; N^{2-2\kappa }V(N^{1-\kappa }(x-y)) \Big \Vert \sum _{v\in P_L} e^{ivx}a_v \xi \Big \Vert ^2 \bigg )^{1/2} \nonumber \\&\le CN^{\alpha +3\kappa /2-1} \Vert {\mathcal {V}}_N^{1/2}\xi \Vert \bigg ( \int _{\Lambda }dx\; e^{i(v-v')x} \sum _{v, v'\in P_L} \langle \xi , a^*_{v'}a_{v} \xi \rangle \bigg )^{1/2} \nonumber \\&\le CN^{\alpha +3\kappa /2-1}\Vert {\mathcal {V}}_N^{1/2}\xi \Vert \Vert {\mathcal {N}}_{\le N^{\beta }}^{1/2}\xi \Vert . \end{aligned}$$

The term $\Theta _{2}$ on the r.h.s. of (4.28) can be controlled by

$$\begin{aligned} \begin{aligned}&|\langle \xi , \Theta _{2}\xi \rangle |\\&\quad =\; \bigg | \frac{1}{ N^{1/2} } \int _{\Lambda ^2}dxdy \;N^{2-2\kappa }V(N^{1-\kappa }(x-y))\!\!\!\!\!\sum _{r\in P_H, v\in P_{L} }\!\!\! e^{ivy}e^{iry} \eta _r \langle \xi , \check{b}_{x}^*\check{a}_y^*a^*_{-r} \check{a}_x a_v\xi \rangle \bigg | \\&\quad \le \; \frac{ \Vert {\eta }_{H}\Vert }{N^{1/2}} \bigg [ \int _{\Lambda ^2}dxdy\; N^{2-2\kappa }V(N^{1-\kappa }(x-y)) \sum _{ v\in P_{L} }|v|^{-2} \Vert \check{b}_x \check{a}_y \xi \Vert ^2 \bigg )^{1/2}\\&\qquad \; \times \bigg ( \int _{\Lambda ^2}dxdy\; N^{2-2\kappa }V(N^{1-\kappa }(x-y)) \sum _{ v\in P_{L} } |v|^{2} \Vert ({\mathcal {N}}_{\ge \frac{1}{2} N^{\alpha }}+1)^{1/2}\check{a}_x a_v \xi \Vert ^2 \bigg )^{1/2}\\&\quad \le \; CN^{ \beta /2+3\kappa /2 -\alpha /2-1/2}\Vert {\mathcal {V}}_N^{1/2}\xi \Vert \Vert {\mathcal {K}}_L^{1/2}({\mathcal {N}}_{\ge \frac{1}{2} N^{\alpha }}+1)^{1/2}\xi \Vert . \end{aligned} \end{aligned}$$

In the last step, we used (4.7) to estimate

4.30 $$\begin{aligned} \int _\Lambda dx\; \Vert ({\mathcal {N}}_{\ge \frac{1}{2} N^{\alpha }}+1)^{1/2}\check{a}_x \xi \Vert ^2&= \sum _{p\in \Lambda _+^*} \Vert ({\mathcal {N}}_{\ge \frac{1}{2} N^{\alpha }}+1)^{1/2} a_p \xi \Vert ^2 \nonumber \\&\le C \sum _{p\in \Lambda _+^*} \Vert a_p({\mathcal {N}}_{\ge \frac{1}{2} N^{\alpha }}+1)^{1/2} \xi \Vert ^2 \nonumber \\&= C\Vert {\mathcal {N}}_+^{1/2}({\mathcal {N}}_{\ge \frac{1}{2} N^{\alpha }}+1)^{1/2} \xi \Vert ^2 \end{aligned}$$

for any $\xi \in {\mathcal {F}}_+^{\le N}$. The contributions $\Theta _{3}$ and $\Theta _{4}$ can be bounded similarly. We find

$$\begin{aligned} \begin{aligned}&|\langle \xi , \Theta _{3}\xi \rangle |\\&\quad =\; \bigg | \frac{1}{ N^{1/2} } \int _{\Lambda ^2}dxdy \;N^{2-2\kappa }V(N^{1-\kappa }(x-y))\!\!\!\sum _{r \in P_H, v\in P_L } \!\!\! e^{-iry} \eta _r\langle \xi , b_{v+r}^*\check{a}_x^*\check{a}^*_y \check{a}_x a_v \xi \rangle \bigg | \\&\quad \le \; \frac{C\Vert \eta _H\Vert }{N^{1/2}} \bigg ( \int _{\Lambda ^2}dxdy\; N^{2-2\kappa }V(N^{1-\kappa }(x-y)) \sum _{ v\in P_{L} }|v|^{-2} \Vert \check{a}_x \check{a}_y \xi \Vert ^2 \bigg )^{1/2}\\&\qquad \; \times \bigg ( \int _{\Lambda ^2}dxdy\; N^{2-2\kappa }V(N^{1-\kappa }(x-y))\sum _{ v\in P_{L} } |v|^{2} \Vert ({\mathcal {N}}_{\ge \frac{1}{2} N^{\alpha }}+1)^{1/2}\check{a}_x a_v \xi \Vert ^2 \bigg )^{1/2}\\&\quad \le \; CN^{\beta /2 + 3\kappa /2 -\alpha /2-1/2} \Vert {\mathcal {V}}_N^{1/2}\xi \Vert \Vert {\mathcal {K}}_L^{1/2}({\mathcal {N}}_{\ge \frac{1}{2} N^{\alpha }}+1)^{1/2}\xi \Vert \end{aligned} \end{aligned}$$

as well as

$$\begin{aligned} \begin{aligned}&|\langle \xi , \Theta _{4}\xi \rangle |\\&\quad =\; \bigg | \frac{1}{ N^{1/2} } \int _{\Lambda ^2}dxdy \;N^{2-2\kappa }V(N^{1-\kappa }(x-y))\!\!\!\!\sum _{r\in P_H, v\in P_L} \!\!\!\!\eta _r e^{-ivy} \langle \xi , b_{v+r}^*a^*_{-r}\check{a}^*_x \check{a}_x \check{a}_y \xi \rangle \bigg | \\&\quad \le \; \frac{C\Vert \eta _H\Vert }{N^{1/2}} \bigg [ \int _{\Lambda ^2} dx dy \; N^{2-2\kappa }V(N^{1-\kappa }(x-y))\sum _{v\in P_{L}}\Vert \check{a}_x \check{a}_y \xi \Vert ^2 \bigg )^{1/2}\\&\qquad \;\times \bigg [ \int _{\Lambda ^2}dxdy \; N^{2-2\kappa }V(N^{1-\kappa }(x-y)) \sum _{r\in P_H, v\in P_{L}} \Vert \check{a}_x a_{v+r}a_{-r} \xi \Vert ^2 \bigg )^{1/2}\\&\quad \le \; CN^{3\beta /2 + 3\kappa /2 -\alpha /2-1/2} \Vert {\mathcal {V}}_N^{1/2}\xi \Vert \Vert ({\mathcal {N}}_{\ge \frac{1}{2} N^{\alpha }}+1)\xi \Vert . \end{aligned} \end{aligned}$$

Summarizing (using $\alpha > 3\beta + 2 \kappa$) we proved that

4.31 $$\begin{aligned} \begin{aligned} \pm \sum _{i=1}^4(\Theta _{i} +\text {h.c.})&\;\le \delta {\mathcal {V}}_N +\delta ^{-1}C N^{2\alpha +3\kappa -2} {\mathcal {N}}_+ + \delta ^{-1}CN^{\kappa -2\beta -1} {\mathcal {K}}_L ({\mathcal {N}}_{\ge \frac{1}{2} N^{\alpha }}+1)\\&\; + \delta ^{-1}CN^{\kappa -1} ({\mathcal {N}}_{\ge \frac{1}{2} N^{\alpha }}+1)^2 \end{aligned} \end{aligned}$$

for any $\delta >0$. Setting ${\mathcal {E}}_{[{\mathcal {V}}_N,A]} = \sum _{i=1}^4(\Theta _{i} +\text {h.c.})$, this proves the claim. $\square$

From Proposition 4.4, we immediately get a bound for the action of $e^A$ on ${\mathcal {V}}_N$.

### Corollary 4.5

Assume the exponents $\alpha ,\beta$ satisfy (4.4). Then, there exists a constant $C>0$ such that

4.32 $$\begin{aligned} \begin{aligned} e^{-sA} {\mathcal {V}}_N e^{sA}&\le C {\mathcal {V}}_N +C(N^\kappa +N^{2\alpha +3\kappa -2}) ({\mathcal {N}}_++1) \\&\quad +CN^{\kappa -2\beta -1} {\mathcal {K}}_L ({\mathcal {N}}_{\ge \frac{1}{2} N^{\alpha } }+1) + C N^{\kappa -3\beta -2} ({\mathcal {N}}_{\ge \frac{1}{2} N^{\alpha } }+1)^3. \end{aligned} \end{aligned}$$

for all $s\in [-1;1]$ and $N\in {\mathbb {N}}$ large enough.

### Proof

We apply Gronwall’s lemma. Given $\xi \in {\mathcal {F}}_+^{\le N}$, we define $\varphi _\xi (s) = \langle \xi , e^{-sA }{\mathcal {V}}_N e^{sA}\xi \rangle$ and compute its derivative s.t.

$$\begin{aligned} \partial _s \varphi _\xi (s) =\langle \xi , e^{-sA }[{\mathcal {V}}_N,A] e^{sA}\xi \rangle . \end{aligned}$$

Hence, we can apply (4.25) and estimate

$$\begin{aligned} \begin{aligned}&\bigg |\frac{1}{\sqrt{N}} \sum _{\begin{array}{c} u\in \Lambda _+^*, v\in P_L:\\ v+u\ne 0 \end{array}} N^{\kappa } \langle e^{sA }\xi , ({\widehat{V}}(./N^{1-\kappa })*\eta /N)(u)b^*_{v+u} a^*_{-u}a_v e^{sA}\xi \rangle \bigg | \\&\quad \le \frac{N^{\kappa /2}\Vert \check{\eta }\Vert _\infty }{N} \bigg ( \int _{\Lambda ^2}dxdy\; N^{2-2\kappa }V(N^{1-\kappa }(x-y)) \Vert \check{a}_x \check{a}_y e^{sA}\xi \Vert ^2 \bigg )^{1/2}\\&\qquad \times \bigg ( \int _{\Lambda ^2}dxdy\; N^{3-3\kappa }V(N^{1-\kappa }(x-y)) \Big \Vert \sum _{v\in P_L} e^{ivx}a_v e^{sA}\xi \Vert ^2 \bigg )^{1/2}\\&\quad \le CN^{\kappa /2}\Vert {\mathcal {V}}_N^{1/2} e^{sA}\xi \Vert \Vert {\mathcal {N}}_{\le N^\beta } e^{sA}\xi \Vert \le CN^{\kappa } \langle \xi , e^{-sA}{\mathcal {N}}_+ e^{sA}\xi \rangle + C \varphi _\xi (s). \end{aligned} \end{aligned}$$

Here, we used (3.10), which shows that $\Vert \check{\eta }\Vert _\infty \le CN$. Using Lemma 4.2, this simplifies to

4.33 $$\begin{aligned} \begin{aligned}&\bigg |\frac{1}{\sqrt{N}} \sum _{\begin{array}{c} u\in \Lambda _+^*, v\in P_L:\\ v+u\ne 0 \end{array}} N^{\kappa } \langle e^{sD }\xi , ({\widehat{V}}(./N^{1-\kappa })*\eta /N)(u)b^*_{u+v} a^*_{-u}a_v e^{sD}\xi \rangle \bigg | \\&\quad \le C \varphi _\xi (s) + CN^\kappa \langle \xi , ({\mathcal {N}}_++1) \xi \rangle . \end{aligned}\end{aligned}$$

Together with (4.25), the bound (4.26) (choosing $\delta =1$) and an application of Lemma 4.2 as well as of Lemma 4.3, the claim follows from Gronwall’s lemma.

$$\square $$

## Quartic Renormalization

To explain why the bounds for ${\mathcal {J}}_N$ obtained in Prop. 4.1 are not enough to show Theorem 1.1, we introduce, for $r\in \Lambda _+^*$, the operators

5.1 $$\begin{aligned} c^*_r = \frac{1}{\sqrt{N}} \sum _{\begin{array}{c} v\in \Lambda _+^*:v\ne -r,\\ v\in P_L, v+r\in P_L^c \end{array}} a^*_{v+r}a_v,  e^*_r = \frac{1}{2 \sqrt{N}} \sum _{\begin{array}{c} v\in \Lambda _+^*:v\ne -r,\\ v\in P_L, v+r\in P_L \end{array}}a^*_{v+r}a_v. \end{aligned}$$

We denote the adjoints of $$ c^*_r$$ and $$e^*_r$$ by $$c_r$$ and $$e_r$$, respectively. Notice in particular that $$ e^*_r = e_{-r}$$ for all $$r\in \Lambda _+^*$$. A straightforward computation shows that

5.2 $$\begin{aligned} \begin{aligned}&\frac{8\pi {\mathfrak {a}}_0N^{\kappa }}{\sqrt{N}}\sum _{\begin{array}{c} p \in P_H^c,q\in P_L: \\ p+q\ne 0 \end{array}} \big [ b^*_{p+q}a^*_{-p}a_q+ \text {h.c.}\big ]\\&\quad = 8\pi {\mathfrak {a}}_0N^{\kappa } \sum _{p\in P_H^c} \Big [ b^*_{-p}e_{-p} + e^*_{-p}b_{-p} + b^*_{-p}e^*_{p} + e_{p}b_{-p} + b^*_{-p}c^*_{p} + c_p b_{-p} \Big ]. \end{aligned} \end{aligned}$$

Together with (4.5), this suggests to bound the Hamiltonian ${\mathcal {J}}_N$ from below by completing the square in the operators $g_r^* := b^*_r + c^*_r + e^*_r$ and $g_r := b_r + c_r + e_r$, for $r\in P_H^c\subset \Lambda _+^*$. A better look at (4.5) reveals, however, that several terms that are needed to complete the square are still hidden in the energy ${\mathcal {H}}_N$. Since these terms are not small, we need to extract them from ${\mathcal {H}}_N$ by conjugation with a unitary operator $e^{D}$, with

5.3 $$\begin{aligned} \begin{aligned} D&= D_1 -D_1^*,  \text { where } D_1 = \frac{1}{2 N} \sum _{r\in P_{H}, p, q \in P_{L}} \eta _r a^*_{p+r}a^*_{q-r}a_pa_q. \end{aligned} \end{aligned}$$

Since $[D,{\mathcal {N}}_+] = 0$, we have the identity

5.4 $$\begin{aligned} e^{-sD} ({\mathcal {N}}_++1)^k e^{sD} = ({\mathcal {N}}_++1)^k \end{aligned}$$

for all $$k \in {\mathbb {N}}$$.

Using $e^D$, we define the final excitation Hamiltonian

5.5 $$\begin{aligned} {\mathcal {M}}_N = e^{-D} {\mathcal {J}}_N^{\text {eff}} e^D. \end{aligned}$$

The next proposition provides an important lower bound for ${\mathcal {M}}_N$. Its proof is given in Sect. 7.

### Proposition 5.1

Suppose the exponents $\alpha$ (in the definition of the set $P_H$ in (3.14)) and $\beta$ (in the definition of the set $P_L$ in (4.2)) are such that

5.6 $$\begin{aligned} i)  \alpha> 3\beta +2\kappa , ii) 1> \alpha +\beta +2\kappa , iii) 5\beta>\alpha ,  iv) \beta> 3\kappa ,  v) 1/2>\beta , \end{aligned}$$

Set $\gamma = \min (\alpha , 1-\alpha -\kappa )$ ($\gamma > 0$ from (5.6)) and let $m_0\in {\mathbb {R}}$ be s.t. $m_0\beta =\alpha$. Let $V\in L^3({\mathbb {R}}^3)$ be compactly supported, pointwise nonnegative and spherically symmetric. Then, ${\mathcal {M}}_N$, as defined as in (5.5), is bounded from below by

5.7 $$\begin{aligned} {\mathcal {M}}_{N} \ge \;4\pi \mathfrak {a}_0 N^{1+\kappa }+ \frac{1}{4} {\mathcal {K}}+ {\mathcal {E}}_{{\mathcal {M}}_N} \end{aligned}$$

for a self-adjoint operator ${\mathcal {E}}_{{\mathcal {M}}_N}$ satisfying

5.8 $$\begin{aligned} \begin{aligned} e^{A}e^D {\mathcal {E}}_{{\mathcal {M}}_{N}}&e^{-D} e^{-A}\\ \ge \;&- C N^{-\beta } {\mathcal {K}}- C N^{-\beta -\kappa }{\mathcal {V}}_N - C N^{\beta + 2\kappa -1} {\mathcal {K}}{\mathcal {N}}_{\ge N^\beta } \\&- C N^{\alpha +\beta + 2\kappa -1} {\mathcal {K}}{\mathcal {N}}_{\ge N^{\lfloor m_0 \rfloor \beta }} \\&- C \sum _{j=3}^{2\lfloor m_0\rfloor -1}N^{ j\beta /2 + \beta /2+2\kappa -1}{\mathcal {K}}{\mathcal {N}}_{\ge \frac{1}{2} N^{j\beta /2}} - CN^{3\alpha +\kappa } \end{aligned} \end{aligned}$$

for all $N\in {\mathbb {N}}$ sufficiently large.

## Proof of Theorem 1.1

For $\varepsilon >0$ sufficiently small, we define

6.1 $$\begin{aligned} \alpha = 14\kappa +4\varepsilon ,  \beta = 4\kappa + \varepsilon \, . \end{aligned}$$

The choice $\kappa < 1/43$ guarantees, if $\varepsilon > 0$ is small enough, that all conditions in (5.6) (and thus also in (3.24) and (4.4)) are satisfied.

From (3.25) and (3.26), we obtain the upper bound

6.2 $$\begin{aligned} E_N \le 4 \pi \mathfrak {a}_0 N^{1+\kappa } + C N^{16\kappa + 4 \varepsilon } \end{aligned}$$

for the ground state energy of $H_N$. From (3.25) and (3.27), on the other hand, we obtain

$$\begin{aligned} {\mathcal {H}}_N \le 2 ({\mathcal {G}}_N - 4 \pi \mathfrak {a}_0 N^{1+\kappa }) + C N^\kappa {\mathcal {N}}_+ + C N^{16\kappa + 4 \varepsilon } \end{aligned}$$

With (6.2) and setting ${\mathcal {G}}'_N = {\mathcal {G}}_N - E_N$, we deduce that

6.3 $$\begin{aligned} {\mathcal {H}}_N \le 2 {\mathcal {G}}'_N + C N^\kappa {\mathcal {N}}_+ + C N^{16\kappa + 4 \varepsilon } \end{aligned}$$

Next, we prove (1.5). From (3.29) and (6.3) we arrive at

$$\begin{aligned} \begin{aligned} {\mathcal {G}}_N&= {\mathcal {G}}_N^{\text {eff}}+ {\mathcal {E}}_{{\mathcal {G}}_N} \ge {\mathcal {G}}_N^\text {eff} - C N^{-(7\kappa +2\varepsilon )/2} {\mathcal {G}}'_N - C N^{-(5\kappa +2\varepsilon )/2} {\mathcal {N}}_+ - C N^{16\kappa + 4 \varepsilon } \end{aligned} \end{aligned}$$

Writing ${\mathcal {G}}_\text {eff} = e^A {\mathcal {J}}_N e^{-A}$ and recalling that $\kappa < 1/43$ (and that $\varepsilon > 0$ is small enough), Prop. 4.1 and (6.3) imply that

$$\begin{aligned} \begin{aligned} {\mathcal {G}}_N \ge \;&e^A {\mathcal {J}}_N^{\text {eff}} e^{-A} + e^A {\mathcal {E}}_{{\mathcal {J}}_N} e^{-A} - C N^{- (7\kappa + 2 \varepsilon ) /2} {\mathcal {G}}'_N - CN^{-(5\kappa +2\varepsilon )/2} {\mathcal {N}}_+ - C N^{16\kappa + 4 \varepsilon } \\ \ge \;&e^A {\mathcal {J}}_N^{\text {eff}} e^{-A} - C N^{-(5 \kappa + 2\varepsilon )/2} {\mathcal {G}}'_N - C N^{- (3\kappa + 2\varepsilon )/2} {\mathcal {N}}_+ - C N^{16\kappa +4\varepsilon } \end{aligned} \end{aligned}$$

Inserting ${\mathcal {J}}_\text {eff} = e^D {\mathcal {M}}_N e^{-D}$ and applying Prop. 5.1, we obtain

6.4 $$\begin{aligned} \begin{aligned} {\mathcal {G}}_N \ge \;&4 \pi \mathfrak {a}_0 N^{1+\kappa } + \frac{1}{4} e^A e^D {\mathcal {K}}e^{-D} e^{-A} + e^A e^D {\mathcal {E}}_{{\mathcal {M}}_N} e^{-D} e^{-A} \\&- C N^{-(5 \kappa + 2\varepsilon )/2} {\mathcal {G}}'_N - C N^{- (3\kappa + 2\varepsilon )/2} {\mathcal {N}}_+ - C N^{16\kappa +4\varepsilon } \end{aligned} \end{aligned}$$

With ${\mathcal {K}}\ge (2\pi )^2 {\mathcal {N}}_+$ and Lemma 4.2 (with $m=0$ and $k=1$) we have

6.5 $$\begin{aligned} \begin{aligned} e^A e^D {\mathcal {K}}e^{-D} e^{-A}&\ge (2\pi )^2 e^A e^D {\mathcal {N}}_+ e^{-D} e^{-A} = (2\pi )^2 e^A {\mathcal {N}}_+ e^{-A} \ge c {\mathcal {N}}_+ \end{aligned} \end{aligned}$$

for a constant $c > 0$ small enough (but independent of *N*). If *N* is large enough, we conclude (using also the upper bound (6.2)), that

6.6 $$\begin{aligned} {\mathcal {N}}_+ \le C {\mathcal {G}}'_N - C e^A e^D {\mathcal {E}}_{{\mathcal {M}}_N} e^{-D} e^{-A} + C N^{16 \kappa + 4 \varepsilon } \end{aligned}$$

To bound the error term $e^A e^D {\mathcal {E}}_{{\mathcal {M}}_N} e^{-D} e^{-A}$, we need (according to (5.8)) to control observables of the form $N^{-1} {\mathcal {K}}{\mathcal {N}}_{\ge c N^\gamma }$. To this end, we observe, first of all, that, by Cauchy–Schwarz and by (6.3),

6.7 $$\begin{aligned} \begin{aligned} N^{-1}{\mathcal {K}}{\mathcal {N}}_{\ge cN^{\gamma }}\le&\; \delta ^{-1} N^{\kappa -2\gamma }{\mathcal {K}}+ \delta N^{2\gamma -\kappa -2}{\mathcal {K}}{\mathcal {N}}_{\ge cN^{\gamma }}^2\\ \le&\; \delta ^{-1} N^{\kappa -2\gamma } {\mathcal {K}}+ 2 \delta N^{2\gamma -\kappa -2}{\mathcal {N}}_{\ge cN^{\gamma }} {\mathcal {G}}_N' {\mathcal {N}}_{\ge cN^{\gamma }} + C \delta N^{-1} {\mathcal {K}}{\mathcal {N}}_{\ge cN^{\gamma }}. \end{aligned} \end{aligned}$$

Choosing $\delta >0$ sufficiently small, we thus have

6.8 $$\begin{aligned} \begin{aligned}&N^{-1}{\mathcal {K}}{\mathcal {N}}_{\ge cN^{\gamma }} \le C N^{\kappa -2\gamma } {\mathcal {K}}+ C N^{2\gamma -\kappa -2} {\mathcal {N}}_{\ge cN^{\gamma }}{\mathcal {G}}_N' {\mathcal {N}}_{\ge cN^{\gamma }}. \end{aligned} \end{aligned}$$

We write

6.9 $$\begin{aligned} {\mathcal {N}}_{\ge cN^{\gamma }} {\mathcal {G}}_N' {\mathcal {N}}_{\ge cN^{\gamma }} = {\mathcal {N}}_{\ge cN^{\gamma }}^2 {\mathcal {G}}_N' + {\mathcal {N}}_{\ge cN^{\gamma }} [{\mathcal {G}}_N', {\mathcal {N}}_{\ge cN^{\gamma }}] . \end{aligned}$$

Using (6.3) (similarly as we did in (6.7)) and ${\mathcal {N}}_{\ge cN^\gamma } \le N$, ${\mathcal {N}}_{\ge cN^{\gamma }}\le CN^{-2\gamma }{\mathcal {K}}$, we can bound the expectation of the first term on the r.h.s. of the last equation, for an arbitrary $\xi \in {\mathcal {F}}_+^{\le N}$, by

6.10 $$\begin{aligned} \begin{aligned}&|\langle \xi , {\mathcal {N}}_{\ge cN^{\gamma }}^2{\mathcal {G}}_N' \xi \rangle | \\&\quad \le \langle \xi , {\mathcal {N}}_{\ge cN^{\gamma }}^3 \xi \rangle ^{1/2}\langle \xi , {\mathcal {G}}_N' {\mathcal {N}}_{\ge cN^{\gamma }}{\mathcal {G}}_N' \xi \rangle ^{1/2}\\&\quad \le CN^{1/2-\gamma } \langle \xi , {\mathcal {K}}{\mathcal {N}}_{\ge cN^{\gamma }}^2 \xi \rangle ^{1/2} \langle \xi , {\mathcal {G}}^{'2}_N \xi \rangle ^{1/2} \\&\quad \le CN^{1/2-\gamma } \langle \xi , {\mathcal {G}}^{'2}_N \xi \rangle ^{1/2}\langle \xi , {\mathcal {N}}_{\ge cN^{\gamma }} {\mathcal {G}}_N' {\mathcal {N}}_{\ge cN^{\gamma }} \xi \rangle ^{1/2} \\&\qquad + CN^{1+\kappa /2-2\gamma }\langle \xi , {\mathcal {G}}^{'2}_N \xi \rangle ^{1/2} \langle \xi , {\mathcal {K}}{\mathcal {N}}_{\ge cN^{\gamma }} \xi \rangle ^{1/2}\\&\quad \le \delta \langle \xi , {\mathcal {N}}_{\ge cN^{\gamma }} {\mathcal {G}}_N' {\mathcal {N}}_{\ge cN^{\gamma }} \xi \rangle + C \delta ^{-1} N^{1-2\gamma } \langle \xi , {\mathcal {G}}^{'2}_N \xi \rangle \\&\qquad + C \delta N^{1+\kappa -2\gamma } \langle \xi , {\mathcal {K}}{\mathcal {N}}_{\ge cN^{\gamma }} \xi \rangle ^{1/2}. \end{aligned} \end{aligned}$$

On the other hand, to estimate the commutator term in Eq. (6.9), we notice that ${\mathcal {A}}:= ({\mathcal {H}}_N+1)^{-1/2} i [{\mathcal {G}}_N',{\mathcal {N}}_{\ge cN^{\gamma }} ] ({\mathcal {H}}_N+1)^{-1/2}$ is a bounded, self-adjoint operator with $\Vert {\mathcal {A}}\Vert \le CN^{\kappa +\alpha /2-\gamma } + CN^{\kappa +\gamma /2}$, by (3.30). Setting $\mu = \max (\alpha , 3\gamma )$, this implies, with (6.3),

6.11 $$\begin{aligned} \begin{aligned}&| \langle \xi , {\mathcal {N}}_{\ge cN^{\gamma }}[{\mathcal {G}}_N', {\mathcal {N}}_{\ge cN^{\gamma }}]\xi \rangle | \\&\quad \le \delta \langle \xi , {\mathcal {N}}_{\ge cN^{\gamma }}({\mathcal {H}}_N +1){\mathcal {N}}_{\ge cN^{\gamma }}\xi \rangle + C \delta ^{-1} N^{2\kappa -2\gamma +\mu }\langle \xi , ({\mathcal {H}}_N +1) \xi \rangle \\&\quad \le 2\delta \langle \xi , {\mathcal {N}}_{\ge cN^{\gamma }}{\mathcal {G}}_N' {\mathcal {N}}_{\ge cN^{\gamma }}\xi \rangle + C \delta N^{1+\kappa -2\gamma } \langle \xi , {\mathcal {K}}{\mathcal {N}}_{\ge cN^{\gamma }}\xi \rangle \\&\qquad + C \delta ^{-1} N^{3\kappa -2\gamma +\mu } \langle \xi , {\mathcal {N}}_+ \xi \rangle + C \delta ^{-1} N^{3\kappa +\alpha -2\gamma +\mu } \Vert \xi \Vert ^2 \, \end{aligned} \end{aligned}$$

for all $\xi \in {\mathcal {F}}_+^{\le N}$. Plugging (6.10) and (6.11) into (6.9), we find that, for sufficiently small $\delta > 0$,

6.12 $$\begin{aligned} \begin{aligned} {\mathcal {N}}_{\ge cN^{\gamma }} {\mathcal {G}}_N' {\mathcal {N}}_{\ge cN^{\gamma }} \le \;&C \delta N^{1+\kappa -2\gamma } {\mathcal {K}}{\mathcal {N}}_{\ge cN^{\gamma }} + C \delta ^{-1} N^{1- 2\gamma } {\mathcal {G}}^{'2}_N \\&+ C \delta ^{-1} N^{3\kappa -2\gamma +\mu } {\mathcal {N}}_+ + C \delta ^{-1} N^{3\kappa -2\gamma +\mu +\alpha } \end{aligned}\end{aligned}$$

Inserting into (6.8) and choosing $\delta > 0$ small enough, we obtain

6.13 $$\begin{aligned} \begin{aligned}&N^{-1} {\mathcal {K}}{\mathcal {N}}_{\ge cN^{\gamma }} \le CN^{\kappa -2\gamma } {\mathcal {K}}+ C N^{-\kappa -1} {\mathcal {G}}^{'2}_N + C N^{2\kappa +\mu -2} {\mathcal {N}}_+ + C N^{2\kappa + \mu +\alpha -2} \end{aligned} \end{aligned}$$

Applying (6.13) to the r.h.s. of (5.8) we find, using also (6.3), (6.1), and the choice $\kappa < 1/43$,

6.14 $$\begin{aligned} \begin{aligned} e^A e^D {\mathcal {E}}_{{\mathcal {M}}_N} e^{-D} e^{-A}&\ge - C N^{-\varepsilon } {\mathcal {N}}_+ - C N^{-(\kappa +\varepsilon )} {\mathcal {G}}'_N - C N^{13\kappa + 3 \varepsilon -1} {\mathcal {G}}^{'2}_N - C N^{43 \kappa + 12 \varepsilon } \end{aligned} \end{aligned}$$

Inserting the last equation into (6.6) and using (6.2), we conclude that for *N* large enough,

$$\begin{aligned} {\mathcal {N}}_+ \le C {\mathcal {G}}'_N + C N^{13\kappa + 3\varepsilon -1} {\mathcal {G}}^{'2}_N + C N^{43 \kappa + 12\varepsilon } \end{aligned}$$

For $\psi _N \in L^2_s (\Lambda ^N)$ with $\Vert \psi _N \Vert = 1$ and $\langle \psi _N, (H_N - E_N)^2 \psi _N \rangle \le \zeta ^2$, the corresponding excitation vector $\xi _N = e^{B} U_N \psi _N$ is such that $\langle \xi _N, {\mathcal {G}}^{'2}_N \xi _N \rangle \le \zeta ^2$ and thus

$$\begin{aligned} \langle \xi _N, {\mathcal {N}}_+ \xi _N \rangle \le C \left[ \zeta + \zeta ^2 N^{13\kappa +3\varepsilon -1} + N^{43 \kappa + 12\varepsilon } \right] \end{aligned}$$

which proves (1.5), using Lemma 3.2. From (6.3), we obtain also

6.15 $$\begin{aligned} \langle \xi _N, {\mathcal {H}}_N \xi _N \rangle \le C \left[ \zeta N^{\kappa } + \zeta ^2 N^{14\kappa + 3\varepsilon -1} + N^{44 \kappa + 12\varepsilon } \right] , \end{aligned}$$

an estimate that will be needed to arrive at (1.6).

Evaluating (6.14) on a normalized ground state $\xi _N$ of ${\mathcal {G}}_N$ and inserting the result in (6.4) we also deduce that

$$\begin{aligned} E_N \ge 4 \pi \mathfrak {a}_0 N^{1+\kappa } - C N^{43 \kappa + 12\varepsilon } \end{aligned}$$

Together with the upper bound (6.2), this concludes the proof of (1.3).

We still have to show (1.6) for $k > 0$. To this end, we will prove the stronger bound (1.8); Eq. (1.6) follows then immediately from ${\mathcal {N}}_+ \le {\mathcal {H}}_N$ and by Lemma 3.2. We denote by $Q_\zeta$ the spectral subspace of ${\mathcal {G}}_N$ associated with energies below $E_N + \zeta$. We use induction to show that for all $k \in {\mathbb {N}}$, there exists a constant $C > 0$ (depending on *k*) such that

6.16 $$\begin{aligned} \sup _{\xi \in Q_{\zeta }} \frac{\langle \xi , ({\mathcal {H}}_N +1) ({\mathcal {N}}_++1)^{2k} \xi \rangle }{ \Vert \xi \Vert ^2} \le C \left[ N^{44\kappa +12\varepsilon } + \zeta ^2 N^{20\kappa + 5\varepsilon } \right] ^{2k+1} \end{aligned}$$

for all $k \in {\mathbb {N}}$. This proves (1.8) and thus, with the bound ${\mathcal {N}}_+\le {\mathcal {H}}_N$ and with Lemma 3.2, also (1.6). The case $k=0$ follows from (6.15). From now on, we assume (6.16) to hold true, and we prove the same bound, with *k* replaced by $(k+1)$ (and with a new constant *C*). To this end, we start by observing that combining (6.3) and (6.6),

$$\begin{aligned} {\mathcal {H}}_N +1 \le C N^\kappa {\mathcal {G}}'_N - C N^\kappa e^A e^D {\mathcal {E}}_{{\mathcal {M}}_N} e^{-D} e^{-A} + C N^{17 \kappa + 4 \varepsilon } \end{aligned}$$

Hence,

6.17 $$\begin{aligned} \begin{aligned} ({\mathcal {N}}_+ +1)^{2(k+1)} ({\mathcal {H}}_N +1)&= ({\mathcal {N}}_+ +1)^{k+1} ({\mathcal {H}}_N +1) ({\mathcal {N}}_+ +1)^{k+1} \\&\le C N^\kappa ({\mathcal {N}}_+ +1)^{k+1} {\mathcal {G}}'_N ({\mathcal {N}}_+ +1)^{k+1} \\&\quad - C N^\kappa ({\mathcal {N}}_+ +1)^{k+1} e^A e^D {\mathcal {E}}_{{\mathcal {M}}_N} e^{-D} e^{-A} ({\mathcal {N}}_+ +1)^{k+1} \\&\quad + C N^{17\kappa +4\varepsilon } ({\mathcal {N}}_+ +1)^{2(k+1)} \end{aligned} \end{aligned}$$

We estimate the first term on the r.h.s. by

$$\begin{aligned} \begin{aligned}&N^\kappa ({\mathcal {N}}_+ +1)^{k+1} {\mathcal {G}}'_N ({\mathcal {N}}_+ +1)^{k+1} \\&\quad \le N^\kappa ({\mathcal {N}}_+ +1)^{2(k+1)} {\mathcal {G}}'_N + N^\kappa ({\mathcal {N}}_+ +1)^{k+1} [ {\mathcal {G}}'_N, ({\mathcal {N}}_+ +1)^{k+1} ] \\&\quad = N^\kappa ({\mathcal {N}}_+ +1)^{2(k+1)} {\mathcal {G}}'_N \\&\quad \quad + N^\kappa \sum _{j=1}^{k+1} {k+1 \atopwithdelims ()j} ({\mathcal {N}}_+ +1)^{k+1} \text {ad}_{{\mathcal {N}}_+}^{(j)} ({\mathcal {G}}_N) ({\mathcal {N}}_+ +1)^{k+1-j} \end{aligned} \end{aligned}$$

By Cauchy–Schwarz, we find

$$\begin{aligned} \begin{aligned}&N^\kappa ({\mathcal {N}}_+ +1)^{k+1} {\mathcal {G}}'_N ({\mathcal {N}}_+ +1)^{k+1} \\&\quad \le N^\kappa ({\mathcal {N}}_+ +1)^{2(k+1)} + N^\kappa {\mathcal {G}}'_N ({\mathcal {N}}_+ +1)^{2(k+1)} {\mathcal {G}}'_N \\&\qquad + N^\kappa \sum _{j=1}^{k+1} {k+1 \atopwithdelims ()j} ({\mathcal {N}}_+ +1)^{k+1} \text {ad}_{{\mathcal {N}}_+}^{(j)} ({\mathcal {G}}_N) ({\mathcal {N}}_+ +1)^{k+1-j} \end{aligned} \end{aligned}$$

With $({\mathcal {N}}_+ +1)^{2(k+1)} \le ({\mathcal {N}}_+ +1)^{2k+1} ({\mathcal {H}}_N+1)$ and with the estimate

6.18 $$\begin{aligned} \Vert ({\mathcal {H}}_N + 1)^{-1/2} \text {ad}_{{\mathcal {N}}_+}^{(j)} ({\mathcal {G}}_N) ({\mathcal {H}}_N +1)^{-1/2} \Vert \le C N^{7\kappa /3 + 2 \varepsilon /3} \end{aligned}$$

from (3.31) we obtain, using again Cauchy–Schwarz,

$$\begin{aligned} \begin{aligned}&N^\kappa \langle \xi , ({\mathcal {N}}_+ +1)^{k+1} {\mathcal {G}}'_N ({\mathcal {N}}_+ +1)^{k+1} \xi \rangle \\&\quad \le \; C \left[ N^\kappa \zeta ^2 + N^{7\kappa /3 + 2 \varepsilon /3} \right] \Vert \xi \Vert ^2 \\&\qquad \times \left[ \sup _{\xi \in Q_{\zeta }} \frac{\langle \xi , ({\mathcal {N}}_+ +1)^{2(k+1)} ({\mathcal {H}}_N+1) \xi \rangle }{\Vert \xi \Vert ^2} \right] ^{1/2} \\&\qquad \left[ \sup _{\xi \in Q_{\zeta }} \frac{\langle \xi , ({\mathcal {N}}_+ +1)^{2k} ({\mathcal {H}}_N+1) \xi \rangle }{\Vert \xi \Vert ^2} \right] ^{1/2} \end{aligned} \end{aligned}$$

for every $\xi \in Q_{\zeta }$. Hence, for any $\delta > 0$, we have

6.19 $$\begin{aligned} \begin{aligned}&N^\kappa \frac{\langle \xi , ({\mathcal {N}}_+ +1)^{k+1} {\mathcal {G}}'_N ({\mathcal {N}}_+ +1)^{k+1} \xi \rangle }{\Vert \xi \Vert ^2} \\&\quad \le \delta \sup _{\xi \in Q_{\zeta }} \frac{\langle \xi , ({\mathcal {N}}_+ +1)^{2(k+1)} ({\mathcal {H}}_N+1) \xi \rangle }{\Vert \xi \Vert ^2} \\&\qquad + C \delta ^{-1} \left[ N^\kappa \zeta ^2 + N^{7\kappa /3 + 2 \varepsilon /3} \right] ^2 \sup _{\xi \in Q_{\zeta }} \frac{\langle \xi , ({\mathcal {N}}_+ +1)^{2k} ({\mathcal {H}}_N +1) \xi \rangle }{\Vert \xi \Vert ^2} \end{aligned} \end{aligned}$$

To bound the contribution proportional to $e^A e^D {\mathcal {E}}_{{\mathcal {M}}_N} e^{-D} e^{-A}$ on the r.h.s. of (6.17), we have to control, according to (6.8), terms of the form

$$\begin{aligned} \begin{aligned}&({\mathcal {N}}_+ +1)^{k+1} {\mathcal {N}}_{\ge cN^\gamma } {\mathcal {G}}'_N {\mathcal {N}}_{\ge cN^\gamma } ({\mathcal {N}}_+ +1)^{k+1} \\&\quad = (({\mathcal {N}}_++1)^{k+1} {\mathcal {N}}_{\ge cN^\gamma })^2 {\mathcal {G}}'_N + ({\mathcal {N}}_+ +1)^{k+1} {\mathcal {N}}_{\ge cN^\gamma } \left[ {\mathcal {G}}'_N, ({\mathcal {N}}_+ +1)^{k+1} {\mathcal {N}}_{\ge cN^\gamma } \right] \\&\quad =: \text {A} + \text {B} \end{aligned} \end{aligned}$$

For an arbitrary $\xi \in Q_{\zeta }$, we can bound the expectation of $\text {A}$ by Cauchy–Schwarz as

6.20 $$\begin{aligned} \begin{aligned} \frac{\langle \xi , \text {A} \xi \rangle }{\Vert \xi \Vert ^2}&\le \frac{\langle \xi , (({\mathcal {N}}_+ +1)^{k+1} {\mathcal {N}}_{\ge cN^\gamma })^2 \xi \rangle }{\Vert \xi \Vert ^2} + \frac{\langle {\mathcal {G}}'_N \xi , (({\mathcal {N}}_+ +1)^{k+1} {\mathcal {N}}_{\ge cN^\gamma })^2 {\mathcal {G}}'_N \xi \rangle }{\Vert \xi \Vert ^2} \\&\le N^2 (1+ \zeta ^2) \, \sup _{\xi \in Q_{\zeta }} \frac{\langle \xi , ({\mathcal {N}}_+ +1)^{2k} {\mathcal {N}}_{\ge cN^\gamma }^2 \xi \rangle }{\Vert \xi \Vert ^2} \\&\le N^{2-2\gamma } (1+ \zeta ^2) \, \sup _{\xi \in Q_{\zeta }} \frac{\langle \xi , ({\mathcal {N}}_+ +1)^{2k+1} {\mathcal {K}}\xi \rangle }{\Vert \xi \Vert ^2} \\&\le N^{2-2\gamma } (1+ \zeta ^2) \, \left[ \sup _{\xi \in Q_{\zeta }} \frac{\langle \xi , ({\mathcal {N}}_+ +1)^{2k} {\mathcal {K}}\xi \rangle }{\Vert \xi \Vert ^2} \right] ^{1/2} \\&\quad \times \left[ \sup _{\xi \in Q_{\zeta }} \frac{\langle \xi , ({\mathcal {N}}_+ +1)^{2(k+1)} {\mathcal {K}}\xi \rangle }{\Vert \xi \Vert ^2} \right] ^{1/2} \end{aligned} \end{aligned}$$

As for the term $\text {B}$, we can write

$$\begin{aligned} \begin{aligned} \text {B} = \;&({\mathcal {N}}_+ +1)^{k+1} {\mathcal {N}}_{\ge c N^\gamma }^2 \left[ {\mathcal {G}}'_N, ({\mathcal {N}}_+ +1)^{k+1} \right] \\&+ ({\mathcal {N}}_+ +1)^{k+1} {\mathcal {N}}_{\ge c N^\gamma } \left[ {\mathcal {G}}'_N, {\mathcal {N}}_{\ge cN^\gamma } \right] ({\mathcal {N}}_+ +1)^{k+1} \\ = \;&\sum _{j=1}^{k+1} {k+1 \atopwithdelims ()j} ({\mathcal {N}}_+ +1)^{k+1} {\mathcal {N}}_{\ge c N^\gamma }^2 \text {ad}^{(j)}_{{\mathcal {N}}_+} ({\mathcal {G}}'_N) ({\mathcal {N}}_+ +1)^{k+1-j} \\&+ ({\mathcal {N}}_+ +1)^{k+1} {\mathcal {N}}_{\ge c N^\gamma } \left[ {\mathcal {G}}'_N, {\mathcal {N}}_{\ge cN^\gamma } \right] ({\mathcal {N}}_+ +1)^{k+1} \end{aligned} \end{aligned}$$

From (6.18) and using (3.30) to estimate

$$\begin{aligned} \Vert ({\mathcal {H}}_N + 1)^{-1/2} [ {\mathcal {N}}_{\ge c N^\gamma } , {\mathcal {G}}_N ] ({\mathcal {H}}_N + 1)^{-1/2} \Vert \le C N^{8\kappa +2\varepsilon -\gamma }+C N^{\kappa +\gamma /2}, \end{aligned}$$

we obtain for every $\xi \in Q_{\zeta }$ that

$$\begin{aligned} \begin{aligned}&|\langle \xi , \text {B} \xi \rangle | \\&\quad \le \; CN^{7\kappa /3+2\varepsilon /3} \Vert ({\mathcal {H}}_N+1)^{1/2} {\mathcal {N}}_{\ge c N^\gamma }^2 ({\mathcal {N}}_+ +1)^{k+1} \xi \Vert \Vert ({\mathcal {H}}_N + 1)^{1/2} ({\mathcal {N}}_+ +1)^{k} \xi \Vert \\&\qquad + C N^{8\kappa +2\varepsilon -\gamma }\Vert ({\mathcal {H}}_N+1)^{1/2} {\mathcal {N}}_{\ge c N^\gamma } ({\mathcal {N}}_+ +1)^{k+1} \xi \Vert \Vert ({\mathcal {H}}_N + 1)^{1/2} ({\mathcal {N}}_+ +1)^{k+1} \xi \Vert \\&\qquad +C N^{\kappa +\gamma /2}\Vert ({\mathcal {H}}_N+1)^{1/2} {\mathcal {N}}_{\ge c N^\gamma } ({\mathcal {N}}_+ +1)^{k+1} \xi \Vert \Vert ({\mathcal {H}}_N + 1)^{1/2} ({\mathcal {N}}_+ +1)^{k+1} \xi \Vert . \end{aligned} \end{aligned}$$

Applying the bounds ${\mathcal {N}}_+\le N$, ${\mathcal {N}}_{\ge c N^\gamma }\le C N^{-2\gamma }{\mathcal {K}}$ and (6.3) yields on the one hand

$$\begin{aligned}\begin{aligned}&\Vert ({\mathcal {H}}_N+1)^{1/2} {\mathcal {N}}_{\ge c N^\gamma } ({\mathcal {N}}_+ +1)^{k+1} \xi \Vert \Vert ({\mathcal {H}}_N + 1)^{1/2} ({\mathcal {N}}_+ +1)^{k+1} \xi \Vert \\&\quad \le C \Vert {\mathcal {G}}_N'{\mathcal {N}}_{\ge cN^\gamma } ({\mathcal {N}}_++1)^{k+1} \xi \Vert \Vert ({\mathcal {H}}_N + 1)^{1/2} ({\mathcal {N}}_+ +1)^{k+1} \xi \Vert \\&\qquad + CN^{1+\kappa /2-\gamma }\Vert ({\mathcal {H}}_N + 1)^{1/2} ({\mathcal {N}}_+ +1)^{k+1} \xi \Vert ^2\\&\quad \le \delta \langle \xi , ({\mathcal {N}}_++1)^{k+1}{\mathcal {N}}_{\ge cN^\gamma } {\mathcal {G}}_N'{\mathcal {N}}_{\ge cN^\gamma } ({\mathcal {N}}_++1)^{k+1} \xi \rangle \\&\qquad +C(\delta ^{-1}+N^{1+\kappa /2-\gamma })\Vert ({\mathcal {H}}_N + 1)^{1/2} ({\mathcal {N}}_+ +1)^{k+1} \xi \Vert ^2 \end{aligned}\end{aligned}$$

for any $\delta > 0$. Since $8\kappa +2\varepsilon -\gamma \le 1+\kappa /2-\gamma$ and $\kappa +\gamma /2 \le 1+\kappa /2-\gamma$ for all $\gamma \le \alpha$ if $\kappa <1/43$, this implies with the choice $\delta =\frac{1}{4} (N^{8\kappa +2\varepsilon -\gamma } + N^{ \kappa +\gamma /2} )^{-1}$ that

6.21 $$\begin{aligned} \begin{aligned} |\langle \xi , \text {B} \xi \rangle | \le \;&CN^{7\kappa /3+2\varepsilon /3} \Vert ({\mathcal {H}}_N+1)^{1/2} {\mathcal {N}}_{\ge c N^\gamma }^2 ({\mathcal {N}}_+ +1)^{k+1} \xi \Vert \Vert ({\mathcal {H}}_N + 1)^{1/2} ({\mathcal {N}}_+ +1)^{k} \xi \Vert \\&+C(N^{1+17\kappa /2+2\varepsilon -\gamma }+N^{1+3\kappa /2-\gamma /2})\Vert ({\mathcal {H}}_N + 1)^{1/2} ({\mathcal {N}}_+ +1)^{k+1} \xi \Vert ^2\\&+ \frac{1}{4} \langle \xi , ({\mathcal {N}}_++1)^{k+1}{\mathcal {N}}_{\ge cN^\gamma } {\mathcal {G}}_N'{\mathcal {N}}_{\ge cN^\gamma } ({\mathcal {N}}_++1)^{k+1} \xi \rangle . \end{aligned} \end{aligned}$$

On the other hand, we can estimate

6.22 $$\begin{aligned} \begin{aligned}&\Vert ({\mathcal {H}}_N + 1)^{1/2} {\mathcal {N}}_{\ge cN^\gamma }^2 ({\mathcal {N}}_+ +1)^{k+1} \xi \Vert \\&\quad \le N \Vert ({\mathcal {K}}+ 1)^{1/2} {\mathcal {N}}_{\ge cN^\gamma }({\mathcal {N}}_+ +1)^{k+1} \xi \Vert + \Vert {\mathcal {V}}_N^{1/2} {\mathcal {N}}_{\ge cN^\gamma }^2 ({\mathcal {N}}_+ +1)^{k+1} \xi \Vert . \end{aligned} \end{aligned}$$

Expressing ${\mathcal {V}}_N$ in position space, we find, with $\phi = {\mathcal {N}}_{\ge cN^\gamma }({\mathcal {N}}_+ +1)^{k+1} \xi$,

6.23 $$\begin{aligned} \Vert {\mathcal {V}}_N^{1/2} {\mathcal {N}}_{\ge cN^\gamma } \phi \Vert ^2 = \int dx dy \, N^{2-2\kappa } V (N^{1-\kappa } (x-y)) \Vert {\check{a}}_x {\check{a}}_y {\mathcal {N}}_{\ge cN^\gamma } \phi \Vert ^2 \end{aligned}$$

We have

$$\begin{aligned} {\check{a}}_x {\mathcal {N}}_{\ge cN^\gamma } = ({\mathcal {N}}_{\ge cN^\gamma } + 1) \check{a}_x - a (\check{\chi }_x) \end{aligned}$$

where

$$\begin{aligned} \check{\chi }_x (y) = \check{\chi } (y-x) = \sum _{p \in \Lambda ^*_+ : |p| \le c N^\gamma } e^{i p \cdot (x-y)} \, \end{aligned}$$

is such that $\Vert \check{\chi }_x \Vert = \Vert \chi \Vert \le C N^{3\gamma /2}$. Hence, we find

$$\begin{aligned} \begin{aligned} \Vert {\check{a}}_x {\check{a}}_y&{\mathcal {N}}_{\ge cN^\gamma } \phi \Vert \le N \Vert {\check{a}}_x {\check{a}}_y \phi \Vert + N^{1/2} \Vert \check{\chi }_x \Vert \Vert {\check{a}}_y \phi \Vert + N^{1/2} \Vert \check{\chi }_y \Vert \Vert {\check{a}}_x \phi \Vert .\end{aligned} \end{aligned}$$

Inserting in (6.23), we find

$$\begin{aligned} \Vert {\mathcal {V}}_N^{1/2} {\mathcal {N}}_{\ge cN^\gamma } \phi \Vert ^2 \le C N^2 \Vert {\mathcal {V}}_N^{1/2} \phi \Vert ^2 + C N^{3\gamma +\kappa } \Vert {\mathcal {N}}_+^{1/2} \phi \Vert ^2 . \end{aligned}$$

From (6.22), we conclude that

$$\begin{aligned} \Vert ({\mathcal {H}}_N + 1)^{1/2} {\mathcal {N}}_{\ge cN^\gamma }^2 {\mathcal {N}}_+^{k+1} \xi \Vert \le N \Vert ({\mathcal {H}}_N + 1)^{1/2}{\mathcal {N}}_{\ge cN^\gamma } ({\mathcal {N}}_++1)^{k+1} \xi \Vert \end{aligned}$$

for all $\gamma \le \alpha = 14 \kappa + 4\varepsilon$, if $\kappa < 1/43$. Using now similar arguments as before (6.21), we conclude that together with (6.21), we have

$$\begin{aligned} \begin{aligned}&|\langle \xi , \text {B} \xi \rangle | \\&\quad \le \; \frac{1}{2} \langle \xi , ({\mathcal {N}}_++1)^{k+1}{\mathcal {N}}_{\ge cN^\gamma } {\mathcal {G}}_N'{\mathcal {N}}_{\ge cN^\gamma } ({\mathcal {N}}_++1)^{k+1} \xi \rangle \\&\qquad \; +C N^{2+10\kappa /3+2\varepsilon /3-\gamma } \Vert ({\mathcal {H}}_N+1)^{1/2} ({\mathcal {N}}_+ +1)^{k+1} \xi \Vert \Vert ({\mathcal {H}}_N + 1)^{1/2} ({\mathcal {N}}_+ +1)^{k} \xi \Vert \\&\qquad \; +C N^{2+14\kappa /3+4\varepsilon /3} \Vert ({\mathcal {H}}_N + 1)^{1/2} ({\mathcal {N}}_+ +1)^{k} \xi \Vert ^2 \\&\qquad \; + C(N^{1+17\kappa /2+2\varepsilon -2\gamma }+N^{1+3\kappa /2-\gamma /2})\Vert ({\mathcal {H}}_N + 1)^{1/2} ({\mathcal {N}}_+ +1)^{k+1} \xi \Vert ^2\\ \end{aligned}\end{aligned}$$

Combining this with (6.20), we arrive at

$$\begin{aligned} \begin{aligned}&\frac{\langle \xi , ({\mathcal {N}}_+ +1)^{k+1} {\mathcal {N}}_{\ge c N^\gamma } {\mathcal {G}}'_N {\mathcal {N}}_{\ge c N^\gamma } ({\mathcal {N}}_+ +1)^{k+1} \xi \rangle }{\Vert \xi \Vert ^2} \\&\quad \le \left[ N^{2-2\gamma } \zeta ^2 + N^{2+10\kappa /3+2\varepsilon /3-\gamma } \right] \left[ \sup _{\xi \in Q_{\zeta }} \frac{\langle \xi , ({\mathcal {N}}_+ +1)^{2k} ({\mathcal {H}}_N +1) \xi \rangle }{\Vert \xi \Vert ^2} \right] ^{1/2} \\&\qquad \times \left[ \sup _{\xi \in Q_{\zeta }} \frac{\langle \xi , ({\mathcal {N}}_+ +1)^{2(k+1)} ({\mathcal {H}}_N +1) \xi \rangle }{\Vert \xi \Vert ^2} \right] ^{1/2} \\&\qquad + CN^{2+14\kappa /3+4\varepsilon /3}\left[ \sup _{\xi \in Q_{\zeta }} \frac{\langle \xi , ({\mathcal {N}}_+ +1)^{2k} ({\mathcal {H}}_N +1) \xi \rangle }{\Vert \xi \Vert ^2} \right] \\&\qquad + C(N^{1+17\kappa /2+2\varepsilon -2\gamma }+N^{1+3\kappa /2-\gamma /2})\left[ \sup _{\xi \in Q_{\zeta }} \frac{\langle \xi , ({\mathcal {N}}_+ +1)^{2(k+1)} ({\mathcal {H}}_N +1) \xi \rangle }{\Vert \xi \Vert ^2} \right] \end{aligned} \end{aligned}$$

for all $\xi \in Q_z$. With (6.8), we obtain

$$\begin{aligned} \begin{aligned}&\frac{N^{-1} \langle \xi , ({\mathcal {N}}_+ +1)^{k+1} {\mathcal {K}}{\mathcal {N}}_{\ge c N^\gamma } ({\mathcal {N}}_+ +1)^{k+1} \xi \rangle }{\Vert \xi \Vert ^2} \\&\quad \le \; C N^{\kappa -2\gamma } \frac{\langle \xi , ({\mathcal {N}}_+ +1)^{k+1} {\mathcal {K}}({\mathcal {N}}_+ +1)^{k+1} \xi \rangle }{\Vert \xi \Vert ^2} \\&\qquad + C \left[ N^{-\kappa } \zeta ^2 + N^{\gamma +7\kappa /3+2\varepsilon /3} \right] \left[ \sup _{\xi \in Q_{\zeta }} \frac{\langle \xi , ({\mathcal {N}}_+ +1)^{2k} ({\mathcal {H}}_N +1) \xi \rangle }{\Vert \xi \Vert ^2} \right] ^{1/2} \\&\qquad \times \left[ \sup _{\xi \in Q_{\zeta }} \frac{\langle \xi , ({\mathcal {N}}_+ +1)^{2(k+1)} ({\mathcal {H}}_N +1) \xi \rangle }{\Vert \xi \Vert ^2} \right] ^{1/2}\\&\qquad + CN^{2\gamma +11\kappa /3+4\varepsilon /3}\left[ \sup _{\xi \in Q_{\zeta }} \frac{\langle \xi , ({\mathcal {N}}_+ +1)^{2k} ({\mathcal {H}}_N +1) \xi \rangle }{\Vert \xi \Vert ^2} \right] \\&\qquad + C(N^{15\kappa /2+2\varepsilon -1}+N^{\kappa /2+3\gamma /2-1})\left[ \sup _{\xi \in Q_{\zeta }} \frac{\langle \xi , ({\mathcal {N}}_+ +1)^{2(k+1)} ({\mathcal {H}}_N +1) \xi \rangle }{\Vert \xi \Vert ^2} \right] . \end{aligned} \end{aligned}$$

Applying this bound to (5.8) and recalling that $\kappa <1/43$, we conclude that

$$\begin{aligned} \begin{aligned}&\frac{N^\kappa \langle \xi , ({\mathcal {N}}_+ +1)^{k+1} e^A e^D {\mathcal {E}}_{{\mathcal {M}}_N} e^{-D} e^{-A} ({\mathcal {N}}_+ +1)^{k+1} \xi \rangle }{\Vert \xi \Vert ^2} \\&\quad \ge \; - C N^{-\varepsilon } \left[ \sup _{\xi \in Q_{\zeta }}\frac{\langle \xi , ({\mathcal {H}}_N+1) ({\mathcal {N}}_+ +1)^{2(k+1)} \xi \rangle }{\Vert \xi \Vert ^2}\right] \\&\qquad - C \left[ N^{20\kappa + 5\varepsilon } \zeta ^2 + N^{44\kappa + 12 \varepsilon } \right] \left[ \sup _{\xi \in Q_{\zeta }} \frac{\langle \xi , ({\mathcal {N}}_+ +1)^{2k} ({\mathcal {H}}_N+1) \xi \rangle }{\Vert \xi \Vert ^2} \right] ^{1/2} \\&\qquad \times \left[ \sup _{\xi \in Q_{\zeta }} \frac{\langle \xi , ({\mathcal {N}}_+ +1)^{2(k+1)} ({\mathcal {H}}_N +1) \xi \rangle }{\Vert \xi \Vert ^2} \right] ^{1/2}.\end{aligned} \end{aligned}$$

Therefore, for any $\delta > 0$, we find (if *N* is large enough)

$$\begin{aligned} \begin{aligned}&\frac{N^\kappa \langle \xi , ({\mathcal {N}}_+ +1)^{k+1} e^A e^D {\mathcal {E}}_{{\mathcal {M}}_N} e^{-D} e^{-A} ({\mathcal {N}}_+ +1)^{k+1} \xi \rangle }{\Vert \xi \Vert ^2} \\&\quad \ge -\delta \sup _{\xi \in Q_{\zeta }} \frac{\langle \xi , ({\mathcal {H}}_N +1) ({\mathcal {N}}_+ +1)^{2(k+1)} \xi \rangle }{\Vert \xi \Vert ^2} \\&\qquad - C \delta ^{-1} \left[ N^{20\kappa +5\varepsilon } \zeta ^2 + N^{44 \kappa + 12 \varepsilon } \right] ^2 \sup _{\xi \in Q_{\zeta }} \frac{\langle \xi , ({\mathcal {H}}_N +1) ({\mathcal {N}}_+ +1)^{2k} \xi \rangle }{\Vert \xi \Vert ^2}. \end{aligned} \end{aligned}$$

From the last bound, (6.19) and (6.17), we obtain

$$\begin{aligned} \begin{aligned}&\frac{\langle \xi , ({\mathcal {N}}_+ +1)^{2(k+1)} ({\mathcal {H}}_N +1) \xi \rangle }{ \Vert \xi \Vert ^2} \\&\quad \le \; \delta \sup _{\xi \in Q_{\zeta }} \frac{\langle \xi , ({\mathcal {N}}_+ +1)^{2(k+1)} ({\mathcal {H}}_N +1) \xi \rangle }{ \Vert \xi \Vert ^2} \\&\qquad + C \delta ^{-1} \left[ N^{20\kappa +5\varepsilon } \zeta ^2 + N^{44 \kappa + 12 \varepsilon } \right] ^2 \sup _{\xi \in Q_{\zeta }} \frac{\langle \xi ., ({\mathcal {N}}_+ +1)^{2k} ({\mathcal {H}}_N +1) \xi \rangle }{ \Vert \xi \Vert ^2} \end{aligned} \end{aligned}$$

for any $\xi \in Q_{\zeta }$. Taking the supremum over all $\xi \in Q_{\zeta }$, and choosing $\delta > 0$ small enough, we arrive at

$$\begin{aligned} \begin{aligned} \sup _{\xi \in Q_{\zeta }}&\frac{\langle \xi , ({\mathcal {N}}_+ +1)^{2(k+1)} ({\mathcal {H}}_N +1) \xi \rangle }{ \Vert \xi \Vert ^2} \\&\le C \left[ N^{20\kappa + 5 \varepsilon } \zeta ^2 + N^{44 \kappa + 12 \varepsilon } \right] ^2 \sup _{\xi \in Q_{\zeta }} \frac{\langle \xi , ({\mathcal {N}}_+ +1)^{2k} ({\mathcal {H}}_N +1) \xi \rangle }{ \Vert \xi \Vert ^2} \\&\le C \left[ N^{20\kappa + 5 \varepsilon } \zeta ^2 + N^{44 \kappa + 12 \varepsilon } \right] ^{2k+1} \end{aligned} \end{aligned}$$

by the induction assumption. $\square$

## Analysis of ${\mathcal {M}}_N$

This section is devoted to the proof of Proposition 5.1. In Sect. 7.1 we establish bounds on the growth of the number of excitations and of their energy with respect to the action of $e^D$, with the quartic operator $D = D_1 - D_1^*$ with

7.1 $$\begin{aligned} D_1 = \frac{1}{2N} \sum _{r \in P_H, p,q \in P_L} \eta _r a_{p+r}^* a_{q-r}^* a_p a_q \end{aligned}$$

as defined in (5.3). In Sect. 7.2, we compute the different parts of the excitation Hamiltonian ${\mathcal {M}}_N$, introduced in (5.5). Finally, in Sect. 7.3, we conclude the proof of Proposition 5.1.

### Growth of Number and Energy of Excitations

The first lemma of this section controls the growth of the number of excitations with high momentum.

#### Lemma 7.1

Assume the exponents $\alpha , \beta$ satisfy (5.6). Let $k\in {\mathbb {N}}_0$, $m=1,2,3$, $0< \gamma \le \alpha$ and $c>0$ ($c < 1$ if $\gamma = \alpha$). Then, there exists a constant $C>0$ such that

7.2 $$\begin{aligned} \begin{aligned} e^{-sD} ({\mathcal {N}}_+ + 1)^{k }({\mathcal {N}}_{\ge c N^{\gamma }}+1)^m e^{sD} \le&\; C({\mathcal {N}}_+ + 1)^{k } ({\mathcal {N}}_{\ge cN^{\gamma }}+1)^m , \end{aligned} \end{aligned}$$

for all $s\in [-1;1]$ and all $N\in {\mathbb {N}}$ large enough.

#### Proof

Since $[{\mathcal {N}}_+ , {\mathcal {N}}_{\ge cN^\gamma } ] = 0$ and $[ {\mathcal {N}}_+ , D] =0$, it is enough to prove the lemma for $k=0$. We consider first $m=1$. For $\xi \in {\mathcal {F}}_+^{\le N}$, we define the function $\varphi _\xi :{\mathbb {R}}\rightarrow {\mathbb {R}}$ by

$$\begin{aligned} \varphi _\xi (s) = \langle \xi , e^{-sD} ({\mathcal {N}}_{\ge c N^{\gamma }}+1) e^{sD} \xi \rangle \end{aligned}$$

so that differentiating yields

7.3 $$\begin{aligned} \begin{aligned} \partial _s \varphi _\xi (s)&=2 \mathrm{Re}\,\langle e^{sD} \xi , \big [{\mathcal {N}}_{\ge c N^{\gamma }}, D_1 \big ] e^{sD} \xi \rangle \end{aligned} \end{aligned}$$

with $D_1$ as in (7.1). By assumption, $N^\alpha \ge N^{\alpha }-N^{\beta } \ge cN^{\gamma }$ for sufficiently large $N\in {\mathbb {N}}$. This implies that

$$\begin{aligned} {[}{\mathcal {N}}_{\ge cN^{\gamma } }, a^*_{p+r}] = a^*_{p+r},  [{\mathcal {N}}_{\ge cN^{\gamma } }, a^*_{q-r}] = a^*_{q-r} \end{aligned}$$

for $r\in P_H$ and $p, q\in P_L$, by (2.1) and (2.10). We then compute

7.4 $$\begin{aligned} \begin{aligned} \big [{\mathcal {N}}_{\ge c N^{\gamma }}, D_1 \big ]&= \frac{1}{ N} \sum _{\begin{array}{c} r\in P_{H}, p, q \in P_{L} \end{array}} \eta _r a^*_{p+r}a^*_{q-r}a_pa_q - \frac{1}{ N} \sum _{\begin{array}{c} r\in P_{H}, p, q \in P_{L}, \\ |p|\ge cN^{\gamma } \end{array}} \eta _r a^*_{p+r}a^*_{q-r}a_pa_q. \end{aligned} \end{aligned}$$

and apply Cauchy–Schwarz to obtain

7.5 $$\begin{aligned} \begin{aligned} | \partial _s \varphi _\xi (s)|&\le \frac{C}{N} \bigg ( \sum _{\begin{array}{c} r\in P_{H}, p, q \in P_{L}, \\ |p+r|\ge cN^{\gamma }, |q-r|\ge cN^{\gamma } \end{array}} \Vert a_{p+r} ({\mathcal {N}}_{\ge cN^{\gamma } }+1)^{-1/2} a_{q-r} e^{sD}\xi \Vert ^2 \bigg )^{1/2}\\&\quad \times \Vert \eta _H\Vert \bigg ( \sum _{\begin{array}{c} p,q \in P_{L} \end{array} } \Vert a_p({\mathcal {N}}_{\ge cN^{\gamma } }+1)^{1/2} a_q e^{sD}\xi \Vert ^2\bigg )^{1/2} \\&\le CN^{\kappa +3\beta /2-\alpha /2} \varphi _\xi (s)\le C\varphi _\xi (s). \end{aligned} \end{aligned}$$

Since the bound is independent of $\xi \in {\mathcal {F}}_+^{\le N}$ and it also holds true if we replace *D* by $-D$ in the definition of $\varphi _\xi$, this proves (7.2), for $m=1$.

For $m=3$, we define

$$\begin{aligned}\psi _\xi (s) = \langle \xi , e^{-sD} ({\mathcal {N}}_{\ge c N^{\gamma }}+1)^3 e^{sD} \xi \rangle \end{aligned}$$

with derivative

$$\begin{aligned} \partial _s \psi _\xi (s) = 2\mathrm{Re}\,\langle e^{sD} \xi , [ ({\mathcal {N}}_{\ge c N^{\gamma }} + 1)^3, D_1 ] e^{sD} \xi \rangle \end{aligned}$$

We have

7.6 $$\begin{aligned} \begin{aligned} {[} ({\mathcal {N}}_{\ge c N^{\gamma }}+1)^3 , D_1 ] = \;&3 ( {\mathcal {N}}_{\ge c N^{\gamma }}+1) [ {\mathcal {N}}_{\ge c N^{\gamma }} , D_1 ] ({\mathcal {N}}_{\ge c N^{\gamma }}+1) \\&+ [ {\mathcal {N}}_{\ge c N^{\gamma }} , [ {\mathcal {N}}_{\ge c N^{\gamma }} , [ {\mathcal {N}}_{\ge c N^{\gamma }} , D_1 ] ] ]. \end{aligned} \end{aligned}$$

The contribution of the first term on the r.h.s. of (7.6) can be controlled as in (7.5) (replacing $e^{sD} \xi$ with $({\mathcal {N}}_{\ge c N^{\gamma }}+1) e^{sD} \xi$). With (7.4) and using again that $N^\alpha \ge N^{\alpha }-N^{\beta } \ge cN^{\gamma }$, we obtain that

$$\begin{aligned} \begin{aligned}&{[} {\mathcal {N}}_{\ge c N^{\gamma }} , [ {\mathcal {N}}_{\ge c N^{\gamma }} , [ {\mathcal {N}}_{\ge c N^{\gamma }} , D_1 ] ] ] \\&\quad = \; \frac{4}{ N} \!\!\sum _{\begin{array}{c} r\in P_{H}, p, q \in P_{L} \end{array}} \eta _r a^*_{p+r}a^*_{q-r}a_pa_q - \frac{7}{ N} \!\!\sum _{\begin{array}{c} r\in P_{H}, p, q \in P_{L}, \\ |p|\ge cN^{\gamma } \end{array}} \eta _r a^*_{p+r}a^*_{q-r}a_pa_q \\&\qquad + \frac{3}{ N} \!\!\sum _{\begin{array}{c} r\in P_{H}, p, q \in P_{L}, \\ |p|,|q| \ge cN^{\gamma } \end{array}} \eta _r a^*_{p+r}a^*_{q-r} a_p a_q . \end{aligned} \end{aligned}$$

All these contributions can be controlled like those in (7.4). We conclude that

$$\begin{aligned} | \partial _s \psi _\xi (s)| \le C \psi _\xi (s) \end{aligned}$$

This proves (7.2) with $m=3$. The case $m=2$ follows by operator monotonicity of the function $x \mapsto x^{2/3}$. $\square$

Next, we prove bounds for the growth of the low-momentum part of the kinetic energy, defined as in (4.17).

#### Lemma 7.2

Assume the exponents $\alpha , \beta$ satisfy (5.6). Let $0< \gamma _1, \gamma _2 \le \alpha$, $c_1, c_2 \ge 0$ (and $c_j \le 1$ if $\gamma _j = \alpha$, for $j=1,2$). Then, there exists a constant $C>0$ such that

7.7 $$\begin{aligned} \begin{aligned} e^{-sD} {\mathcal {K}}_{\le c_1 N^{\gamma _1}} e^{sD} \le&\; {\mathcal {K}}_{\le c_1 N^{\gamma _1}} + N^{2\beta -1} ({\mathcal {N}}_{\ge \frac{1}{2} N^{\alpha }} +1)^2,\\ e^{-sD} {\mathcal {K}}_{\le c_1 N^{\gamma _1}} ({\mathcal {N}}_{\ge c_2 N^{\gamma _2}}+1)e^{sD} \le&\; {\mathcal {K}}_{\le c_1 N^{\gamma _1}}({\mathcal {N}}_{\ge c_2 N^{\gamma _2}} +1) \\&+ N^{2\beta -1} ({\mathcal {N}}_{\ge c_2 N^{\gamma _2}} +1)^2({\mathcal {N}}_{\ge \frac{1}{2} N^{\alpha }} +1)\\ \end{aligned} \end{aligned}$$

for all $s\in [-1;1]$ and all $N\in {\mathbb {N}}$ sufficiently large.

#### Proof

Fix $\xi \in {\mathcal {F}}_+^{\le N}$ and define $\varphi _\xi :{\mathbb {R}}\rightarrow {\mathbb {R}}$ by $\varphi _\xi (s) = \langle \xi , e^{-sD} {\mathcal {K}}_{\le c_1 N^{\gamma _1}} e^{sD}\xi \rangle$ such that

$$\begin{aligned} \begin{aligned} \partial _s \varphi _\xi (s)&= 2\mathrm{Re}\,\langle \xi , e^{-sD} [ {\mathcal {K}}_{\le c_1 N^{\gamma _1}} , D_1 ] e^{sD}\xi \rangle .\\ \end{aligned}\end{aligned}$$

We notice that

$$\begin{aligned} \big [ {\mathcal {K}}_{\le c_1 N^{\gamma _1}}, a^*_{p+r} \big ]=\big [ {\mathcal {K}}_{\le c_1 N^{\gamma _1}}, a^*_{q-r} \big ]=0 \end{aligned}$$

if $r\in P_H$ and $p,q\in P_L$, because $|r|, |p+r|, |q-r|\ge N^{\alpha }- N^{\beta } > c_1 N^{\gamma _1}$ for $N\in {\mathbb {N}}$ large enough.

Using (2.1), we then compute

7.8 $$\begin{aligned} \begin{aligned} {[} {\mathcal {K}}_{\le c_1 N^{\gamma _1}} , D_1 ]&= - \frac{1}{ N} \sum _{\begin{array}{c} r\in P_{H}, p, q \in P_{L}: |p|\le c_1 N^{\gamma _1} \end{array}} p^2 \eta _r a^*_{p+r}a^*_{q-r}a_pa_q. \end{aligned} \end{aligned}$$

and, using that $|p|\le N^{\beta }$ for $p\in P_L$, we obtain with Cauchy–Schwarz

7.9 $$\begin{aligned} \begin{aligned}&\big | \langle \xi , e^{-sD} [ {\mathcal {K}}_{\le c_1 N^{\gamma _1}}, D_1 ] e^{sD}\xi \rangle \big | \\&\quad \le \frac{CN^{\beta }}{ N} \sum _{\begin{array}{c} r\in P_{H}, p, q \in P_{L}: |p|\le c_1 N^{\gamma _1} \end{array}} |p| |\eta _r| \Vert a_{r+p}a_{q-r} e^{sD}\xi \Vert \Vert a_p a_qe^{sD}\xi \Vert \\&\quad \le CN^{5\beta /2+\kappa -\alpha /2-1/2} \Vert ({\mathcal {N}}_{\ge \frac{1}{2} N^\alpha }+1)e^{sD}\xi \Vert \Vert {\mathcal {K}}_{\le c_1 N^{\gamma _1}}^{1/2}e^{sD}\xi \Vert . \end{aligned} \end{aligned}$$

With Lemma 7.1 choosing $c=\frac{1}{2}$ and $\gamma =\alpha$, this implies for $N\in {\mathbb {N}}$ large enough that

$$\begin{aligned} \begin{aligned} \partial _s\varphi _\xi (s)&\le CN^{5\beta /2+\kappa -\alpha /2-1/2} \Vert ({\mathcal {N}}_{\ge \frac{1}{2} N^\alpha }+1) e^{sD}\xi \Vert \Vert {\mathcal {K}}_{\le c_1 N^{\gamma _1}}^{1/2}e^{sD}\xi \Vert \\&\le CN^{2\beta -1} \langle \xi , ({\mathcal {N}}_{\ge \frac{1}{2} N^\alpha }+1)^2 \xi \rangle + C \varphi _\xi (s). \end{aligned} \end{aligned}$$

This proves the first inequality in (7.7), by Gronwall’s lemma and $\alpha > 3\beta +2\kappa \ge 0$.

Next, let us prove the second inequality in (7.7). We define $\psi _\xi :{\mathbb {R}}\rightarrow {\mathbb {R}}$ by

$$\begin{aligned} \psi _\xi (s) = \langle \xi , e^{-sD} {\mathcal {K}}_{\le c_1 N^{\gamma _1}} ({\mathcal {N}}_{\ge c_2 N^{\gamma _2}}+1) e^{sD}\xi \rangle , \end{aligned}$$

and we compute

$$\begin{aligned} \begin{aligned} \partial _s \psi _\xi (s)&= 2\mathrm{Re}\,\langle \xi , e^{-sD} \big [ {\mathcal {K}}_{\le c_1 N^{\gamma _1}}, D_1\big ] ({\mathcal {N}}_{\ge c_2 N^{\gamma _2}}+1) e^{sD}\xi \rangle \\&\quad + 2\mathrm{Re}\,\langle \xi , e^{-sD} {\mathcal {K}}_{\le c_1 N^{\gamma _1}} \big [ {\mathcal {N}}_{\ge c_2 N^{\gamma _2}}, D_1 \big ] e^{sD}\xi \rangle . \end{aligned}\end{aligned}$$

First, we proceed as in (7.9) and obtain with (4.7) that

$$\begin{aligned} \begin{aligned}&\big | \langle \xi , e^{-sD} [ {\mathcal {K}}_{\le c_1 N^{\gamma _1}}, D_1 ]({\mathcal {N}}_{\ge c_2 N^{\gamma _2}}+1) e^{sD}\xi \rangle \big | \\&\quad \le \frac{CN^{\beta }}{ N} \sum _{\begin{array}{c} r\in P_{H}, p, q \in P_{L}: \\ |p|\le c_1 N^{\gamma _1} \end{array}}|p| |\eta _r| \Vert a_{r+p} a_{q-r} ({\mathcal {N}}_{\ge c_2 N^{\gamma _2}}+1)^{1/2} e^{sD}\xi \Vert \\&\qquad \times \Vert a_q a_p ({\mathcal {N}}_{\ge c_2 N^{\gamma _2}}+1)^{1/2} e^{sD}\xi \Vert \\&\quad \le CN^{5\beta /2+\kappa -\alpha /2-1/2}\Vert ({\mathcal {N}}_{\ge c_2 N^{\gamma _2} }+1)({\mathcal {N}}_{\ge \frac{1}{2} N^\alpha }+1)^{1/2}e^{sD}\xi \Vert \\&\qquad \times \Vert {\mathcal {K}}_{\le c_1 N^{\gamma _1}}^{1/2}({\mathcal {N}}_{\ge c_2 N^{\gamma _2}}+1)^{1/2}e^{sD}\xi \Vert . \end{aligned} \end{aligned}$$

Here, we used in the last step that $[a_{q-r}, {\mathcal {N}}_{\ge c_2N^{\gamma _2}}] = a_{q-r}$ for $r\in P_H$, $q\in P_L$ and that ${\mathcal {N}}_{c_2 N^{\gamma _2}} \ge {\mathcal {N}}_{ N^{\alpha }-N^{\beta }}$ for $N\in {\mathbb {N}}$ large enough. The last bound and Lemma 7.1 imply that

7.10 $$\begin{aligned} \begin{aligned}&\big | \langle \xi , e^{-sD} [ {\mathcal {K}}_{\le c_1 N^{\gamma _1}}, D_1 ]({\mathcal {N}}_{\ge c_2 N^{\gamma _2}}+1) e^{sD}\xi \rangle \big | \\&\quad \le CN^{2\beta -1} \langle \xi , ({\mathcal {N}}_{\ge c_2 N^{\gamma _2} }+1)^2({\mathcal {N}}_{\ge \frac{1}{2} N^\alpha }+1)\xi \rangle + C\psi _\xi (s). \end{aligned} \end{aligned}$$

Next, we recall the identity (7.4) and that

$$\begin{aligned} \big [ {\mathcal {K}}_{\le c_1 N^{\gamma _1}}, a^*_{p+r} \big ]=\big [ {\mathcal {K}}_{\le c_1 N^{\gamma _1}}, a^*_{q-r} \big ]=0 \end{aligned}$$

whenever $r\in P_H, p,q \in P_L$ and $N\in {\mathbb {N}}$ is sufficiently large. We then obtain

7.11 $$\begin{aligned} \begin{aligned}&\big | \langle \xi , e^{-sD} {\mathcal {K}}_{\le c_1 N^{\gamma _1}}\big [ {\mathcal {N}}_{\ge c_2 N^{\gamma _2}}, D_1 \big ] e^{sD}\xi \rangle \big | \\&\quad \le \frac{C}{N} \sum _{\begin{array}{c} r\in P_{H}, p,q \in P_{L},\\ v\in \Lambda _+^*: |v|\le c_1N^{\gamma _1} \end{array}} |v|^2 |\eta _r| \Vert a_{r+p}({\mathcal {N}}_{\ge c_2 N^{\gamma _2} }+1)^{-1/2} a_{q-r} a_v e^{sD}\xi \Vert \\&\qquad \times \Vert a_pa_q ({\mathcal {N}}_{\ge c_2 N^{\gamma _2} }+1)^{1/2}a_v e^{sD}\xi \Vert \\&\quad \le C N^{3\beta /2 +\kappa - \alpha /2} \langle e^{sD}\xi , {\mathcal {K}}_{\le c_1 N^{\gamma _1}} ({\mathcal {N}}_{\ge c_2 N^{\gamma _2}}+1) e^{sD}\xi \rangle \le C \psi _\xi (s). \end{aligned} \end{aligned}$$

Hence, putting (7.10) and (7.11) together, we have proved that

$$\begin{aligned} \partial _s \psi _\xi (s) \le CN^{2\beta -1} \langle \xi , ({\mathcal {N}}_{\ge c_2 N^{\gamma _2} }+1)^2({\mathcal {N}}_{\ge \frac{1}{2} N^\alpha }+1)\xi \rangle +C \psi _\xi (s), \end{aligned}$$

which implies the second bound in (7.7), by Gronwall’s lemma. $\square$

It will also be important to control the potential energy operator, restricted to low momenta. We define

7.12 $$\begin{aligned} {\mathcal {V}}_{N,L} = \frac{1}{2N}\sum _{\begin{array}{c} u\in \Lambda ^*, p,q\in \Lambda _+^*:\\ p+u, q+u, p,q \in P_L \end{array}} N^{\kappa }{\widehat{V}}(u/N^{1-\kappa })a^*_{p+u}a^*_{q}a_pa_{q+u}. \end{aligned}$$

Notice that ${\mathcal {V}}_{N,L} = {\mathcal {V}}_{N,L}^*$ by symmetry of the momentum restrictions. To calculate $e^D {\mathcal {V}}_{N,L} e^{-D}$, we will use the next lemma, which will also be useful in the next subsections.

#### Lemma 7.3

Assume the exponents $\alpha , \beta$ satisfy (5.6). Let $F = (F_p)_{p\in \Lambda _+^*}\in \ell ^\infty (\Lambda _+^*)$ and define

7.13 $$\begin{aligned} Z = \frac{1}{2N}\sum _{\begin{array}{c} u\in \Lambda ^*, p,q\in \Lambda _+^*:\\ p+u, q+u, p,q \in P_L \end{array}} F_u a^*_{p+u}a^*_{q}a_pa_{q+u} \end{aligned}$$

Then, there exists a constant $C>0$ such that

7.14 $$\begin{aligned} \begin{aligned} \pm \big ( e^{-sD} Z e^{sD}-Z\big )&\le C\Vert F\Vert _\infty N^{\beta -1} {\mathcal {K}}_L({\mathcal {N}}_{\ge \frac{1}{2} N^{\alpha } }+1) + C\Vert F\Vert _\infty N^{3\beta -2} ({\mathcal {N}}_{\ge \frac{1}{2} N^{\alpha } }+1)^3 \end{aligned} \end{aligned}$$

for all $s\in [-1;1]$, and for all $N\in {\mathbb {N}}$ sufficiently large.

#### Proof

Given $\xi \in {\mathcal {F}}_+^{\le N}$, we define $\varphi _\xi :{\mathbb {R}}\rightarrow {\mathbb {R}}$ by

$$\begin{aligned} \varphi _\xi (s) = \langle \xi , e^{-sD} \text {Z} e^{sD}\xi \rangle , \end{aligned}$$

which has derivative

$$\begin{aligned} \begin{aligned} \partial _s \varphi _\xi (s)&= 2\mathrm{Re}\,\langle \xi , e^{-sD} [ \text {Z}, D_1] e^{sD}\xi \rangle . \end{aligned} \end{aligned}$$

By assumption, we have $\alpha > 3\beta + 2\kappa$ so that $|r|, |v+r|, |w-r| \ge N^\alpha -N^\beta > N^\beta$ if $r\in P_H$ and $v,w\in P_L$, for sufficiently large $N\in {\mathbb {N}}$. This implies in particular that

$$\begin{aligned} {[}a_{p}a_{q+u}, a^*_{v+r}a^*_{w-r}] = 0 \end{aligned}$$

whenever $q+u, p\in P_L$ and $r\in P_H$, $v,w\in P_L$. As a consequence, we find

7.15 $$\begin{aligned} \begin{aligned} {[}\text {Z}, D_1]&= - \frac{1}{2N^{2}}\sum _{\begin{array}{c} u\in \Lambda ^*, r\in P_H, v,w \in P_L: \\ w-u, v+u \in P_L \end{array}} F_u \eta _r a^*_{v+r}a^*_{w-r} a_{w-u} a_{v+u}\\&\quad - \frac{1}{N^{2}}\sum _{\begin{array}{c} u\in \Lambda ^*, r\in P_H, v, w, p\in P_L:\\ p+u, v+u \in P_L \end{array}}F_u \eta _r a^*_{v+r}a^*_{w-r} a^*_{p+u} a_w a_{v+u} a_p. \end{aligned} \end{aligned}$$

With (4.7) and $N^\alpha -N^\beta > \frac{1}{2} N^\alpha$ for $N\in {\mathbb {N}}$ large enough, we can bound

$$\begin{aligned} \begin{aligned}&\bigg | \frac{1}{N^{2}}\sum _{\begin{array}{c} u\in \Lambda ^*, r\in P_H, v,w \in P_L: \\ w-u, v+u \in P_L \end{array}}F_u \eta _r \langle e^{sD}\xi ,a^*_{v+r}a^*_{w-r} a_{w-u} a_{v+u}e^{sD}\xi \rangle \bigg | \\&\quad \le \frac{C\Vert F\Vert _\infty }{N^{2}}\bigg (\sum _{\begin{array}{c} u\in \Lambda ^*, r\in P_H, v,w \in P_L: \\ w-u, v+u \in P_L \end{array}} |v+u|^{-2}\Vert a_{v+r} ({\mathcal {N}}_{\ge \frac{1}{2} N^{\alpha } }+1)^{-1/2}a_{w-r} e^{sD}\xi \Vert ^2 \bigg )^{1/2}\\&\qquad \times \bigg (\sum _{\begin{array}{c} u\in \Lambda ^*, r\in P_H, v,w \in P_L: \\ w-u, v+u \in P_L \end{array}}\eta _r^2 |v+u|^{2} \Vert a_{w-u} ({\mathcal {N}}_{\ge \frac{1}{2} N^{\alpha } }+1)^{1/2}a_{v+u}e^{sD}\xi \Vert ^2 \bigg )^{1/2}\\&\quad \le C\Vert F\Vert _\infty N^{7\beta /2 + \kappa -\alpha /2-3/2} \Vert ({\mathcal {N}}_{\ge \frac{1}{2} N^{\alpha } }+1)^{1/2} e^{sD}\xi \Vert \Vert {\mathcal {K}}_L^{1/2}({\mathcal {N}}_{\ge \frac{1}{2} N^{\alpha } }+1)^{1/2} e^{sD}\xi \Vert . \end{aligned}\end{aligned}$$

and

$$\begin{aligned} \begin{aligned}&\bigg | \frac{1}{N^{2}}\sum _{\begin{array}{c} u\in \Lambda ^*, r\in P_H, v, w, p\in P_L:\\ p+u, v+u \in P_L \end{array}} F_u \eta _r \langle e^{sD}\xi , a^*_{v+r}a^*_{w-r} a^*_{p+u} a_w a_{v+u} a_pe^{sD}\xi \rangle \bigg | \\&\quad \le \frac{C\Vert F\Vert _\infty }{N^{2}}\bigg (\sum _{\begin{array}{c} u\in \Lambda ^*, r\in P_H, v, w, p\in P_L:\\ p+u, v+u \in P_L \end{array}} |p+u|^2|p|^{-2} \\&\qquad \times \Vert a_{v+r}({\mathcal {N}}_{\ge \frac{1}{2} N^{\alpha } }+1)^{-1/2}a_{w-r} a_{p+u} e^{sD}\xi \Vert ^2 \bigg )^{1/2}\\&\qquad \times \bigg (\sum _{\begin{array}{c} u\in \Lambda ^*, r\in P_H, v, w, p\in P_L:\\ p+u, v+u \in P_L \end{array}}\eta _r^2 |p|^{2}|p+u|^{-2}\\&\qquad \times \Vert a_w ({\mathcal {N}}_{\ge \frac{1}{2} N^{\alpha } }+1)^{1/2}a_{v+u}a_pe^{sD}\xi \Vert ^2 \bigg )^{1/2}\\&\quad \le C\Vert F\Vert _\infty N^{5\beta /2 + \kappa -\alpha /2-1} \langle \xi , e^{-sD} {\mathcal {K}}_L({\mathcal {N}}_{\ge \frac{1}{2} N^{\alpha } }+1)e^{sD}\xi \rangle . \end{aligned}\end{aligned}$$

Lemmas 7.1, 7.2 and the assumption $\alpha > 3\beta + 2\kappa \ge 0$ implies

$$\begin{aligned} \begin{aligned} \pm \partial _s \varphi _s(\xi )&\le C\Vert F\Vert _\infty N^{\beta -1} \langle \xi , {\mathcal {K}}_L({\mathcal {N}}_{\ge \frac{1}{2} N^{\alpha } }+1)\xi \rangle \\&\quad + C\Vert F\Vert _\infty N^{3\beta -2}\langle \xi , ({\mathcal {N}}_{\ge \frac{1}{2} N^{\alpha } }+1)^3\xi \rangle . \end{aligned}\end{aligned}$$

Hence, integrating the last equation from zero to $s\in [-1; 1]$ proves the lemma.

$$\square $$

With $\sup _{p\in \Lambda ^*}| N^{\kappa }{\widehat{V}}(p/N^{1-\kappa })|\le CN^\kappa$, we obtain immediately the following result.

#### Corollary 7.4

Assume the exponents $\alpha , \beta$ satisfy (5.6). Then, there exists a constant $C>0$ such that

$$\begin{aligned} \begin{aligned} \pm \big ( e^{-sD} {\mathcal {V}}_{N,L} e^{sD} -{\mathcal {V}}_{N,L} \big )&\le CN^{\beta + \kappa -1} {\mathcal {K}}_L({\mathcal {N}}_{\ge \frac{1}{2} N^{\alpha } }+1) \\&\quad + CN^{3\beta + \kappa -2} ({\mathcal {N}}_{\ge \frac{1}{2} N^{\alpha } }+1)^3 \end{aligned} \end{aligned}$$

for all $s\in [-1;1]$, and for all $N\in {\mathbb {N}}$ sufficiently large.

We also need rough bounds for the conjugation of the full potential energy operator ${\mathcal {V}}_N$. To this end, we will make use of the following estimate for the commutator of ${\mathcal {V}}_N$ with $D = D_1 - D_1^*$, with $D_1$ defined in (7.1).

#### Proposition 7.5

Assume the exponents $\alpha , \beta$ satisfy (5.6). Then,

7.16 $$\begin{aligned} \begin{aligned} {}[{\mathcal {V}}_N, D]&= \frac{1}{2N} \sum _{\begin{array}{c} u\in \Lambda _+^*, p,q\in P_L:\\ p+u, q-u\ne 0 \end{array}} N^{\kappa } ({\widehat{V}}(./N^{1-\kappa })*\eta /N)(u) \big ( a^*_{p+u} a^*_{q-u}a_pa_q +h.c. \big ) \\&\quad + {\mathcal {E}}_{[{\mathcal {V}}_N,D]} \end{aligned} \end{aligned}$$

and there exists a constant $C>0$ such that

7.17 $$\begin{aligned} \begin{aligned} \pm {\mathcal {E}}_{[{\mathcal {V}}_N,D]}&\;\le \delta {\mathcal {V}}_N + CN^{\alpha +\kappa -1}{\mathcal {V}}_N+ CN^{\alpha +\kappa -1}{\mathcal {V}}_{N,L} \\&\quad \; + \delta ^{-1}C N^{\beta +\kappa -1} {\mathcal {K}}_L ({\mathcal {N}}_{\ge \frac{1}{2} N^{\alpha } }+1) + \delta ^{-1}C N^{3\beta +\kappa -1} ({\mathcal {N}}_{\ge \frac{1}{2} N^{\alpha } }+1)^2 \end{aligned} \end{aligned}$$

for all $\delta >0$ and for all $N\in {\mathbb {N}}$ sufficiently large.

#### Proof

We have

$$\begin{aligned} {[}{\mathcal {V}}_N, D] = [{\mathcal {V}}_N, D_1] +\text {h.c.} \end{aligned}$$

To compute the commutator $[ {\mathcal {V}}_N, D_1 ]$, we compute first of all that

$$\begin{aligned}\begin{aligned}&[a^*_{p+u}a^*_q a_p a_{q+u}, a^*_{v+r} a^*_{w-r} a_v a_w] \\&\quad = a^*_{p+u}a^*_q a_{q+u} a^*_{w-r} a_v a_w\delta _{p, v+r} + a^*_{p+u}a^*_q a_p a^*_{w-r} a_v a_w \delta _{q+u, v+r}\\&\qquad + a^*_{p+u}a^*_q a^*_{v+r}a_{q+u} a_v a_w\delta _{p, w-r} + a^*_{p+u}a^*_q a^*_{v+r} a_p a_v a_w \delta _{q+u, w-r}\\&\qquad - a^*_{v+r} a^*_{w-r}a^*_q a_w a_{p} a_{q+u}\delta _{p+u, v} - a^*_{v+r} a^*_{w-r}a^*_{p+u} a_w a_{p} a_{q+u} \delta _{q, v}\\&\qquad - a^*_{v+r} a^*_{w-r} a_v a^*_q a_{p} a_{q+u}\delta _{p+u, w} - a^*_{v+r} a^*_{w-r} a_v a^*_{p+u} a_{p} a_{q+u} \delta _{q, w}. \end{aligned}\end{aligned}$$

Putting the terms in the first and last line on the r.h.s. into normal order, we obtain

7.18 $$\begin{aligned} \begin{aligned} {[}{\mathcal {V}}_N, D_1]+\text {h.c.} =&\; \frac{1}{2N} \sum ^*_{u\in \Lambda ^*, v, w \in P_L} N^{\kappa } ({\widehat{V}}(./N^{1-\kappa })*\eta /N)(u) a^*_{v+u} a^*_{w-u}a_va_w \\&+\Phi _{1}+\Phi _{2}+\Phi _{3}+\Phi _{4} +\text {h.c.}, \end{aligned}\end{aligned}$$

where

7.19 $$\begin{aligned} \begin{aligned} \Phi _{1}&= - \frac{1}{2N^{2}}\sum ^*_{\begin{array}{c} u \in \Lambda ^*, v,w \in P_L, \\ r \in P_H^c\cup \{0\} \end{array}} N^{\kappa } {\widehat{V}}((u-r)/N^{1-\kappa })\eta _r a^*_{v+u} a_{w-u}^* a_va_w,\\ \Phi _{2}&= -\frac{1}{2N^{2}}\sum ^*_{\begin{array}{c} u\in \Lambda ^*, r\in P_{H},\\ v,w\in P_{L} \end{array}}N^{\kappa } {\widehat{V}} (u/N^{1-\kappa }) \eta _r a_{v+r}^* a_{w-r}^* a_{w-u}a_{v+u} ,\\ \Phi _{3}&= \frac{1}{N^{2}}\sum ^*_{\begin{array}{c} u\in \Lambda ^*,q\in \Lambda _+^*,\\ r\in P_{H}, v,w\in P_{L} \end{array}}N^{\kappa } {\widehat{V}} (u/N^{1-\kappa }) \eta _r a_{w-r+u}^* a_{v+r}^*a_{q}^*a_{q+u}a_{v}a_{w} ,\\ \Phi _{4}&= - \frac{1}{N^{2}}\sum ^*_{\begin{array}{c} u\in \Lambda ^*,q\in \Lambda _+^*,\\ r\in P_{H}, v,w\in P_{L} \end{array}}N^{\kappa } {\widehat{V}} (u/N^{1-\kappa }) \eta _r a_{v+r}^* a_{w-r}^* a_{q}^*a_{w}a_{v-u}a_{q+u}. \end{aligned}\end{aligned}$$

The first term on the r.h.s. in (7.18) appears explicitly in (7.16). Hence, let us estimate the size of the operators $\Phi _{1}$ to $\Phi _{4}$, defined in (7.19).

Starting with $\Phi _1$, we switch to position space and find

7.20 $$\begin{aligned} \begin{aligned} |\langle \xi , \Phi _1\xi \rangle |\le&\; \frac{1}{N} \sum _{ r\in P_H^c\cup \{0\}} |\eta _r|\bigg ( \int _{\Lambda ^2}dxdy\; N^{2-2\kappa }V(N^{1-\kappa }(x-y))\Vert \check{b}_x \check{a}_y \xi \Vert ^2 \bigg )^{1/2}\\&\; \times \bigg ( \int _{\Lambda ^2}dxdy\; N^{2-2\kappa }V(N^{1-\kappa }(x-y)) \Big \Vert \sum _{w,v\in P_L} e^{ivx+iwy}a_v a_w\xi \Big \Vert ^2 \bigg )^{1/2}\\ \le&\; CN^{\alpha +\kappa -1} \Vert {\mathcal {V}}_N^{1/2}\xi \Vert \Vert {\mathcal {V}}_{N,L}^{1/2}\xi \Vert . \end{aligned} \end{aligned}$$

The term $\Phi _2$ on the r.h.s. of (7.19) can be controlled by

$$\begin{aligned} \begin{aligned} |\langle \xi , \Phi _{2}\xi \rangle |&=\; \bigg | \frac{1}{ N } \int _{\Lambda ^2}dxdy \;N^{2-2\kappa }V(N^{1-\kappa }(x-y))\\&\quad \times \;\sum ^*_{\begin{array}{c} r\in P_H, \\ v,w \in P_{L} \end{array} } e^{-iwx}e^{-ivy} \eta _r \langle \xi , a^*_{v+r}a^*_{w-r}\check{a}_x\check{a}_y\xi \rangle \bigg | \\&\le \; \frac{ CN^{3 \beta } \Vert \eta _H\Vert }{N} \bigg ( \int _{\Lambda ^2}dxdy\; N^{2-2\kappa }V(N^{1-\kappa }(x-y)) \Vert \check{a}_x \check{a}_y \xi \Vert ^2 \bigg )^{1/2}\\&\quad \;\times \bigg ( \int _{\Lambda ^2}dxdy\; N^{2-2\kappa }V(N^{1-\kappa }(x-y)) \sum _{r\in P_H, v,w\in P_{L} } \Vert a_{v+r}a_{w-r} \xi \Vert ^2 \bigg )^{1/2}\\&\le \; CN^{9\beta /2 +3\kappa /2 -\alpha /2 -3/2}\Vert {\mathcal {V}}_N^{1/2}\xi \Vert \Vert ({\mathcal {N}}_{\ge \frac{1}{2} N^{\alpha }}+1)\xi \Vert . \end{aligned} \end{aligned}$$

Finally, the contributions $\Phi _3$ and $\Phi _{4}$ can be bounded as follows. We obtain

$$\begin{aligned}\begin{aligned} |\langle \xi , \Phi _{3}\xi \rangle |\le&\; \frac{1}{ N } \int _{\Lambda ^2}dxdy \;N^{2-2\kappa }V(N^{1-\kappa }(x-y))\sum _{\begin{array}{c} r\in P_H, \\ v,w \in P_{L} \end{array}} |\eta _r| |\langle \xi , a_{v+r}^*\check{a}_x^*\check{a}^*_y \check{a}_y a_v a_w \xi \rangle | \\ \le&\; \frac{CN^{3\beta /2}\Vert \eta _H\Vert }{N} \bigg ( \int _{\Lambda ^2}dxdy\; N^{2-2\kappa }V(N^{1-\kappa }(x-y)) \sum _{ v\in P_{L} }|v|^{-2} \Vert \check{a}_x \check{a}_y \xi \Vert ^2 \bigg )^{1/2}\\&\; \times \bigg ( N^{\kappa -1}\int _{\Lambda }dx\; \sum _{ v,w\in P_{L} } |v|^{2} \Vert ({\mathcal {N}}_{\ge \frac{1}{2} N^{\alpha }}+1)^{1/2}\check{a}_x a_wa_v \xi \Vert ^2 \bigg )^{1/2}\\ \le&\; CN^{2\beta + 3\kappa /2 -\alpha /2-1/2} \Vert {\mathcal {V}}_N^{1/2}\xi \Vert \Vert {\mathcal {K}}_L^{1/2}({\mathcal {N}}_{\ge \frac{1}{2} N^{\alpha }}+1)^{1/2}\xi \Vert \end{aligned} \end{aligned}$$

as well as

$$\begin{aligned} |\langle \xi , \Phi _{4}\xi \rangle |\le & {} \frac{1}{ N } \int _{\Lambda ^2}dxdy \;N^{2-2\kappa }V(N^{1-\kappa }(x-y))\\&\quad \times \;\!\!\!\!\sum _{r\in P_H, v,w\in P_L}\!\!\!\! |\eta _r| | \langle \xi , a_{v+r}^*a^*_{w-r}\check{a}^*_y a_w \check{a}_x \check{a}_y \xi \rangle | \\&\le \; \frac{CN^{3\beta /2}\Vert \eta _H\Vert }{N} \bigg [ \int _{\Lambda ^2} dx dy \; N^{2-2\kappa }V(N^{1-\kappa }(x-y))\Vert \check{a}_x \check{a}_y \xi \Vert ^2 \bigg )^{1/2}\\&\quad \times \bigg ( N^{\kappa -1}\int _{\Lambda }dy \; \sum _{\begin{array}{c} r\in P_H, \\ v,w \in P_{L} \end{array}} \Vert \check{a}_y a_{v+r}a_{w-r} ({\mathcal {N}}_++1)^{1/2} \xi \Vert ^2 \bigg )^{1/2}\\&\le CN^{ 3\beta +3\kappa /2 -\alpha /2-1/2} \Vert {\mathcal {V}}_N^{1/2}\xi \Vert \Vert ({\mathcal {N}}_{\ge \frac{1}{2} N^{\alpha }}+1)\xi \Vert . \end{aligned}$$

In conclusion, the previous bounds imply with the assumption (5.6) (in particular, since $\alpha > 3\beta +2\kappa$ and $3\beta -2 < 0$) that

7.21 $$\begin{aligned} \begin{aligned}&\pm ( \Phi _{1} + \Phi _{2}+\Phi _{3}+\Phi _{4}+\text {h.c.} )\\&\quad \;\le \delta {\mathcal {V}}_N + CN^{\alpha +\kappa -1}{\mathcal {V}}_N+ CN^{\alpha +\kappa -1}{\mathcal {V}}_{N,L} + \delta ^{-1}C N^{\beta +\kappa -1} {\mathcal {K}}_L ({\mathcal {N}}_{\ge \frac{1}{2} N^{\alpha } }+1) \\&\qquad \; + \delta ^{-1}C N^{3\beta +\kappa -1} ({\mathcal {N}}_{\ge \frac{1}{2} N^{\alpha } }+1)^2 \end{aligned} \end{aligned}$$

holds true in ${\mathcal {F}}_+^{\le N}$ for any $\delta >0$. This concludes the proof. $\square$

With Proposition 7.5, we obtain a bound for the growth of ${\mathcal {V}}_N$.

#### Corollary 7.6

Assume the exponents $\alpha , \beta$ satisfy (5.6). Then, there exists a constant $C>0$ such that the operator inequality

$$\begin{aligned} \begin{aligned} e^{-sD} {\mathcal {V}}_N e^{sD}&\le C {\mathcal {V}}_N + C {\mathcal {V}}_{N,L} + CN^{\beta + \kappa -1} {\mathcal {K}}_L({\mathcal {N}}_{\ge \frac{1}{2} N^{\alpha } }+1) \\&\quad + CN^{3\beta + \kappa }({\mathcal {N}}_{\ge \frac{1}{2} N^{\alpha } }+1). \end{aligned}\end{aligned}$$

for all $s\in [-1;1]$ and for all $N\in {\mathbb {N}}$ sufficiently large.

#### Proof

We apply Gronwall’s lemma. Given a normalized vector $\xi \in {\mathcal {F}}_+^{\le N}$, we define $\varphi _\xi (s) = \langle \xi , e^{-sD }{\mathcal {V}}_N e^{sD}\xi \rangle$ and compute its derivative s.t.

$$\begin{aligned} \partial _s \varphi _\xi (s) =\langle \xi , e^{-sD }[{\mathcal {V}}_N,D] e^{sD}\xi \rangle . \end{aligned}$$

Hence, we can apply (7.16) and estimate

7.22 $$\begin{aligned} \begin{aligned}&\bigg | \frac{1}{2N} \sum _{\begin{array}{c} u\in \Lambda _+^*, v,w\in P_L:\\ v+u, w-u\ne 0 \end{array}} N^{\kappa } ({\widehat{V}}(./N^{1-\kappa })*\eta /N)(u) \langle e^{sD }\xi , a^*_{v+u} a^*_{w-u}a_va_w e^{sD}\xi \rangle \bigg |\\&\quad \le \frac{\Vert \check{\eta }\Vert _\infty }{N} \bigg ( \int _{\Lambda ^2}dxdy\; N^{2-2\kappa }V(N^{1-\kappa }(x-y)) \Vert \check{a}_x \check{a}_y e^{sD}\xi \Vert ^2 \bigg )^{1/2}\\&\qquad \times \bigg ( \int _{\Lambda ^2}dxdy\; N^{2-2\kappa }V(N^{1-\kappa }(x-y)) \Big \Vert \sum _{v,w\in P_L}e^{ivx+iwy} a_va_w e^{sD}\xi \Big \Vert ^2 \bigg )^{1/2}\\&\quad \le C \Vert {\mathcal {V}}_N^{1/2} e^{sD}\xi \Vert \Vert {\mathcal {V}}_{N,L}^{1/2}e^{sD}\xi \Vert \le C \varphi _\xi (s) + C\langle \xi , e^{-sD}{\mathcal {V}}_{N,L}e^{sD}\xi \rangle . \end{aligned} \end{aligned}$$

Here, we used (3.10), which shows that $\Vert \check{\eta }\Vert _\infty \le CN$. Using Corollary 7.4 (recalling that $\alpha > 3\beta +2\kappa$ and $2\beta \le 1$) and ${\mathcal {N}}_{\ge \frac{1}{2} N^{\alpha } }\le N$ in ${\mathcal {F}}_+^{\le N}$, this simplifies to

$$\begin{aligned} \begin{aligned}&\bigg | \frac{1}{2N} \sum _{\begin{array}{c} u\in \Lambda _+^*, v,w\in P_L:\\ v+u, w-u\ne 0 \end{array}} N^{\kappa } ({\widehat{V}}(./N^{1-\kappa })*\eta /N)(u) \langle e^{sD }\xi , a^*_{v+u} a^*_{w-u}a_va_w e^{sD}\xi \rangle \bigg |\\&\quad \le C \varphi _\xi (s) + C \langle \xi , {\mathcal {V}}_{N,L}\xi \rangle + CN^{\beta + \kappa -1} \langle \xi , {\mathcal {K}}_L({\mathcal {N}}_{\ge \frac{1}{2} N^{\alpha } }+1)\xi \rangle \\&\qquad + CN^{3\beta + \kappa } \langle \xi , ({\mathcal {N}}_{\ge \frac{1}{2} N^{\alpha } }+1)\xi \rangle . \end{aligned} \end{aligned}$$

Together with (7.16), the bound (7.17) (choosing $\delta =1$) and an application of Lemma 7.1 and of Lemma 7.2, the claim follows now from Gronwall’s lemma.

$$\square $$

Finally, we need control for the growth of the full kinetic energy operator ${\mathcal {K}}$. To this end, we need to estimate its commutator with *D*.

#### Proposition 7.7

Assume the exponents $\alpha , \beta$ satisfy (5.6). Let $m_0 \in {\mathbb {R}}$ be such that $m_0\beta = \alpha$ (from (5.6) it follows that $3< m_0 < 5$). Then,

7.23 $$\begin{aligned} \begin{aligned} {[}{\mathcal {K}}, D]&= - \frac{1}{2N} \sum _{\begin{array}{c} u\in \Lambda ^*, p,q\in P_L:\\ p+u, q-u\ne 0 \end{array}} N^{\kappa } ({\widehat{V}}(./N^{1-\kappa })*{\widehat{f}}_{N})(u) \big ( a^*_{p+u} a^*_{q-u}a_pa_q +h.c. \big ) \\&\quad + {\mathcal {E}}_{[{\mathcal {K}},D]}, \end{aligned} \end{aligned}$$

where the self-adjoint operator ${\mathcal {E}}_{[{\mathcal {K}},D]}$ satisfies

7.24 $$\begin{aligned} \begin{aligned} \pm {\mathcal {E}}_{[{\mathcal {K}},D]} \le&\; CN^{5\beta /4+\kappa }{\mathcal {K}}_{\le 2 N^{3\beta /2}} +\delta {\mathcal {K}}\\&\;+ C \delta ^{-1}\sum _{j=3}^{2\lfloor m_0\rfloor -1}N^{ j\beta /2 + 3\beta /2+2\kappa -1}{\mathcal {K}}_L({\mathcal {N}}_{\ge \frac{1}{2} N^{j\beta /2}}+1) \\&\; +C \delta ^{-1}N^{ \alpha +\beta +2\kappa -1}{\mathcal {K}}_L({\mathcal {N}}_{\ge \frac{1}{2} N^{\lfloor m_0 \rfloor \beta }} +1) +C \\ \end{aligned} \end{aligned}$$

for all $\delta >0$ and for all $N\in {\mathbb {N}}$ sufficiently large.

#### Proof

Using that $[{\mathcal {K}}, D] = [{\mathcal {K}}, D_1] +\text {h.c.}$, a straight forward computation shows that

7.25 $$\begin{aligned} \begin{aligned} {[}{\mathcal {K}}, D_1] + \text {h.c.}&= -\frac{1}{2N} \sum _{\begin{array}{c} r\in \Lambda ^*, v,w\in P_L:\\ v+r,w-r\ne 0 \end{array}} N^{\kappa } ({\widehat{V}}(./N^{1-\kappa })*{\widehat{f}}_{N})(r) a^*_{v+r} a^*_{w-r}a_va_w\\&\quad + \Sigma _{1} +\Sigma _{2} + \Sigma _{3} +\text {h.c.}, \end{aligned} \end{aligned}$$

where

7.26 $$\begin{aligned} \begin{aligned} \Sigma _{1} =&\; \frac{1}{2N} \sum _{\begin{array}{c} r\in P_H^c\cup \{0\}, v,w\in P_L:\\ v+r, w-r\ne 0 \end{array}} N^{\kappa } ({\widehat{V}}(./N^{1-\kappa })*{\widehat{f}}_{N})(r) a^*_{v+r} a^*_{w-r}a_va_w , \\ \Sigma _{2} =&\; \frac{1}{2N} \sum _{\begin{array}{c} r\in P_H, v,w \in P_L:\\ v+r, w-r\ne 0 \end{array}} N^{3-2\kappa }\lambda _\ell ({\widehat{\chi }}_\ell *{\widehat{f}}_N)(r) a^*_{v+r} a^*_{w-r}a_va_w , \\ \Sigma _{3} =&\; \frac{2}{N } \sum _{\begin{array}{c} r\in P_H, v,w \in P_L:\\ v+r, w-r\ne 0 \end{array} } r\cdot v\,\eta _r a^*_{v+r}a^*_{w-r}a_va_w. \end{aligned} \end{aligned}$$

Let us estimate the size of the operators $\Sigma _{1}, \Sigma _{2}$ and $\Sigma _{3}$. Using $\big |({\widehat{V}}(./N^{1-\kappa })*{\widehat{f}}_{N})(r)\big |\le C$, we control the operator $\Sigma _1$ by

7.27 $$\begin{aligned} |\langle \xi , \Sigma _1\xi \rangle |&= \bigg | \frac{1}{2N} \sum _{\begin{array}{c} r\in P_H^c\cup \{0\}, v,w \in P_L:\nonumber \\ v+r,w-r\ne 0 \end{array}} N^{\kappa } ({\widehat{V}}(./N^{1-\kappa })*{\widehat{f}}_{N})(r) \langle \xi , b^*_{v+r} a^*_{w-r}a_va_w \xi \rangle \bigg |\nonumber \\&\le \frac{CN^\kappa }{ N} \sum _{\begin{array}{c} r\in \Lambda ^*, v,w\in P_L: |r|\le N^{3\beta /2}, \nonumber \\ v+r,w-r\ne 0 \end{array}} \Vert a_{w-r} a_{v+r}\xi \Vert \Vert a_va_w \xi \Vert \nonumber \\&\quad + \frac{CN^\kappa }{N} \sum _{j=3}^{2\lfloor m_0\rfloor -1}\nonumber \\&\quad \!\!\!\!\!\!\!\!\sum _{\begin{array}{c} r\in P_H^c\cup \{0\}, v,w\in P_L: \nonumber \\ N^{j\beta /2}\le |r|\le N^{(j+1)\beta /2}, \\ v+r,w-r\ne 0 \end{array}} \Vert a_{w-r} ({\mathcal {N}}_{\ge \frac{1}{2} N^{j\beta /2}}+1)^{-1/2} a_{v+r}\xi \Vert \Vert a_v({\mathcal {N}}_{\ge \frac{1}{2} N^{j\beta /2}}+1)^{1/2}a_w\xi \Vert \nonumber \\&\quad + \frac{CN^\kappa }{ N} \nonumber \\&\quad \sum _{\begin{array}{c} r\in P_H^c\cup \{0\}, v,w\in P_L: \nonumber \\ N^{\lfloor m_0 \rfloor \beta }\le |r|\le N^{\alpha }, \\ v+r,w-r\ne 0 \end{array}} \Vert a_{w-r} ({\mathcal {N}}_{\ge \frac{1}{2} N^{\lfloor m_0 \rfloor \beta }}+1)^{-1/2} a_{v+r}\xi \Vert \Vert a_v({\mathcal {N}}_{\ge \frac{1}{2} N^{\lfloor m_0 \rfloor \beta }}+1)^{1/2}a_w\xi \Vert .\nonumber \\ \end{aligned}$$

By Cauchy–Schwarz, the first term on the r.h.s. of (7.27) can be controlled by

$$\begin{aligned} \frac{CN^\kappa }{ N} \sum _{\begin{array}{c} r\in \Lambda ^*, v, w\in P_L: |r|\le N^{3\beta /2}, \\ v+r,w-r\ne 0 \end{array}} \Vert a_{w-r} a_{v+r}\xi \Vert \Vert a_va_w \xi \Vert \le CN^{5\beta /4+\kappa } \langle \xi , {\mathcal {K}}_{\le 2 N^{3\beta /2} } \xi \rangle . \end{aligned}$$

The second contribution on the r.h.s. of (7.27) can be bounded by

7.28 $$\begin{aligned} \begin{aligned}&\frac{CN^\kappa }{N} \sum _{j=3}^{2\lfloor m_0\rfloor -1} \!\!\!\!\!\!\!\!\sum _{\begin{array}{c} r\in P_H^c\cup \{0\}, v,w\in P_L: \\ N^{j\beta /2}\le |r|\le N^{(j+1)\beta /2}, \\ v+r,w-r\ne 0 \end{array}} \Vert a_{w-r} ({\mathcal {N}}_{\ge \frac{1}{2} N^{j\beta /2}}+1)^{-1/2} a_{v+r}\xi \Vert \Vert a_w({\mathcal {N}}_{\ge \frac{1}{2} N^{j\beta /2}}+1)^{1/2}a_v\xi \Vert \\&\quad \;\le C \sum _{j=3}^{2\lfloor m_0\rfloor -1}N^{ j\beta /4 + 3\beta /4+\kappa -1/2} \Vert {\mathcal {K}}^{1/2}\xi \Vert \Vert {\mathcal {K}}_L^{1/2} ({\mathcal {N}}_{\ge \frac{1}{2} N^{j\beta /2}}+1)^{1/2}\xi \Vert . \end{aligned} \end{aligned}$$

Similarly, we find that

7.29 $$\begin{aligned} \begin{aligned}&\frac{CN^\kappa }{ N} \sum _{\begin{array}{c} r\in P_H^c\cup \{0\}, v,w\in P_L: \\ N^{\lfloor m_0 \rfloor \beta }\le |r|\le N^{\alpha }, \\ v+r,w-r\ne 0 \end{array}} \Vert a_{w-r} ({\mathcal {N}}_{\ge \frac{1}{2} N^{\lfloor m_0 \rfloor \beta }}+1)^{-1/2} a_{v+r}\xi \Vert \Vert a_w({\mathcal {N}}_{\ge \frac{1}{2} N^{\lfloor m_0 \rfloor \beta }}+1)^{1/2}a_v\xi \Vert \\&\quad \;\le C N^{ \alpha /2 +\beta /2+\kappa -1/2} \Vert {\mathcal {K}}^{1/2}\xi \Vert \Vert {\mathcal {K}}_L^{1/2}({\mathcal {N}}_{\ge \frac{1}{2} N^{\lfloor m_0 \rfloor \beta }}+1)^{1/2}\xi \Vert . \end{aligned} \end{aligned}$$

In summary, the previous three bounds imply that

7.30 $$\begin{aligned} \begin{aligned} \pm \Sigma _1\le&\; CN^{5\beta /4+\kappa }{\mathcal {K}}_{\le 2 N^{3\beta /2}} +\delta {\mathcal {K}}+ C \delta ^{-1}N^{ \alpha +\beta +2\kappa -1}{\mathcal {K}}_L({\mathcal {N}}_{\ge \frac{1}{2} N^{\lfloor m_0 \rfloor \beta }} +1) \\&\;+ C \delta ^{-1}\sum _{j=3}^{2\lfloor m_0\rfloor -1}N^{ j\beta /2 + 3\beta /2+2\kappa -1}{\mathcal {K}}_L({\mathcal {N}}_{\ge \frac{1}{2} N^{j\beta /2}}+1) \end{aligned} \end{aligned}$$

for some constant $C>0$ and all $\delta >0$.

Next, let us switch to $\Sigma _2$ and $\Sigma _3$, defined in (7.26). Since $({\widehat{\chi }}_\ell * {\widehat{f}}_N) (r) = {\widehat{\chi }}_\ell (r) + N^{-1} \eta _r$, with

$$\begin{aligned} {\widehat{\chi }}_\ell (r) = \frac{4\pi }{|r|^2} \left( \frac{\sin (\ell |r|)}{|r|} - \ell \cos (\ell |r|) \right) \end{aligned}$$

we find

$$\begin{aligned} |({\widehat{\chi }}_\ell * {\widehat{f}}_N ) (r)| \le C |r|^{-2} \end{aligned}$$

This, together with Lemma 3.1(i), Cauchy–Schwarz and $\alpha >3\beta +2\kappa$, implies that

7.31 $$\begin{aligned} \begin{aligned} |\langle \xi , \Sigma _2\xi \rangle | \le&\; \frac{CN^{\kappa } }{N} \sum _{\begin{array}{c} r\in P_H, v,w\in P_L:\\ v+r,w-r\ne 0 \end{array}} |r|^{-2} \Vert a_{v+r} ({\mathcal {N}}_{\ge \frac{1}{2} N^{\alpha } }+1)^{-1/2}a_{w-r} \xi \Vert \\&\times \Vert a_v ({\mathcal {N}}_{\ge \frac{1}{2} N^{\alpha }}+1)^{1/2}a_w\xi \Vert \\ \le&\;CN^{-\beta -1/2}\Vert ({\mathcal {N}}_{\ge \frac{1}{2} N^{\alpha }}+1)^{1/2}\xi \Vert \Vert {\mathcal {K}}_L^{1/2}({\mathcal {N}}_{\ge \frac{1}{2} N^{\alpha }}+1)^{1/2}\xi \Vert . \end{aligned} \end{aligned}$$

Similarly, we obtain

7.32 $$\begin{aligned} \begin{aligned} |\langle \xi , \Sigma _3\xi \rangle | \le \;&\frac{C}{N } \sum _{r\in P_H, v,w\in P_L } |r| | v| |\eta _r| \Vert a_{v+r} ({\mathcal {N}}_{\ge \frac{1}{2} N^{\alpha } }+1)^{-1/2}a_{w-r} \xi \Vert \\&\times \Vert a_v a_w({\mathcal {N}}_{\ge \frac{1}{2} N^{\alpha }}+1)^{1/2}\xi \Vert \\ \le&\; CN^{-1/2} \Vert {\mathcal {K}}^{1/2}\xi \Vert \Vert {\mathcal {K}}_L^{1/2}({\mathcal {N}}_{\ge \frac{1}{2} N^{\alpha }}+1)^{1/2} \xi \Vert , \end{aligned} \end{aligned}$$

where we used that $|r|/|v+r|\le 2$ for $r\in P_H$, $v\in P_L$ and $N\in {\mathbb {N}}$ large enough. Combining (7.30), (7.31) and (7.32) and defining ${\mathcal {E}}_{[{\mathcal {K}},D]} = \sum _{i=1}^3(\Sigma _i+\text {h.c.})$ proves the claim. $\square$

#### Corollary 7.8

Assume the exponents $\alpha , \beta$ satisfy (5.6). Let $m_0\in {\mathbb {R}}$ be such that $m_0 \beta =\alpha$ ($3< m_0 < 5$ from (5.6)). Then, there exists a constant $C>0$ such that

7.33 $$\begin{aligned} \begin{aligned}&e^{-sD} {\mathcal {K}}e^{sD} \\&\quad \le C {\mathcal {K}}+C{\mathcal {V}}_N+ C{\mathcal {V}}_{N,L} + CN^{5\beta /4+\kappa }{\mathcal {K}}_{\le N^{3\beta /2}} \\&\quad \quad + C \sum _{j=3}^{2\lfloor m_0\rfloor -1}N^{ j\beta /2 + 3\beta /2+2\kappa -1}\Big [{\mathcal {K}}_L + N^{2\beta }({\mathcal {N}}_{\ge \frac{1}{2} N^\alpha } +1)\Big ]({\mathcal {N}}_{\ge \frac{1}{2} N^{j\beta /2}}+1)\\&\quad \quad +C N^{ \alpha +\beta +2\kappa -1}\Big [{\mathcal {K}}_L + N^{2\beta }({\mathcal {N}}_{\ge \frac{1}{2} N^\alpha } +1)\Big ]({\mathcal {N}}_{\ge \frac{1}{2} N^{\lfloor m_0 \rfloor \beta }} +1) + CN^{13\beta /4 +\kappa } \end{aligned} \end{aligned}$$

for all $s\in [-1;1]$ and for all $N\in {\mathbb {N}}$ sufficiently large.

#### Proof

Given $\xi \in {\mathcal {F}}_+^{\le N}$, we define $\varphi _\xi (s) = \langle \xi , e^{-sD} {\mathcal {K}}e^{sD}\xi \rangle$. Differentiation yields

$$\begin{aligned} \partial _s \varphi _\xi (s) = \langle \xi , e^{-sD} [{\mathcal {K}}, D]e^{sD}\xi \rangle , \end{aligned}$$

s.t., to bound the derivative of $\varphi _\xi$, we can apply Proposition 7.7. Arguing exactly as in (7.22), we obtain with $\sup _{x\in \Lambda } |f_N(x)|\le 1$ the operator inequality

$$\begin{aligned}\begin{aligned}&\pm \frac{1}{2N} \sum _{\begin{array}{c} u\in \Lambda _+^*, v,w\in P_L:\\ v+u, w-u\ne 0 \end{array}} N^{\kappa } ({\widehat{V}}(./N^{1-\kappa })*{\widehat{f}}_N)(u) a^*_{v+u} a^*_{w-u}a_va_w \le C {\mathcal {V}}_N + C{\mathcal {V}}_{N,L} . \end{aligned}\end{aligned}$$

Now, the claim follows from the bound (7.24) (choosing $\delta =1$), the previous bound and an application of Corollaries 7.6, 7.4, Lemmas 7.1, 7.2 and the operator bound ${\mathcal {N}}_{\ge \frac{1}{2} N^\alpha }\le 4 N^{-2\alpha }{\mathcal {K}}$, by Gronwall’s Lemma. $\square$

### Action of Quartic Renormalization on Excitation Hamiltonian

We compute now the main contributions to ${\mathcal {M}}_N= e^{-D} {\mathcal {J}}_{N}^\text {eff} e^{D}$. From (4.5) and recalling that $[{\mathcal {N}}_+ , D] = 0$, we can decompose

7.34 $$\begin{aligned} {\mathcal {M}}_{N}= 4\pi \mathfrak {a}_0 N^{1+\kappa } -4\pi \mathfrak {a}_0 N^{\kappa -1}{\mathcal {N}}_+^2/N +{\mathcal {M}}_{N}^{(2)}+{\mathcal {M}}_{N}^{(3)}+{\mathcal {M}}_{N}^{(4)} \end{aligned}$$

where the operators ${\mathcal {M}}_{N}^{(i)}, i= 2,3,4,$ are defined by

7.35 $$\begin{aligned} \begin{aligned} {\mathcal {M}}_{N}^{(2)} =&\; 8\pi {\mathfrak {a}}_0N^\kappa \sum _{p\in P_H^c} e^{-D}b^*_pb_p e^D + 4\pi \mathfrak {a}_0 N^{\kappa }\sum _{p\in P^c_H} e^{-D} \big [ b^*_p b^*_{-p} + b_p b_{-p} \big ] e^D \\ {\mathcal {M}}_{N}^{(3)} =&\; \frac{8\pi {\mathfrak {a}}_0N^\kappa }{\sqrt{N}}\sum _{\begin{array}{c} p\in P_H^c , q\in P_L:\\ p+q\ne 0 \end{array}} e^{-D}\big [ b^*_{p+q}a^*_{-p}a_q+ \text {h.c.}\big ]e^D ,\\ {\mathcal {M}}_{N}^{(4)} =&\; e^{-D}{\mathcal {H}}_N e^D = e^{-D}{\mathcal {K}}e^D +e^{-D}{\mathcal {V}}_N e^D. \end{aligned} \end{aligned}$$

#### Analysis of ${\mathcal {M}}_N^{(2)}$

In this section, we determine the main contributions to ${\mathcal {M}}_N^{(2)}$, defined in (7.35) by

7.36 $$\begin{aligned} {\mathcal {M}}_N^{(2)} = 8\pi {\mathfrak {a}}_0N^\kappa \sum _{p\in P_H^c} e^{-D}b^*_pb_p e^D + 4\pi \mathfrak {a}_0 N^{\kappa }\sum _{p\in P^c_H} e^{-D} \big [ b^*_p b^*_{-p} + b_p b_{-p} \big ] e^D \end{aligned}$$

The main result of this section is the following proposition.

##### Proposition 7.9

Assume the exponents $\alpha , \beta$ satisfy (5.6). Then

7.37 $$\begin{aligned} {\mathcal {M}}_N^{(2)}= 8\pi {\mathfrak {a}}_0N^\kappa \sum _{p\in P_H^c} \Big [ b^*_pb_p +\frac{1}{2} b^*_p b^*_{-p} +\frac{1}{2} b_p b_{-p} \Big ] + {\mathcal {E}}_{{\mathcal {M}}_N}^{(2)} \end{aligned}$$

and there exists a constant $C>0$ such that

7.38 $$\begin{aligned} \begin{aligned} \pm e^{A}e^{D} {\mathcal {E}}_{{\mathcal {M}}_N}^{(2)} e^{-D} e^{-A}&\le C N^{-\beta -2\kappa } {\mathcal {K}}+ CN^\kappa \end{aligned} \end{aligned}$$

for all $N\in {\mathbb {N}}$ sufficiently large.

##### Proof

We start with the identity

7.39 $$\begin{aligned} \begin{aligned}&{\mathcal {M}}_N^{(2)} - 8\pi {\mathfrak {a}}_0N^\kappa \sum _{p\in P_H^c} \Big [ b^*_pb_p +\frac{1}{2} b^*_p b^*_{-p} +\frac{1}{2} b_p b_{-p} \Big ] \\&\quad = 8\pi {\mathfrak {a}}_0N^\kappa \int _0^1dt \;\sum _{p\in P_H^c} e^{-tD}\big [ b^*_pb_p +\frac{1}{2} b^*_p b^*_{-p} +\frac{1}{2} b_p b_{-p} , D_1 \big ]e^{tD} +\text {h.c.} \end{aligned} \end{aligned}$$

and a straight-forward computation shows that

$$\begin{aligned} \begin{aligned}&\big [ b^*_pb_p +\frac{1}{2} b^*_p b^*_{-p} +\frac{1}{2} b_p b_{-p}, a_{v+r}^* a_{w-r}^* a_w a_v \big ] \\&\quad = b^*_{v+r}a^*_{w-r}a_v b_w \big ( \delta _{p,v+r} + \delta _{p,w-r}- \delta _{p,v} - \delta _{p,w}\big ) \\&\qquad - \frac{1}{2} b^*_{v+r}b^*_{w-r} \big (\delta _{p,w}\delta _{-p,v} + \delta _{-p,w}\delta _{p,v}) + \frac{1}{2}b_{v}b_{w} (\delta _{p,w-r}\delta _{-p,v+r} + \delta _{-p,w-r}\delta _{p,v+r}\big ) \\&\qquad - \frac{1}{2}b^*_{v+r}b^*_{w-r}\big ( a^*_{-p}a_w\delta _{p,v} + a^*_{p}a_w\delta _{-p,v} + a^*_{-p}a_v\delta _{p,w} + a^*_{p}a_v\delta _{-p,w} \big )\\&\qquad + \frac{1}{2}\big ( a^*_{w-r}a_{-p}\delta _{p,v+r} + a^*_{v+r}a_{-p}\delta _{p,w-r} + a^*_{w-r}a_{p}\delta _{-p,v+r} + a^*_{v+r}a_{p}\delta _{-p,w-r} \big ) b_v b_w. \end{aligned} \end{aligned}$$

As a consequence, we find that

7.40 $$\begin{aligned} \begin{aligned}&{\mathcal {M}}_N^{(2)} - 8\pi {\mathfrak {a}}_0N^\kappa \sum _{p\in P_H^c} \Big [ b^*_pb_p +\frac{1}{2} b^*_p b^*_{-p} +\frac{1}{2} b_p b_{-p} \Big ] = \int _0^1\!\!dt\; e^{-tD}\sum _{j =1}^5\big ( \text {V}_j + \text {h.c.}\big )e^{tD}, \end{aligned} \end{aligned}$$

where

7.41 $$\begin{aligned} \begin{aligned} \text {V}_1&= - \frac{8\pi {\mathfrak {a}}_0N^\kappa }{2N}\sum _{\begin{array}{c} r\in P_H, v \in P_L \end{array}} \eta _r b^*_{v+r}b^*_{-v-r}, \\ \text {V}_2&= \frac{8\pi {\mathfrak {a}}_0N^\kappa }{2N}\sum _{\begin{array}{c} r\in P_H, v\in P_L: \\ v+r \in P_H^c , v+r\ne 0 \end{array}}\eta _r b_v b_{-v} , \\ \text {V}_3&=\frac{8\pi {\mathfrak {a}}_0N^\kappa }{2N}\sum _{\begin{array}{c} r\in P_H, v,w\in P_L: \\ v+r,w-r\ne 0 \end{array}}\eta _r \big (-2 + \chi _{\{ r+v\in P_H^c\} }+ \chi _{\{ w-r\in P_H^c\} }\big ) b^*_{v+r}a^*_{w-r}a_v b_w , \\ \text {V}_4&=- \frac{8\pi {\mathfrak {a}}_0N^\kappa }{N}\sum _{\begin{array}{c} r\in P_H, v,w\in P_L: \\ v+r,w-r\ne 0 \end{array}}\eta _r b^*_{v+r}b^*_{w-r} a^*_{-v}a_w , \\ \text {V}_5&= \frac{8\pi {\mathfrak {a}}_0N^\kappa }{N}\sum _{\begin{array}{c} r\in P_H, v,w\in P_L: \\ r-w\in P_H^c, v+r,w-r\ne 0 \end{array}}\eta _r a^*_{v+r}a_{r-w} b_v b_w . \\ \end{aligned} \end{aligned}$$

Here, $\chi _{\{ p \in S\}}$ denotes as usual the characteristic function for the set $S\subset \Lambda _+^*$, evaluated at $p\in \Lambda _+^*$. Let us briefly explain how to bound the different contributions $\text {V}_1$ to $\text {V}_5$, defined in (7.41). Using Cauchy–Schwarz, the first two contributions are bounded by

$$\begin{aligned}\begin{aligned} \pm (\text {V}_1 + \text {V}_2)&\le C N^{2\kappa +3\beta -\alpha /2 -1} ({\mathcal {N}}_{\ge \frac{1}{2} N^\alpha }+1) + CN^{2\kappa +3\beta /2-1}({\mathcal {K}}_L+1) \end{aligned} \end{aligned}$$

where, for $\text {V}_2$, we used that $v+r\in P_H^c$ implies that $|r|\le N^{\alpha }+N^{\beta }$ and furthermore that $\sum _{N^\alpha \le |r|\le N^\alpha +N^\beta }|\eta _r|\le N^{\kappa +\beta }$. The contributions $\text {V}_3$ to $\text {V}_5$, on the other hand, can be controlled by

$$\begin{aligned} \begin{aligned}&|\langle \xi , (\text {V}_3 + \text {V}_4+\text {V}_5)\xi \rangle |\\&\quad \le \frac{CN^{\kappa }}{N}\sum _{\begin{array}{c} r\in P_H, v,w\in P_L: \\ v+r,w-r\ne 0 \end{array}} |\eta _r| \Vert a_{v+r}({\mathcal {N}}_{\ge \frac{1}{2} N^\alpha }+1)^{-1/2}a_{w-r} \xi \Vert \Vert a_v ({\mathcal {N}}_{\ge \frac{1}{2} N^\alpha }+1)^{1/2} a_w\xi \Vert \\&\qquad + \frac{CN^{\kappa }}{N}\sum _{\begin{array}{c} r\in P_H, v,w\in P_L: \\ v+r,w-r\ne 0 \end{array}} |\eta _r| \Vert a_{v+r}({\mathcal {N}}_{\ge \frac{1}{2} N^\alpha }+1)^{-1/2}a_{w-r}a_w \xi \Vert \Vert a_v ({\mathcal {N}}_{\ge \frac{1}{2} N^\alpha }+1)^{1/2} \xi \Vert \\&\qquad + \frac{CN^{\kappa }}{N}\sum _{\begin{array}{c} r\in P_H, v,w\in P_L: \\ v+r,w-r\ne 0 \end{array}}|\eta _r| \Vert a_{v+r}\xi \Vert \Vert a_va_w a_{w-r}\xi \Vert \\&\quad \le CN^{2\kappa +3\beta /2-\alpha /2} \langle \xi , ({\mathcal {N}}_{\ge \frac{1}{2} N^\alpha }+1)\xi \rangle \le C N^\kappa \langle \xi , ({\mathcal {N}}_{\ge \frac{1}{2} N^\alpha }+1)\xi \rangle \end{aligned} \end{aligned}$$

for any $\xi \in {\mathcal {F}}_+^{\le N}$. In conclusion (since $2\kappa + 3\beta -\alpha /2-1 < \kappa$ from (5.6)), we have proved that

$$\begin{aligned} \pm \sum _{j =1}^5\big ( \text {V}_j + \text {h.c.}\big )\le CN^{2\kappa +3\beta /2-1} {\mathcal {K}}_L + CN^\kappa ({\mathcal {N}}_{\ge \frac{1}{2} N^\alpha } + 1) . \end{aligned}$$

Now, applying this bound together with (7.40), Lemmas 4.2, 4.3, 7.1, 7.2 and the operator inequality ${\mathcal {N}}_{\ge \frac{1}{2} N^\alpha }\le 4 N^{-2\alpha }{\mathcal {K}}$ proves the claim. $\square$

#### Analysis of ${\mathcal {M}}_N^{(3)}$

In this section, we determine the main contributions to ${\mathcal {M}}_N^{(3)}$, defined in (7.35) by

7.42 $$\begin{aligned} {\mathcal {M}}_N^{(3)}= \frac{8\pi {\mathfrak {a}}_0 N^\kappa }{\sqrt{N}}\sum _{\begin{array}{c} p\in P_H^c, q\in P_L: \\ p+q\ne 0 \end{array}} e^{-D}\big (b^*_{p+q}a^*_{-p}a_q+ \text {h.c.}\big )e^D . \end{aligned}$$

##### Proposition 7.10

Assume the exponents $\alpha , \beta$ satisfy (5.6). Then, we have that

7.43 $$\begin{aligned} {\mathcal {M}}_N^{(3)}= \frac{8\pi {\mathfrak {a}}_0 N^\kappa }{\sqrt{N}}\sum _{\begin{array}{c} p\in P_H^c, q\in P_L: \\ p+q\ne 0 \end{array}} \big (b^*_{p+q}a^*_{-p}a_q+ h.c. \big ) + {\mathcal {E}}_{{\mathcal {M}}_N}^{(3)} \end{aligned}$$

and there exists a constant $C>0$ such that

7.44 $$\begin{aligned} \begin{aligned}&\pm e^{A}e^{D} {\mathcal {E}}_{{\mathcal {M}}_N}^{(3)} e^{-D} e^{-A} \\&\quad \le CN^{-\beta }{\mathcal {K}}+ CN^{\alpha + \beta /2+2\kappa -1 }{\mathcal {K}}({\mathcal {N}}_{\ge \frac{1}{2} N^\alpha }+1) + CN^{\alpha + \beta /2+2\kappa } \end{aligned} \end{aligned}$$

for all $N\in {\mathbb {N}}$ sufficiently large.

##### Proof

Let us define the operator $\text {Y}:{\mathcal {F}}_+^{\le N}\rightarrow {\mathcal {F}}_+^{\le N}$ by

7.45 $$\begin{aligned} \text {Y} = \frac{8\pi {\mathfrak {a}}_0 N^\kappa }{\sqrt{N}}\sum _{\begin{array}{c} p\in P_H^c, q\in P_L: \\ p+q\ne 0 \end{array}} \big (b^*_{p+q}a^*_{-p}a_q+ \text {h.c.}\big ), \end{aligned}$$

so that ${\mathcal {M}}_N^{(3)} = e^{-D}\text {Y}e^D$. We recall the definition (7.1) and observe that

7.46 $$\begin{aligned} e^{-D}\text {Y}e^D- \text {Y} = \int _0^1 ds\; e^{-sD} [\text {Y}, D_1] e^{sD} + \text {h.c.}. \end{aligned}$$

This implies that it is enough to control the commutator $$ [\text {Y}, D_1]$$ after conjugation with $$ e^{tD}$$, for any $$t\in [-1;1] $$. Note that, if $$ p\in P_H^c, q\in P_L, r\in P_H$$ and $$v,w \in P_L$$, we have $$ |v+r|\ge N^\alpha -N^\beta>\frac{1}{2} N^\alpha >N^\beta $$ s.t. $$[a^*_{-p}a_q, a^*_{v+r}a^*_{w-r}] = 0 $$, for $$N\in {\mathbb {N}}$$ large enough. Then, a lengthy but straightforward calculation shows that

$$\begin{aligned} \begin{aligned} {[}b^*_{p+q}a^*_{-p}a_q, a^*_{v+r}a^*_{w-r}a_va_w]&= -b^*_{v+r}a^*_{w-r}a_q (\delta _{-p, w}\delta _{p+q,v} + \delta _{-p,v}\delta _{p+q,w})\\&\quad -b^*_{p+q}a^*_{v+r}a^*_{w-r}a_q (a_w \delta _{-p,v}+ a_v\delta _{-p,w} ) \\&\quad -b^*_{-p}a^*_{v+r}a^*_{w-r}a_q (a_w\delta _{p+q,v}+a_v\delta _{p+q,w}) \end{aligned}\end{aligned}$$

and

$$\begin{aligned}\begin{aligned} {[}a^*_qa_{-p}b_{p+q}, a^*_{v+r}a^*_{w-r}a_va_w]&= a^*_q a_v b_w \delta _{-p,w-r}\delta _{p+q,v+r} + a^*_q a_v b_w \delta _{-p,v+r}\delta _{p+q,w-r}\\&\quad + a^*_qa^*_{w-r}a_va_wb_{p+q}\delta _{-p,v+r}+ a^*_qa^*_{v+r}a_va_wb_{p+q}\delta _{-p,w-r}\\&\quad - a^*_{v+r}a^*_{w-r}a_wa_{-p}b_{p+q}\delta _{q,v}- a^*_{v+r}a^*_{w-r}a_va_{-p}b_{p+q}\delta _{q,w}\\&\quad + a^*_qa^*_{w-r}a_{-p}a_vb_{w}\delta _{p+q,v+r}+ a^*_qa^*_{v+r}a_{-p}a_vb_{w}\delta _{p+q,w-r}. \end{aligned} \end{aligned}$$

As a consequence, we conclude that

7.47 $$\begin{aligned} \begin{aligned} {[}\text {Y}, D_1]+\text {h.c.}&=\sum _{i=1}^6 (\Psi _{i}+\text {h.c.}) , \end{aligned} \end{aligned}$$

where

7.48 $$\begin{aligned} \begin{aligned} \Psi _{1}&= - \frac{8\pi {\mathfrak {a}}_0 N^\kappa }{N^{3/2}} \sum _{\begin{array}{c} r\in P_H, v,w\in P_L : \\ v+w \in P_L \end{array}}^* \eta _r b^*_{v+r}a^*_{w-r}a_{v+w} , \\ \Psi _{2}&= \frac{8\pi {\mathfrak {a}}_0 N^\kappa }{N^{3/2}} \sum _{\begin{array}{c} r\in P_H, v,w\in P_L:\\ v+r, r - w \in P_H^c, v+w \in P_L \end{array}}^* \eta _r a^*_{v+w} a_v b_w,\\ \Psi _{3}&= -\frac{16\pi {\mathfrak {a}}_0 N^\kappa }{N^{3/2}} \sum _{r\in P_H, q, v,w\in P_L}^*\eta _r b^*_{q-v}a^*_{v+r}a^*_{w-r}a_qa_w, \\ \Psi _{4}&= \frac{8\pi {\mathfrak {a}}_0 N^\kappa }{N^{3/2}} \sum _{\begin{array}{c} r\in P_H, q,v,w\in P_L:\\ v+r\in P_H^c \end{array}}^* \eta _r a^*_qa^*_{w-r}a_va_wb_{q-v-r}, \\ \Psi _{5}&=\frac{8\pi {\mathfrak {a}}_0 N^\kappa }{N^{3/2}} \sum _{\begin{array}{c} r\in P_H, q,v,w\in P_L:\\ v+r-q \in P_H^c \end{array}}^* \eta _r a^*_qa^*_{w-r}a_va_wb_{q-v-r}, \\ \Psi _{6}&= - \frac{8\pi {\mathfrak {a}}_0 N^\kappa }{N^{3/2}} \sum _{p\in P_H^c, r\in P_H, v,w\in P_L}^* \eta _r a^*_{v+r}a^*_{w-r}a_wa_{-p}b_{p+v}. \end{aligned} \end{aligned}$$

Let us explain how to control the operators $\Psi _1$ to $\Psi _6$, defined in (7.48). We start with $\Psi _1$. Given $\xi \in {\mathcal {F}}_+^{\le N}$, we find that

$$\begin{aligned} \begin{aligned}&|\langle \xi , \Psi _1\xi \rangle | \\&\quad = \bigg | \frac{8\pi {\mathfrak {a}}_0 N^\kappa }{N^{3/2}} \sum _{r\in P_H, v,w\in P_L}^* \eta _r \langle \xi , b^*_{v+r}a^*_{w-r}a_{v+w}\xi \rangle \bigg | \\&\quad \le \frac{C N^\kappa }{N^{3/2}} \sum _{r\in P_H, v,w\in P_L}^*|\eta _r| \Vert ({\mathcal {N}}_{\ge \frac{1}{2} N^\alpha }+1)^{-1/2}a_{v+r}a_{w-r} \xi \Vert \Vert ({\mathcal {N}}_{\ge \frac{1}{2} N^\alpha }+1)^{1/2}a_{v+w} \xi \Vert \\&\quad \le C N^{3\beta + 2\kappa -\alpha /2-1} \langle \xi , ({\mathcal {N}}_{\ge \frac{1}{2} N^\alpha }+1)\xi \rangle \le C N^{3\beta /2 + \kappa -1} \langle \xi , ({\mathcal {N}}_{\ge \frac{1}{2} N^\alpha }+1)\xi \rangle . \end{aligned} \end{aligned}$$

The contribution $\Psi _2$ can be bounded by

$$\begin{aligned} \begin{aligned} |\langle \xi , \Psi _2\xi \rangle |&= \bigg | \frac{8\pi {\mathfrak {a}}_0 N^\kappa }{N^{3/2}} \sum _{\begin{array}{c} r\in P_H, v,w\in P_L:\\ v+r\in P_H^c \end{array}}^* \eta _r \langle \xi a^*_{v+w} a_v b_w\xi \rangle \bigg | \\&\le C N^{\beta /2 + \kappa -1} \langle \xi , {\mathcal {K}}_{\le 2N^\beta }\xi \rangle \sum _{\begin{array}{c} N^\alpha \le |r|\le N^{\alpha }+N^\beta \end{array} } |\eta _r|\le C N^{3\beta /2 + 2\kappa -1} \langle \xi , {\mathcal {K}}_{\le 2N^\beta }\xi \rangle . \end{aligned}\end{aligned}$$

Notice here, that we used that $|r|\le N^\alpha +N^\beta$ if $r+v\in P_H^c$ and $v\in P_L$. Next, we apply as usual Cauchy–Schwarz to estimate the terms $\Psi _3$ to $\Psi _5$ by

$$\begin{aligned} \begin{aligned}&| \langle \xi , \Psi _3\xi \rangle + \langle \xi , \Psi _4\xi \rangle + \langle \xi , \Psi _5\xi \rangle | \\&\quad \le C N^{3\beta + 2\kappa -\alpha /2} \langle \xi , ({\mathcal {N}}_{\ge \frac{1}{2} N^\alpha }+1)\xi \rangle \le C N^{3\beta /2 + \kappa } \langle \xi , ({\mathcal {N}}_{\ge \frac{1}{2} N^\alpha }+1)\xi \rangle \end{aligned}\end{aligned}$$

for all $\alpha >3\beta +2\kappa$. Finally, the term $\Psi _6$ can be controlled by

$$\begin{aligned} \begin{aligned} | \langle \xi , \Psi _6\xi \rangle |&= \bigg | \frac{8\pi {\mathfrak {a}}_0 N^\kappa }{N^{3/2}} \sum _{p\in P_H^c, r\in P_H, v,w\in P_L}^* \eta _r \langle \xi , a^*_{v+r}a^*_{w-r}a_wa_{-p}b_{p+v} \xi \rangle \bigg | \\&\le CN^{\kappa -3/2} \sum _{p\in P_H^c, r\in P_H, v,w\in P_L}^*|w|^{-1} \Vert ({\mathcal {N}}_{\ge N^\alpha /2} + 1)^{-1/2}a_{v+r}a_{w-r} \xi \Vert \\&\quad \times |w||\eta _r|\Vert a_wa_{-p}b_{p+v} ({\mathcal {N}}_{\ge N^\alpha /2} + 1)^{1/2} \xi \Vert \\&\le CN^{\alpha + \beta /2+2\kappa -1 } \langle \xi , {\mathcal {K}}_L ({\mathcal {N}}_{\ge \frac{1}{2} N^\alpha }+1)\xi \rangle + CN^{\alpha + \beta /2+2\kappa }\langle \xi , ({\mathcal {N}}_{\ge \frac{1}{2} N^\alpha }+1)\xi \rangle . \end{aligned} \end{aligned}$$

In conclusion, the previous estimates show that

$$\begin{aligned} \begin{aligned} \pm \bigg [ \sum _{i=1}^6 (\Psi _{i}+\text {h.c.}) \bigg ]&\le C N^{3\beta /2 + 2\kappa -1}{\mathcal {K}}_{\le 2N^\beta } + CN^{\alpha + \beta /2+2\kappa -1 }{\mathcal {K}}_L ({\mathcal {N}}_{\ge \frac{1}{2} N^\alpha }+1)\\&\quad + CN^{\alpha + \beta /2+2\kappa }({\mathcal {N}}_{\ge \frac{1}{2} N^\alpha }+1), \end{aligned} \end{aligned}$$

so that, together with (7.46) and (7.47), an application of the Lemmas 4.2, 4.3, 7.1, 7.2 and the operator bound ${\mathcal {N}}_{\ge \frac{1}{2} N^\alpha }\le 4N^{-2\alpha }{\mathcal {K}}$ proves the claim. $\square$

#### Analysis of ${\mathcal {M}}_N^{(4)}$

In this section, we determine the main contributions to ${\mathcal {M}}_N^{(4)} = e^{-D}{\mathcal {H}}_N e^D$, defined in (7.35). To this end, we start with the observation that

7.49 $$\begin{aligned} {\mathcal {M}}_N^{(4)} = {\mathcal {H}}_N + \int _0^1ds\; e^{-sD} \Big ( [{\mathcal {K}}, D_1] + [{\mathcal {V}}_N, D_1]\Big ) e^{sD} +\text {h.c.}, \end{aligned}$$

with $D_1$ defined in (7.1). By Propositions 7.5 and 7.7, this implies that

7.50 $$\begin{aligned} \begin{aligned} {\mathcal {M}}_N^{(4)}&= {\mathcal {H}}_N - \frac{N^\kappa }{2N}\sum _{\begin{array}{c} r\in \Lambda ^*, v,w\in P_L: \\ v+r, w-r\ne 0 \end{array}}\int _0^1 ds\; {\widehat{V}}(r/N^{1-\kappa }) e^{-sD}\big (a^*_{v+r}a^*_{w-r}a_va_w+\text {h.c.}\big )e^{sD}\\&\quad + \int _0^1ds\; e^{-sD} \Big ( {\mathcal {E}}_{ [{\mathcal {K}}, D] }+ {\mathcal {E}}_{[{\mathcal {V}}_N, D]}\Big ) e^{sD}, \end{aligned} \end{aligned}$$

where we used that ${\widehat{V}}(\cdot /N^{1-\kappa })*({\widehat{f}}_N-\eta /N)(r) = {\widehat{V}}(\cdot /N^{1-\kappa })(r)$ for all $r\in \Lambda _+^*$. Moreover, the operators ${\mathcal {E}}_{[{\mathcal {V}}_N,D]}$ and ${\mathcal {E}}_{[{\mathcal {K}}, D]}$ are explicitly given by

7.51 $$\begin{aligned} \begin{aligned} {\mathcal {E}}_{[{\mathcal {V}}_N,D]} = \sum _{i=1}^4 \big (\Phi _i+\text {h.c.} \big ) ,  {\mathcal {E}}_{[{\mathcal {K}},D]} = \sum _{j=1}^3 \big (\Sigma _j+\text {h.c.}\big ) \end{aligned} \end{aligned}$$

where we recall the definitions (7.19) and (7.26). Let us analyze the different contributions in (7.50), separately. We start with the second term on the r.h.s. of (7.50).

##### Proposition 7.11

Assume the exponents $\alpha , \beta$ satisfy (5.6). Then, we have

7.52 $$\begin{aligned} \begin{aligned}&\frac{1}{2N}\sum _{\begin{array}{c} u\in \Lambda ^*, p, q\in P_L: \\ p+u, q-u\ne 0 \end{array}} N^\kappa {\widehat{V}}(r/N^{1-\kappa }) e^{-sD}\big (a^*_{p+u}a^*_{q-u}a_p a_q+h.c. \big )e^{sD} \\&\quad = \frac{1}{2N}\sum _{\begin{array}{c} u\in \Lambda ^*, p, q\in P_L: \\ p+u, q-u\ne 0 \end{array}} N^\kappa {\widehat{V}}(r/N^{1-\kappa }) \big (a^*_{p+u}a^*_{q-u}a_p a_q+h.c. \big )\\&\qquad + \frac{s}{N} \sum ^*_{\begin{array}{c} u\in \Lambda ^*, v, w \in P_L:\\ v+u, w-u\in P_L \end{array}} N^{\kappa } ({\widehat{V}}(./N^{1-\kappa })*\eta /N)(u) a^*_{v+u} a^*_{w-u}a_va_w+ {\mathcal {E}}_1(s) + {\mathcal {E}}_2(s) \end{aligned} \end{aligned}$$

and there exists a constant $C>0$ s.t. ${\mathcal {E}}_1(s)$ and ${\mathcal {E}}_2(s)$ satisfy

7.53 $$\begin{aligned} \begin{aligned} \pm {\mathcal {E}}_1(s)&\le C(N^{\alpha +\beta +2\kappa -1} +N^{-3\beta -3\kappa }) {\mathcal {K}}+ CN^{2\beta +\kappa },\\ \pm {\mathcal {E}}_2(s)&\le CN^{\beta +\kappa -1}{\mathcal {K}}_L({\mathcal {N}}_{\ge \frac{1}{2}N^\alpha }+1) + C(N^{-\beta -\kappa } + CN^{3\beta /2 + \kappa /2-1}) \int _0^s dt\; e^{-tD} {\mathcal {V}}_N e^{tD}\\&\quad + CN^{2\beta +2\kappa -1}\int _0^s dt\; e^{-tD} {\mathcal {K}}_{\le 2N^\beta }({\mathcal {N}}_{\ge \frac{1}{2} N^\alpha }+1)e^{tD}, \end{aligned}\end{aligned}$$

for all $\delta >0$, $s\in [-1;1]$ and for all $N\in {\mathbb {N}}$ sufficiently large.

##### Proof

For definiteness, let us denote by $\text {W}:{\mathcal {F}}_+^{\le N}\rightarrow {\mathcal {F}}_+^{\le N}$ the operator

7.54 $$\begin{aligned} \text {W} = \frac{1}{2N}\sum _{\begin{array}{c} u\in \Lambda ^*, p, q\in P_L: \\ p+u, q-u\ne 0 \end{array}} N^\kappa {\widehat{V}}(u/N^{1-\kappa }) \big (a^*_{p+u}a^*_{q-u}a_p a_q+\text {h.c.}\big ) \end{aligned}$$

and consider the identity

7.55 $$\begin{aligned} \begin{aligned}&e^{-sD}\text {W} e^{sD} - \text {W} \\&\quad = \int _0^s dt\; e^{-tD} [ \text {W}, D_1] e^{tD} +\text {h.c.}\\&\quad = \frac{1}{2N}\int _0^s dt\;\sum _{\begin{array}{c} u\in \Lambda ^*, p, q\in P_L: \\ p+u, q-u\ne 0 \end{array}} N^\kappa {\widehat{V}}(r/N^{1-\kappa }) e^{-tD} \Big [\big (a^*_{p+u}a^*_{q-u}a_p a_q+\text {h.c.}\big ), D_1\Big ]e^{tD} +\text {h.c.} \end{aligned} \end{aligned}$$

Now, observe that

$$\begin{aligned} {[}a_p, a^*_{v+r} ] = [a_q, a^*_{v+r} ] = [a_p, a^*_{w-r} ] =[a_q, a^*_{w-r} ] =0 \end{aligned}$$

for all $p,q \in P_L$ and $r\in P_H$, $v,w\in P_L$ and $N\in {\mathbb {N}}$ sufficiently large. Then, proceeding as in the proof of Proposition 7.5, we obtain

7.56 $$\begin{aligned} \begin{aligned}&[a^*_{p+u}a^*_{q-u} a_p a_{q}, a^*_{v+r} a^*_{w-r} a_v a_w] \\&\quad = - a^*_{v+r} a^*_{w-r}a^*_{q-u} a_w a_{p} a_{q}\delta _{p+u, v} - a^*_{v+r} a^*_{w-r}a^*_{p+u} a_w a_{p} a_{q} \delta _{q-u, v}\\&\qquad - a^*_{v+r} a^*_{w-r} a_v a^*_{q-u} a_{p} a_{q}\delta _{p+u, w} - a^*_{v+r} a^*_{w-r} a_v a^*_{p+u} a_{p} a_{q} \delta _{q-u, w}. \end{aligned} \end{aligned}$$

and

7.57 $$\begin{aligned} \begin{aligned}&[a^*_{p}a^*_q a_{p-u} a_{q+u}, a^*_{v+r} a^*_{w-r} a_v a_w] \\&\quad = a^*_{p}a^*_q a_{q+u} a^*_{w-r} a_v a_w\delta _{p-u, v+r} + a^*_{p}a^*_q a_{p-u} a^*_{w-r} a_v a_w \delta _{q+u, v+r}\\&\qquad + a^*_{p}a^*_q a^*_{v+r}a_{q+u} a_v a_w\delta _{p-u, w-r} + a^*_{p}a^*_q a^*_{v+r} a_{p-u} a_v a_w \delta _{q+u, w-r}\\&\qquad - a^*_{v+r} a^*_{w-r}a^*_q a_w a_{p-u} a_{q+u}\delta _{p, v} - a^*_{v+r} a^*_{w-r}a^*_{p} a_w a_{p-u} a_{q+u} \delta _{q, v}\\&\qquad - a^*_{v+r} a^*_{w-r} a_v a^*_q a_{p-u} a_{q+u}\delta _{p, w} - a^*_{v+r} a^*_{w-r} a_v a^*_{p} a_{p-u} a_{q+u} \delta _{q, w}. \end{aligned} \end{aligned}$$

Combining the last two identities and putting non-normally ordered contributions into normal order, we find that

7.58 $$\begin{aligned} \begin{aligned} {[}\text {W}, D_1]+\text {h.c.} =&\; \frac{1}{N} \sum ^*_{\begin{array}{c} u\in \Lambda ^*, v, w \in P_L:\\ v+u, w-u\in P_L \end{array}} N^{\kappa } ({\widehat{V}}(./N^{1-\kappa })*\eta /N)(u) a^*_{v+u} a^*_{w-u}a_va_w \\&+\sum _{j=1}^6 \big ( \zeta _{j}+\text {h.c.}\big ), \end{aligned}\end{aligned}$$

where

7.59 $$\begin{aligned} \zeta _{1}&= - \frac{1}{2N^{2}}\sum ^*_{\begin{array}{c} u \in \Lambda ^*, v,w \in P_L: \nonumber \\ v+u, w-u\in P_L,\nonumber \\ r \in P_H^c\cup \{0\} \end{array}} N^{\kappa } {\widehat{V}}((u-r)/N^{1-\kappa })\eta _r a^*_{v+u} a_{w-u}^* a_va_w,\nonumber \\ \zeta _{2}&= -\frac{1}{2N^{2}}\sum ^*_{\begin{array}{c} u\in \Lambda ^*, r\in P_{H},\nonumber \\ v,w\in P_{L}:\nonumber \\ w-u, v+u\in P_L \end{array}}N^\kappa {\widehat{V}}(u/N^{1-\kappa }) \eta _r a^*_{v+r} a^*_{w-r} a_{w-u} a_{v+u} ,\nonumber \\ \zeta _{3}&= -\frac{1}{2N^{2}}\sum ^*_{\begin{array}{c} u\in \Lambda ^*, r\in P_{H},\nonumber \\ v,w\in P_{L} \end{array}}N^\kappa {\widehat{V}}(u/N^{1-\kappa }) \eta _r a^*_{v+r} a^*_{w-r} a_{w-u} a_{v+u} ,\nonumber \\ \zeta _{4}&= -\frac{1}{N^{2}}\sum ^*_{\begin{array}{c} u\in \Lambda ^*, r\in P_{H},\nonumber \\ v,w,q\in P_L:\nonumber \\ v-u\in P_{L} \end{array}}N^\kappa {\widehat{V}}(u/N^{1-\kappa }) \eta _r a^*_{v+r} a^*_{w-r}a^*_{q-u} a_w a_{v-u} a_{q},\nonumber \\ \zeta _{5}&= \frac{1}{N^{2}}\sum ^*_{\begin{array}{c} u\in \Lambda ^*, r\in P_{H},\nonumber \\ v,w,q \in P_L:\nonumber \\ v+r+u\in P_{L} \end{array}}N^\kappa {\widehat{V}}(u/N^{1-\kappa }) \eta _r a^*_{v+r+u}a^*_q a^*_{w-r}a_{q+u} a_v a_w ,\nonumber \\ \zeta _{6}&= -\frac{1}{N^{2}}\sum ^*_{\begin{array}{c} u\in \Lambda ^*, r\in P_{H},\nonumber \\ v,w,q\in P_{L} \end{array}}N^\kappa {\widehat{V}}(u/N^{1-\kappa }) \eta _r a^*_{v+r} a^*_{w-r}a^*_q a_w a_{v-u} a_{q+u}.\nonumber \\ \end{aligned}$$

Let us briefly explain how to control the operators $\zeta _{1}$ to $\zeta _{6}$, defined in (7.2.3).

Noting that $v+u\in P_L$ implies $|u|\le 2N^\beta$ whenever $v\in P_L$, the first two contributions $\zeta _1$ and $\zeta _2$ in (7.2.3) can be controlled by

7.60 $$\begin{aligned} \begin{aligned}&|\langle \xi , \zeta _1\xi \rangle | +|\langle \xi , \zeta _2\xi \rangle |\\&\quad \le \frac{CN^\kappa }{2N^{2}}\sum ^*_{\begin{array}{c} u \in \Lambda ^*, v,w \in P_L: \\ v+u, w-u\in P_L,\\ r \in P_H^c\cup \{0\} \end{array}} | \eta _r| \frac{|w-u|}{|v|}\Vert a_{v+u} a_{w-u} \xi \Vert \frac{|v|}{|w-u|}\Vert a_va_w \xi \Vert \\&\qquad +\frac{C N^\kappa }{2N^{2}}\sum ^*_{\begin{array}{c} u\in \Lambda ^*, r\in P_{H},\\ v,w\in P_{L}:\\ w-u, v+u\in P_L \end{array}} |\eta _r| \Vert a_{v+r} ({\mathcal {N}}_{\ge \frac{1}{2}N^\alpha }+1)^{-1/2}a_{w-r} \xi \Vert \\&\qquad \times \Vert a_{w-u} ({\mathcal {N}}_{\ge \frac{1}{2}N^\alpha }+1)^{1/2}a_{v+u} \xi \Vert \\&\quad \le CN^{\alpha +\beta +2\kappa -1} \langle \xi , {\mathcal {K}}_{\le 2N^\beta }\xi \rangle + N^{7\beta /2+2\kappa -\alpha /2-1}\langle \xi , ({\mathcal {N}}_{\ge \frac{1}{2}N^\alpha }+1)\xi \rangle \\&\qquad + N^{7\beta /2+2\kappa -\alpha /2-2}\langle \xi , {\mathcal {K}}_{\le 2N^\beta } ({\mathcal {N}}_{\ge \frac{1}{2}N^\alpha }+1)\xi \rangle \\&\quad \le CN^{\alpha +\beta +2\kappa -1} \langle \xi , {\mathcal {K}}_{\le 2N^\beta }\xi \rangle + CN^{2\beta +\kappa -1}\langle \xi , ({\mathcal {N}}_{\ge \frac{1}{2}N^\alpha }+1)\xi \rangle . \end{aligned} \end{aligned}$$

By switching to position space, the term $\zeta _3$ can be bounded by

$$\begin{aligned}&|\langle \xi , \zeta _3\xi \rangle | \\&\quad \le CN^{3\beta /2+ \kappa -\alpha /2-1} \bigg (\int _{\Lambda ^2}dxdy\; N^{2-2\kappa }V(N^{1-\kappa }(x-y)) \Vert \check{a}_x\check{a}_y\xi \Vert ^2\bigg )^{1/2}\\&\qquad \times \bigg (\int _{\Lambda ^2}dxdy\; N^{2-2\kappa }V(N^{1-\kappa }(x-y)) \sum _{r\in P_H, w\in P_L}\Big \Vert \sum _{v\in P_L} e^{ivx} a_{v+r}a_{w-r}\xi \Big \Vert ^2\bigg )^{1/2}\\&\quad \le CN^{3\beta /2+ \kappa -\alpha /2-1} \Vert {\mathcal {V}}_N^{1/2}\xi \Vert \bigg (N^{\kappa -1}\int _{\Lambda }dx\; \sum _{r\in P_H, w\in P_L}\Big \Vert \sum _{v\in P_L} e^{ivx} a_{v+r}a_{w-r}\xi \Big \Vert ^2\bigg )^{1/2}\\&\quad \le CN^{3\beta /2 + \kappa /2-1}\langle \xi , {\mathcal {V}}_N\xi \rangle + CN^{3\beta /2 +\kappa /2} . \end{aligned}$$

We proceed similarly as above for the terms $\zeta _4$ and $\zeta _5$ which yields

7.61 $$\begin{aligned} \begin{aligned}&|\langle \xi , \zeta _4\xi \rangle | +|\langle \xi , \zeta _5\xi \rangle |\\&\quad \le \frac{CN^\kappa }{N^{2}}\sum ^*_{\begin{array}{c} u\in \Lambda ^*, r\in P_{H},\\ v,w,q\in P_L: v-u\in P_{L} \end{array}} |q|^{-1} |q-u| \Vert a_{v+r} ({\mathcal {N}}_{\ge \frac{1}{2} N^\alpha }+1)^{-1/2} a_{w-r}a_{q-u}\xi \Vert \\&\qquad \times |\eta _r| |q| |q-u|^{-1} \Vert a_w ({\mathcal {N}}_{\ge \frac{1}{2} N^\alpha }+1)^{1/2}a_{v-u} a_{q}\xi \Vert \\&\qquad + \frac{CN^\kappa }{N^{2}}\sum ^*_{\begin{array}{c} u\in \Lambda ^*, r\in P_{H},\\ v,w,q\in P_L:\\ v+r+u\in P_{L} \end{array}} \Big ( |q| |v|^{-1} \Vert a_{v+r+u}a_q a_{w-r}\xi \Vert \Big )\Big ( |\eta _r| |q|^{-1} |v|\Vert a_{q+u} a_v a_w\xi \Vert \Big )\\&\quad \le CN^{5\beta /2+2\kappa -\alpha /2 -1} \langle \xi , {\mathcal {K}}_{\le 3N^\beta }({\mathcal {N}}_{\ge \frac{1}{2} N^\alpha }+1)\xi \rangle \\&\quad \le C N^{\beta +\kappa -1} \langle \xi , {\mathcal {K}}_{\le 3N^\beta }({\mathcal {N}}_{\ge \frac{1}{2} N^\alpha }+1)\xi \rangle , \end{aligned}\end{aligned}$$

where, for $\zeta _5$, we used that $v+r+u\in P_L$ implies that $|u|\ge \frac{3}{4} N^\alpha$, and thus $|q+u|\ge \frac{1}{2} N^\alpha$, whenever $v,q\in P_L$, $r\in P_H$ and $N\in {\mathbb {N}}$ sufficiently large (otherwise $|v+r+u|\ge \frac{1}{4}N^{\alpha }-N^\beta > N^\beta$ for large enough $N\in {\mathbb {N}}$). Finally, $\zeta _6$ can be controlled by

$$\begin{aligned} \begin{aligned}&|\langle \xi , \zeta _6\xi \rangle | \\&\quad = \bigg | \frac{1}{N }\sum ^*_{\begin{array}{c} r\in P_{H},\\ v,w,q\in P_{L} \end{array}}\int _{\Lambda ^2} N^{2-2\kappa }V(N^{1-\kappa }(x-y)) e^{-ivx-iqy} \eta _r \langle \xi , a^*_{v+r} a^*_{w-r}a^*_q a_w \check{a}_{x} \check{a}_y\xi \rangle \bigg | \\&\quad \le CN^{\beta /2 + \kappa -\alpha /2-1/2} \Vert {\mathcal {V}}_N^{1/2}\xi \Vert \bigg ( N^{\kappa -1}\int _{\Lambda }dx\; \sum ^*_{\begin{array}{c} r\in P_{H},\\ w,q\in P_{L} \end{array}} |q| \Big \Vert \sum _{v\in P_L}e^{-ivx}a_{v+r} a_{w-r}a_q \xi \Big \Vert ^2\bigg )^{1/2}\\&\quad \le CN^{\beta /2 +\kappa /2-1/2} \Vert {\mathcal {V}}_N^{1/2}\xi \Vert \Vert {\mathcal {K}}_L^{1/2}({\mathcal {N}}_{\ge \frac{1}{2} N^\alpha }+1)^{1/2}\xi \Vert \end{aligned}\end{aligned}$$

In summary, the previous estimates show that

7.62 $$\begin{aligned} \begin{aligned} \pm \sum _{j=1}^6 \big ( \zeta _{j}+\text {h.c.}\big )&\le \delta {\mathcal {V}}_N + CN^{3\beta /2 + \kappa /2-1}{\mathcal {V}}_N + CN^{\alpha +\beta +2\kappa -1} {\mathcal {K}}_{\le 2N^\beta } + CN^{2\beta +\kappa } \\&\quad + C(1+\delta ^{-1})N^{\beta +\kappa -1}{\mathcal {K}}_{\le 3N^\beta }({\mathcal {N}}_{\ge \frac{1}{2} N^\alpha }+1) \end{aligned}\end{aligned}$$

for all $\delta >0$. On the other hand, by Lemma 7.3, we also know that

7.63 $$\begin{aligned} \begin{aligned}&\pm \bigg [ \frac{1}{N} \sum ^*_{\begin{array}{c} u\in \Lambda ^*, v, w \in P_L:\\ v+u, w-u\in P_L \end{array}} N^{\kappa } ({\widehat{V}}(./N^{1-\kappa })*\eta /N)(u)\int _0^s dt\; e^{-tD}a^*_{v+u} a^*_{w-u}a_va_w e^{tD} \\&\qquad - \frac{s}{N} \sum ^*_{\begin{array}{c} u\in \Lambda ^*, v, w \in P_L:\\ v+u, w-u\in P_L \end{array}} N^{\kappa } ({\widehat{V}}(./N^{1-\kappa })*\eta /N)(u) a^*_{v+u} a^*_{w-u}a_va_w \bigg ]\\&\quad \le C N^{-3\beta -3\kappa }{\mathcal {K}}+ CN^{3\beta +\kappa -2} + CN^{\beta +\kappa -1}{\mathcal {K}}_L({\mathcal {N}}_{\ge \frac{1}{2}N^\alpha }+1)\, . \end{aligned} \end{aligned}$$

Now, going back to (7.55), the bounds (7.62) and (7.63) imply that

7.64 $$\begin{aligned} \begin{aligned} e^{-sD} \text {W} e^{sD}&= \text {W} + \frac{s}{N} \sum ^*_{\begin{array}{c} u\in \Lambda ^*, v, w \in P_L:\\ v+u, w-u\in P_L \end{array}} N^{\kappa } ({\widehat{V}}(./N^{1-\kappa })*\eta /N)(u) a^*_{v+u} a^*_{w-u}a_va_w\\&\quad + {\mathcal {E}}_1(s)+{\mathcal {E}}_2(s,\delta ), \end{aligned} \end{aligned}$$

where the self-adjoint operators ${\mathcal {E}}_1(s)$ and ${\mathcal {E}}_2(s)$ are bounded by

$$\begin{aligned} \begin{aligned} \pm {\mathcal {E}}_1(s)&\le C(N^{\alpha +\beta +2\kappa -1} +N^{-3\beta -3\kappa }) {\mathcal {K}}+ CN^{2\beta +\kappa }, \end{aligned}\end{aligned}$$

as well as

$$\begin{aligned} \begin{aligned} \pm {\mathcal {E}}_2(s,\delta )&\le CN^{\beta +\kappa -1}{\mathcal {K}}_L({\mathcal {N}}_{\ge \frac{1}{2}N^\alpha }+1) + C(\delta + CN^{3\beta /2 + \kappa /2-1}) \int _0^s dt\; e^{-tD} {\mathcal {V}}_N e^{tD}\\&\quad + C(1+\delta ^{-1})N^{\beta +\kappa -1}\int _0^s dt\; e^{-tD} {\mathcal {K}}_{\le 2N^\beta }({\mathcal {N}}_{\ge \frac{1}{2} N^\alpha }+1)e^{tD}, \end{aligned}\end{aligned}$$

for all $\delta >0$ and uniformly in $s\in [-1;1]$. Defining ${\mathcal {E}}_2(s) = {\mathcal {E}}_2(s, N^{-\beta -\kappa })$, this concludes the proof. $\square$

Equipped with Proposition 7.11, we go back to (7.50) and conclude that

7.65 $$\begin{aligned} \begin{aligned} {\mathcal {M}}_N^{(4)}&\ge {\mathcal {H}}_N - \frac{1}{2N}\sum _{\begin{array}{c} r\in \Lambda ^*, v,w\in P_L: \\ v+r, w-r\ne 0 \end{array}} {\widehat{V}}(r/N^{1-\kappa })\big (a^*_{v+r}a^*_{w-r}a_va_w+\text {h.c.}\big )\\&\quad -\frac{1}{2N}\sum ^*_{\begin{array}{c} u\in \Lambda ^*, v, w \in P_L:\\ v+u, w-u\in P_L \end{array}} N^{\kappa } ({\widehat{V}}(./N^{1-\kappa })*\eta /N)(u) a^*_{v+u} a^*_{w-u}a_va_w \\&\quad - \frac{1}{8}{\mathcal {K}}- CN^{2\beta +\kappa } + \int _0^{1} ds \, {\mathcal {E}}_2(s) + \int _0^1ds\; e^{-sD} \Big ( {\mathcal {E}}_{[{\mathcal {V}}_N, D]} + {\mathcal {E}}_{ [{\mathcal {K}}, D] }\Big ) e^{sD}, \end{aligned} \end{aligned}$$

for all $\alpha \ge 3\beta +2\kappa \ge 0$ with $\alpha +\beta +2\kappa -1<0$, $0\le \kappa <\beta$ and $N\in {\mathbb {N}}$ large enough.

Next, let us analyse the error terms related to ${\mathcal {E}}_2(s)$ and ${\mathcal {E}}_{[{\mathcal {V}}_N, D]}$ further. The bounds (7.53) and (7.21) (with $\delta = c N^{-\beta -\kappa }$ for a sufficiently small $c>0$; this choice guarantees that we can extract the term ${\mathcal {V}}_{N,L}$ in (7.66), with an error that can be absorbed in ${\mathcal {K}}$) imply, together with Lemmas 7.1, 7.2, Corollaries 7.4 and 7.6 and with the assumption (5.6) on the exponents $\alpha , \beta$, that

$$\begin{aligned} \begin{aligned}&\int _0^{1} ds\; \Big (e^D {\mathcal {E}}_2(s)e^{-D} + e^{(1-s)D} {\mathcal {E}}_{[{\mathcal {V}}_N, D]} e^{-(1-s)D} \Big ) \\&\quad \ge - CN^{2\beta +2\kappa -1} {\mathcal {K}}_L({\mathcal {N}}_{\ge \frac{1}{2}N^\alpha }+1) - {\widetilde{C}} N^{-\beta -\kappa } ({\mathcal {V}}_N + {\mathcal {V}}_{N,L}) - CN^{2\beta }({\mathcal {N}}_{\ge \frac{1}{2} N^\alpha }+1) \\&\qquad - CN^{4\beta +2\kappa -1}({\mathcal {N}}_{\ge \frac{1}{2} N^\alpha }+1)^2 \end{aligned}\end{aligned}$$

for all $N\in {\mathbb {N}}$ large enough and for an arbitrarily small constant ${\widetilde{C}} > 0$. With Corollary 7.4 and (7.65), we conclude that

7.66 $$\begin{aligned} \begin{aligned} {\mathcal {M}}_N^{(4)}&\ge {\mathcal {H}}_N - \frac{1}{2N}\sum _{\begin{array}{c} r\in \Lambda ^*, v,w\in P_L: \\ v+r, w-r\ne 0 \end{array}} {\widehat{V}}(r/N^{1-\kappa })\big (a^*_{v+r}a^*_{w-r}a_va_w+\text {h.c.}\big )\\&\quad -\frac{1}{2N}\sum ^*_{\begin{array}{c} u\in \Lambda ^*, v, w \in P_L:\\ v+u, w-u\in P_L \end{array}} N^{\kappa } ({\widehat{V}}(./N^{1-\kappa })*\eta /N)(u) a^*_{v+u} a^*_{w-u}a_va_w \\&\quad - \frac{1}{4}{\mathcal {K}}- CN^{2\beta +\kappa }- {\widetilde{C}} N^{-\beta - \kappa }{\mathcal {V}}_{N,L} + \int _0^1ds\; e^{-sD} {\mathcal {E}}_{ [{\mathcal {K}}, D] } e^{sD} + {\mathcal {E}}_{{\mathcal {M}}_N}^{(41)}, \end{aligned} \end{aligned}$$

where the error ${\mathcal {E}}_{{\mathcal {M}}_N}^{(41)}$ is such that

$$\begin{aligned} \begin{aligned} e^{D} {\mathcal {E}}_{{\mathcal {M}}_N}^{(41)} e^{-D}&\ge - CN^{2\beta +2\kappa -1}{\mathcal {K}}_L({\mathcal {N}}_{\ge \frac{1}{2}N^\alpha }+1) - CN^{-\beta -\kappa } {\mathcal {V}}_N \\&\quad - CN^{2\beta } {\mathcal {N}}_{\ge \frac{1}{2} N^\alpha } - CN^{4\beta +2\kappa -1} {\mathcal {N}}_{\ge \frac{1}{2} N^\alpha }^2 \end{aligned} \end{aligned}$$

Applying Lemmas 4.2, 4.3 and Corollary 4.5, we deduce with the operator inequality ${\mathcal {N}}_{\ge \frac{1}{2} N^\alpha }\le 4N^{-2\alpha }{\mathcal {K}}$ that

7.67 $$\begin{aligned} \begin{aligned} e^A e^{D} {\mathcal {E}}_{{\mathcal {M}}_N}^{(41)} e^{-D} e^{-A}&\ge - CN^{-\beta } {\mathcal {K}}- CN^{-\beta -\kappa } {\mathcal {V}}_N - C N^{2\beta +2\kappa -1} \\&\quad - CN^{2\beta +2\kappa -1}{\mathcal {K}}{\mathcal {N}}_{\ge \frac{1}{2}N^\alpha } \end{aligned} \end{aligned}$$

for all $N\in {\mathbb {N}}$ large enough.

Now, we switch to the contribution containing the operator ${\mathcal {E}}_{ [{\mathcal {K}}, D] }$ on the r.h.s. of the lower bound (7.66). We recall once again that

$$\begin{aligned} \int _0^1ds\; e^{-sD} {\mathcal {E}}_{[{\mathcal {K}},D]} e^{sD} = \int _0^1ds\; \sum _{j=1}^3 e^{-sD}\big (\Sigma _j+\text {h.c.}\big )e^{sD}, \end{aligned}$$

where the operators $\Sigma _1, \Sigma _2$ and $\Sigma _3$ were defined in (7.26). It turns out that $\Sigma _2$ and $\Sigma _3$ are negligible errors while $\Sigma _1$ still contains an important contribution of leading order. We start with the analysis of the contribution related to $\Sigma _1$.

##### Proposition 7.12

Assume the exponents $\alpha , \beta$ satisfy (5.6). Then, we have that

7.68 $$\begin{aligned} \begin{aligned}&\frac{1}{2N}\sum _{\begin{array}{c} u\in P_H^c\cup \{0\}, p, q\in P_L: \\ p+u, q-u\ne 0 \end{array}} N^\kappa \big ({\widehat{V}}(/N^{1-\kappa }) *{\widehat{f}}_N\big )(u) e^{-sD}\big (a^*_{p+u}a^*_{q-u}a_p a_q+h.c. \big )e^{sD} \\&\quad = \frac{1}{2N}\sum _{\begin{array}{c} u\in P_H^c\cup \{0\}, p, q\in P_L: \\ p+u, q-u\ne 0 \end{array}} N^\kappa \big ({\widehat{V}}(/N^{1-\kappa })*{\widehat{f}}_N\big )(u) \big (a^*_{p+u}a^*_{q-u}a_p a_q+h.c. \big ) + {\mathcal {E}}_3(s) \end{aligned} \end{aligned}$$

and there exists a constant $C>0$ such that

7.69 $$\begin{aligned} \begin{aligned}&\pm e^{A}e^D{\mathcal {E}}_3(s) e^{-D}e^{-A} \\&\quad \le C N^{\alpha + \beta + 2 \kappa -1} {\mathcal {K}}+ C N^{\alpha + \beta + 2 \kappa -1} {\mathcal {K}}{\mathcal {N}}_{\ge \frac{1}{2} N^\alpha } +CN^{4\beta +2\kappa } + C N^{\alpha + 3\beta + 2 \kappa -1} \end{aligned} \end{aligned}$$

for all $s\in [-1;1]$ and for all $N\in {\mathbb {N}}$ sufficiently large.

##### Proof

We proceed as in Proposition 7.11 and recall $\Sigma _1:{\mathcal {F}}_+^{\le N}\rightarrow {\mathcal {F}}_+^{\le N}$ to be

$$\begin{aligned} \begin{aligned}&\Sigma _{1} = \frac{1}{2N}\sum _{\begin{array}{c} u\in P_H^c\cup \{0\}, p, q\in P_L: \\ p+u, q-u\ne 0 \end{array}} N^\kappa \big ({\widehat{V}}(/N^{1-\kappa }) *{\widehat{f}}_N\big )(u) \big (a^*_{p+u}a^*_{q-u}a_p a_q+\text {h.c.}\big ). \end{aligned}\end{aligned}$$

We then have

7.70 $$\begin{aligned} \begin{aligned}&e^{-sD}\Sigma _{1} e^{sD} - \Sigma _{1} = \int _0^sdt \; e^{-tD} [ \Sigma _{1}, D_1] e^{tD} + \text {h.c.} \end{aligned} \end{aligned}$$

Similarly as in (7.58) and (7.2.3), we find that

7.71 $$\begin{aligned} {[}\Sigma _{1}, D_1] +\text {h.c.} = \sum _{i=1}^8 (\Gamma _i +\text {h.c.}), \end{aligned}$$

where

$$\begin{aligned} \Gamma _{1}&= \frac{1}{N^2} \sum ^*_{\begin{array}{c} u\in P_H^c\cup \{0\}, r\in P_H, v, w \in P_L:\\ v+u+r, w-u-r\in P_L \end{array}} N^{\kappa } \big ({\widehat{V}}( . /N^{1-\kappa }) *{\widehat{f}}_N\big )(u) \eta _r a^*_{v+u+r} a^*_{w-u-r}a_va_w,\\ \Gamma _{2}&= -\frac{1}{2N^{2}}\sum ^*_{\begin{array}{c} u\in P_H^c\cup \{0\}, r\in P_{H},\\ v,w\in P_{L}:\\ w-u, v+u\in P_L \end{array}}N^\kappa \big ({\widehat{V}}(. /N^{1-\kappa }) *{\widehat{f}}_N\big )(u) \eta _r a^*_{v+r} a^*_{w-r} a_{w-u} a_{v+u} ,\\ \Gamma _{3}&= -\frac{1}{2N^{2}}\sum ^*_{\begin{array}{c} u\in P_H^c\cup \{0\}, r\in P_{H},\\ v,w\in P_{L} \end{array}}N^\kappa \big ({\widehat{V}}(. /N^{1-\kappa }) *{\widehat{f}}_N\big )(u)\eta _r a^*_{v+r} a^*_{w-r} a_{w-u} a_{v+u} ,\\ \Gamma _{4}&= -\frac{1}{N^{2}}\sum ^*_{\begin{array}{c} u\in P_H^c\cup \{0\}, r\in P_{H},\\ v,w,q\in P_L:\\ v-u\in P_{L} \end{array}}N^\kappa \big ({\widehat{V}}(. /N^{1-\kappa }) *{\widehat{f}}_N\big )(u) \eta _r a^*_{v+r} a^*_{w-r}a^*_{q-u} a_w a_{v-u} a_{q},\\ \Gamma _{5}&= \frac{1}{N^{2}}\sum ^*_{\begin{array}{c} u\in P_H^c\cup \{0\}, r\in P_{H},\\ v,w,q \in P_L:\\ v+r+u\in P_{L} \end{array}}N^\kappa \big ({\widehat{V}}(. /N^{1-\kappa }) *{\widehat{f}}_N\big )(u) \eta _r a^*_{v+r+u}a^*_q a^*_{w-r}a_{q+u} a_v a_w ,\\ \Gamma _{6}&= -\frac{1}{N^{2}}\sum ^*_{\begin{array}{c} u\in P_H^c\cup \{0\}, r\in P_{H},\\ v,w,q\in P_{L} \end{array}}N^\kappa \big ({\widehat{V}}(. /N^{1-\kappa }) *{\widehat{f}}_N\big )(u) \eta _r a^*_{v+r} a^*_{w-r}a^*_q a_w a_{v-u} a_{q+u}. \end{aligned}$$

The operators $\Gamma _1$ to $\Gamma _6$ can be bounded similarly as in the proof of Proposition 7.11. Let us start with $\Gamma _1$. Applying as usual Cauchy-Schwarz implies that

$$\begin{aligned}\begin{aligned}&|\langle \xi , \Gamma _1\xi \rangle | \\&\quad \le \frac{CN^\kappa }{N^2} \sum ^*_{\begin{array}{c} u\in P_H^c\cup \{0\}, r\in P_H, v, w \in P_L:\\ v+u+r, w-u-r\in P_L \end{array}} \bigg ( |v|^{-1} \Vert a_{v+u+r} a_{w-u-r}\xi \Vert \bigg ) \bigg ( |\eta _r||v| \Vert a_va_w\xi \Vert \bigg ) \\&\quad \le CN^{\alpha /2+ 5\beta /2+ 2\kappa -1/2} \Vert \xi \Vert \Vert {\mathcal {K}}_L^{1/2}\xi \Vert \le CN^{\alpha + \beta + 2\kappa -1}\langle \xi , {\mathcal {K}}_L\xi \rangle + CN^{4\beta +2\kappa } \Vert \xi \Vert ^2 \end{aligned} \end{aligned}$$

where we used that $v+u+r \in P_L$ implies $|u|\ge N^{\alpha }-3N^\beta$ and $|r|\le N^{\alpha }+3N^\beta$ whenever $u\in P_H^c, r\in P_H$ and $v\in P_L$ (otherwise $|u+r+v| \ge |r| - |u|-N^\beta \ge 2N^\beta > N^\beta$ if either $|u|\le N^{\alpha }-3N^\beta$ or $|r|\ge N^{\alpha }+3N^\beta$, in contradiction to $u+r+v\in P_L$) for $N\in {\mathbb {N}}$ sufficiently large. Notice in addition that $\sum _{ N^\alpha -3N^\beta \le |u|\le N^\alpha } \le C N^{2\alpha +\beta }$.

The term $\Gamma _2$ can be estimated exactly as the term $\zeta _2$ in (7.60), that is

$$\begin{aligned} |\langle \xi , \Gamma _2\xi \rangle | \le CN^{\alpha +\beta +2\kappa -1} \langle \xi , {\mathcal {K}}_{\le 2N^\beta }\xi \rangle + CN^{2\beta +\kappa -1}\langle \xi , ({\mathcal {N}}_{\ge \frac{1}{2}N^\alpha }+1)\xi \rangle . \end{aligned}$$

The contribution $\Gamma _3$ can be controlled by

$$\begin{aligned}\begin{aligned} |\langle \xi , \Gamma _3\xi \rangle |&\le \frac{CN^\kappa }{2N^{2}}\!\!\!\!\sum ^*_{\begin{array}{c} u\in P_H^c\cup \{0\}, r\in P_{H},\\ v,w\in P_{L} \end{array}}\!\!\!\!|\eta _r| \Vert a_{v+r}({\mathcal {N}}_{\ge \frac{1}{2}N^\alpha }+1)^{-1/2} a_{w-r}\xi \Vert \\&\quad \Vert a_{w-u} ({\mathcal {N}}_{\ge \frac{1}{2}N^\alpha }+1)^{1/2}a_{v+u} \xi \Vert \\&\le CN^{\alpha + 3\beta + 2\kappa -1} \langle \xi , ({\mathcal {N}}_{\ge \frac{1}{2}N^\alpha }+1)\xi \rangle . \end{aligned} \end{aligned}$$

The terms $\Gamma _4$ and $\Gamma _5$ can be bounded exactly as in (7.61). We find

$$\begin{aligned} |\langle \xi , \Gamma _4\xi \rangle | +|\langle \xi , \Gamma _5\xi \rangle | \le C N^{\beta +\kappa -1} \langle \xi , {\mathcal {K}}_{\le 2N^\beta }({\mathcal {N}}_{\ge \frac{1}{2} N^\alpha }+1)\xi \rangle , \end{aligned}$$

Finally, the last contribution $\Gamma _6$ is bounded by

$$\begin{aligned}\begin{aligned} |\langle \xi , \Gamma _6\xi \rangle |&\le \frac{CN^\kappa }{N^{2}}\sum ^*_{\begin{array}{c} u\in P_H^c\cup \{0\}, r\in P_{H},\\ v,w,q\in P_{L} \end{array}} \Big ( |q||w|^{-1} \Vert a_{v+r} ({\mathcal {N}}_{\ge \frac{1}{2}N^\alpha }+1)^{-1/2}a_{w-r}a _q \xi \Vert \Big )\\&\quad \times \Big ( |\eta _r||w| |q|^{-1}\Vert a_{v-u} ({\mathcal {N}}_{\ge \frac{1}{2}N^\alpha }+1)^{1/2} a_wa_{q+u} \xi \Vert \Big )\\&\le C N^{\alpha + \beta + 2 \kappa -1} \langle \xi , {\mathcal {K}}_L({\mathcal {N}}_{\ge \frac{1}{2} N^\alpha }+1)\xi \rangle . \end{aligned}\end{aligned}$$

In conclusion, the above estimates imply that

$$\begin{aligned} \begin{aligned} \pm \sum _{i=1}^6 \big ( \Gamma _i+\text {h.c.}\big )&\le C N^{\alpha + \beta + 2 \kappa -1} {\mathcal {K}}_{\le 2N^\beta }+ C N^{\alpha + \beta + 2 \kappa -1} {\mathcal {K}}_{\le 2N^\beta }({\mathcal {N}}_{\ge \frac{1}{2} N^\alpha }+1)\\&\quad + CN^{\alpha + 3\beta + 2\kappa -1} ({\mathcal {N}}_{\ge \frac{1}{2}N^\alpha }+1)+CN^{4\beta +2\kappa } \end{aligned}\end{aligned}$$

for all $\alpha >3\beta +2\kappa \ge 0$ and for all $N\in {\mathbb {N}}$ sufficiently large. Combining this estimate with the identites (7.70) and (7.71), and applying Lemmas 4.2, 4.3, 7.1 as well as Lemma 7.2 together with the operator inequality ${\mathcal {N}}_{\ge \frac{1}{2} N^\alpha }\le 4 N^{-2\alpha }{\mathcal {K}}$ proves the proposition. $\square$

Applying Proposition 7.12 to the lower bound (7.66) and defining ${\mathcal {E}}_{{\mathcal {M}}_N}^{(42)} = \int _0^1 ds\; {\mathcal {E}}_3(s)$ with ${\mathcal {E}}(s)$ from Proposition 7.12, we conclude that

7.72 $$\begin{aligned} \begin{aligned} {\mathcal {M}}_N^{(4)}&\ge {\mathcal {H}}_N - \frac{1}{2N}\sum _{\begin{array}{c} r\in \Lambda ^*, v,w\in P_L: \\ v+r, w-r\ne 0 \end{array}} {\widehat{V}}(r/N^{1-\kappa })\big (a^*_{v+r}a^*_{w-r}a_va_w+\text {h.c.}\big )\\&\quad -\frac{1}{2N}\sum ^*_{\begin{array}{c} u\in \Lambda ^*, v, w \in P_L:\\ v+u, w-u\in P_L \end{array}} N^{\kappa } ({\widehat{V}}(./N^{1-\kappa })*\eta /N)(u) a^*_{v+u} a^*_{w-u}a_va_w \\&\quad +\frac{1}{2N}\sum _{\begin{array}{c} u\in P_H^c\cup \{0\}, p, q\in P_L: \\ p+u, q-u\ne 0 \end{array}} N^\kappa \big ({\widehat{V}}(/N^{1-\kappa })*{\widehat{f}}_N\big )(u) \big (a^*_{p+u}a^*_{q-u}a_p a_q+h.c. \big )\\&\quad - \frac{1}{4}{\mathcal {K}}- C N^{-\beta -\kappa }{\mathcal {V}}_{N,L} + {\mathcal {E}}_{{\mathcal {M}}_N}^{(41)}+ {\mathcal {E}}_{{\mathcal {M}}_N}^{(42)} + \int _0^1ds\; e^{-sD} \big ( \Sigma _2+\Sigma _3+\text {h.c.}\big ) e^{sD}, \end{aligned} \end{aligned}$$

where ${\mathcal {E}}_{{\mathcal {M}}_N}^{(41)}$ satisfies the lower bound (7.67), ${\mathcal {E}}_{{\mathcal {M}}_N}^{(42)}$ satisfies the bound (7.69) and where the operators $\Sigma _2$ and $\Sigma _3$ were defined in (7.26).

Let us finally estimate the size of the error in the last line of (7.72), involving the two operators $\Sigma _2$ and $\Sigma _3$. Using the estimate (7.31) together with Lemmas 4.2, 4.3, 7.1 and 7.2, we find for ${\mathcal {E}}_{{\mathcal {M}}_N}^{(43)} = \int _0^1ds\; e^{-sD} \big ( \Sigma _2+\text {h.c.}\big ) e^{sD}$

7.73 $$\begin{aligned} \begin{aligned} e^{A}e^{D}{\mathcal {E}}_{{\mathcal {M}}_N}^{(43)}e^{-D}e^{-A} \ge - CN^{-\beta -1} {\mathcal {K}}{\mathcal {N}}_{\ge \frac{1}{2} N^\alpha }-CN^{-5\beta -4\kappa }{\mathcal {K}}-CN^\beta . \end{aligned} \end{aligned}$$

Finally, consider the operator ${\mathcal {E}}_{{\mathcal {M}}_N}^{(44)} = \int _0^1ds\; e^{-sD} \big ( \Sigma _3+\text {h.c.}\big ) e^{sD}$, with $\Sigma _3$ defined in (7.26). Let $m_0\in {\mathbb {R}}$ be such that $m_0\beta =\alpha$ (in particular, $\lfloor m_0 \rfloor \ge 3$). Here, we use the bound (7.32) to find first of all that

$$\begin{aligned} \begin{aligned} {\mathcal {E}}_{{\mathcal {M}}_N}^{(44)} \ge&- \int _0^1 ds\; \Vert {\mathcal {K}}^{1/2}e^{sD} \xi \Vert \Big ( N^{-1/2}\big \Vert {\mathcal {K}}_L^{1/2}({\mathcal {N}}_{\ge \frac{1}{2} N^\alpha }+1)^{1/2}\xi \big \Vert \\&+ N^{\beta -1}\big \Vert ({\mathcal {N}}_{\ge \frac{1}{2} N^\alpha }+1)^{3/2}\xi \big \Vert \Big ) \end{aligned} \end{aligned}$$

for any $\xi \in {\mathcal {F}}_+^{\le N}$ with $\Vert \xi \Vert =1$. Notice that we applied once again Lemmas 7.1 and 7.2 in the second factor. With Corollary 7.8, the first factor is bounded by

$$\begin{aligned}\begin{aligned}&{\mathcal {E}}_{{\mathcal {M}}_N}^{(44)} \\&\quad \ge - C \bigg ( \Vert {\mathcal {K}}^{1/2}\xi \Vert + \Vert {\mathcal {V}}_N^{1/2}\xi \Vert + \Vert {\mathcal {V}}_{N,L}^{1/2}\xi \Vert + N^{5\beta /8+\kappa /2}\Vert {\mathcal {K}}_{\le N^{3\beta /2}}^{1/2} \xi \Vert \\&\qquad +N^{-1/2}\big \Vert {\mathcal {K}}_L^{1/2}({\mathcal {N}}_{\ge \frac{1}{2} N^\alpha }+1)^{1/2}\xi \big \Vert + N^{3\beta /2+\kappa /2} \\&\qquad + \sum _{j=3}^{2\lfloor m_0\rfloor -1}N^{ j\beta /4 + 3\beta /4+\kappa -1/2}\Big [ \big \Vert {\mathcal {K}}_L^{1/2}({\mathcal {N}}_{\ge \frac{1}{2} N^{j\beta /2}}+1)^{1/2}\xi \big \Vert \\&\qquad + N^\beta \big \Vert ({\mathcal {N}}_{\ge \frac{1}{2} N^{\alpha }}+1)^{1/2}({\mathcal {N}}_{\ge \frac{1}{2} N^{j\beta /2}}+1)^{1/2}\xi \big \Vert \Big ] \\&\qquad + N^{ \alpha /2 +\beta /2 +\kappa -1/2}\Big [ \big \Vert {\mathcal {K}}_L^{1/2}({\mathcal {N}}_{\ge \frac{1}{2} N^{\lfloor m_0 \rfloor \beta }} +1)^{1/2}\xi \big \Vert \\&\qquad + N^\beta \big \Vert ({\mathcal {N}}_{\ge \frac{1}{2} N^{\alpha }}+1)^{1/2}({\mathcal {N}}_{\ge \frac{1}{2} N^{\lfloor m_0 \rfloor \beta }} +1)^{1/2}\xi \big \Vert \Big ] \bigg ) \\&\qquad \times \bigg ( N^{-1/2}\big \Vert {\mathcal {K}}_L^{1/2}({\mathcal {N}}_{\ge \frac{1}{2} N^\alpha }+1)^{1/2}\xi \big \Vert + N^{\beta -1}\big \Vert ({\mathcal {N}}_{\ge \frac{1}{2} N^\alpha }+1)^{3/2}\xi \big \Vert \bigg ) \end{aligned}\end{aligned}$$

for all exponents $\alpha , \beta$ satisfying (5.6) and $N\in {\mathbb {N}}$ sufficiently large. It follows that

7.74 $$\begin{aligned} \begin{aligned} {\mathcal {E}}_{{\mathcal {M}}_N}^{(44)} \ge {\mathcal {E}}_{{\mathcal {M}}_N}^{(441)} +{\mathcal {E}}_{{\mathcal {M}}_N}^{(442)} +{\mathcal {E}}_{{\mathcal {M}}_N}^{(443)}, \end{aligned}\end{aligned}$$

where

7.75 $$\begin{aligned} \begin{aligned} {\mathcal {E}}_{{\mathcal {M}}_N}^{(441)} = - \frac{1}{8} {\mathcal {K}}- {\widetilde{C}} N^{-\alpha } {\mathcal {V}}_{N,L}-CN^{3\beta +\kappa },  {\mathcal {E}}_{{\mathcal {M}}_N}^{(442)} = N^{-\alpha }{\mathcal {V}}_N \end{aligned} \end{aligned}$$

with an arbitrarily small constant ${\widetilde{C}} >0$ and where after an additional application of Lemmas 4.2, 4.3, 7.1 and 7.2 together with the operator bound ${\mathcal {N}}_{\ge \Theta }\le \Theta ^{-2}{\mathcal {K}}$, the error ${\mathcal {E}}_{{\mathcal {M}}_N}^{(443)}$ is such that

7.76 $$\begin{aligned} \begin{aligned}&e^A e^{D} {\mathcal {E}}_{{\mathcal {M}}_N}^{(443)}e^{-D}e^{-A}\\&\quad \ge -C N^{\alpha +\beta +2\kappa -1}{\mathcal {K}}-C N^{\alpha -1}{\mathcal {K}}{\mathcal {N}}_{\ge \frac{1}{2}N^\alpha } - C N^{\alpha +3\beta +2\kappa -1}\\&\qquad - C \sum _{j=3}^{2\lfloor m_0\rfloor -1}N^{ j\beta /2 + \beta /2+2\kappa -1}{\mathcal {K}}{\mathcal {N}}_{\ge \frac{1}{2} N^{j\beta /2}}-C N^{ \alpha +\beta +2\kappa -1}{\mathcal {K}}{\mathcal {N}}_{\ge \frac{1}{2} N^{\lfloor m_0 \rfloor \beta }} \end{aligned} \end{aligned}$$

for all exponents $\alpha , \beta$ satisfying (5.6) and $N\in {\mathbb {N}}$ sufficiently large.

Choosing ${{\widetilde{C}}}>0$ sufficiently large (but independently of $N\in {\mathbb {N}}$) and arguing as right before (7.66), we deduce that

7.77 $$\begin{aligned} \begin{aligned}&e^A \Big ( {{\widetilde{C}}} N^{-\alpha } e^D{\mathcal {V}}_{N,L } e^{-D}+ e^D {\mathcal {E}}_{{\mathcal {M}}_N}^{(442)} e^{-D}\Big )e^{-A} \\&\quad \ge - C N^{-\alpha }{\mathcal {V}}_N - CN^{-3\beta -\kappa }{\mathcal {N}}_+- CN^{-2\beta -\kappa -1}{\mathcal {K}}{\mathcal {N}}_{\ge \frac{1}{2}N^\alpha } \end{aligned} \end{aligned}$$

for all $\alpha , \beta$ satisfying (5.6) and $N\in {\mathbb {N}}$ sufficiently large. This follows through another application of Corollaries 4.5, 7.4 and 7.6, together with Lemmas 4.2, 4.3, 7.1 and 7.2. We summarize these bounds in the following corollary.

##### Corollary 7.13

Let $m_0\in {\mathbb {R}}$ be such that $m_0\beta =\alpha$ and let ${\mathcal {M}}_N^{(4)}$ be defined as in (7.35). For every ${\widetilde{C}} > 0$, there exists a constant $C>0$ such that

7.78 $$\begin{aligned} \begin{aligned} {\mathcal {M}}_N^{(4)}&\ge \frac{1}{2}{\mathcal {K}}+{\mathcal {V}}_N - \frac{1}{2N}\sum _{\begin{array}{c} r\in \Lambda ^*, v,w\in P_L: \\ v+r, w-r\ne 0 \end{array}} {\widehat{V}}(r/N^{1-\kappa })\big (a^*_{v+r}a^*_{w-r}a_va_w+\text {h.c.}\big )\\&\quad -\frac{1}{2N}\sum ^*_{\begin{array}{c} u\in \Lambda ^*, v, w \in P_L:\\ v+u, w-u\in P_L \end{array}} N^{\kappa } ({\widehat{V}}(./N^{1-\kappa })*\eta /N)(u) a^*_{v+u} a^*_{w-u}a_va_w \\&\quad +\frac{1}{2N}\sum _{\begin{array}{c} u\in P_H^c\cup \{0\}, p, q\in P_L: \\ p+u, q-u\ne 0 \end{array}} N^\kappa \big ({\widehat{V}}(/N^{1-\kappa })*{\widehat{f}}_N\big )(u) \big (a^*_{p+u}a^*_{q-u}a_p a_q+h.c. \big )\\&\quad -{\widetilde{C}} N^{-\beta -\kappa } {\mathcal {V}}_{N,L} + {\mathcal {E}}_{{\mathcal {M}}_N}^{(4)} \end{aligned} \end{aligned}$$

where

7.79 $$\begin{aligned} \begin{aligned}&e^Ae^D {\mathcal {E}}_{{\mathcal {M}}_N}^{(4)} e^{-D}e^{-A}\\&\quad \ge - C N^{-\beta }{\mathcal {K}}- C N^{-\beta -\kappa } {\mathcal {V}}_N -C N^{ \alpha +\beta +2\kappa -1}{\mathcal {K}}{\mathcal {N}}_{\ge \frac{1}{2} N^{\lfloor m_0 \rfloor \beta }} \\&\qquad - C \sum _{j=3}^{2\lfloor m_0\rfloor -1}N^{ j\beta /2 + \beta /2+2\kappa -1}{\mathcal {K}}{\mathcal {N}}_{\ge \frac{1}{2} N^{j\beta /2}}- CN^{2\beta +\kappa } \end{aligned} \end{aligned}$$

for all exponents $\alpha ,\beta$ satisfying (5.6) and for all $N\in {\mathbb {N}}$ sufficiently large.

##### Proof

The proof follows from defining ${\mathcal {E}}_{{\mathcal {M}}_N}^{(4)} = \sum _{j=1}^3{\mathcal {E}}_{{\mathcal {M}}_N}^{(4j)} + \sum _{j=1}^3{\mathcal {E}}_{{\mathcal {M}}_N}^{(44j)}$ and combining (7.67), (7.72), (7.69), (7.73), (7.74), (7.75), (7.77), (7.76) and the operator bound ${\mathcal {N}}_+\le (2\pi )^{-2}{\mathcal {K}}$ in ${\mathcal {F}}_+^{\le N}$. $\square$

### Proof of Proposition 5.1

Recall from (7.34) the decomposition

$$\begin{aligned} {\mathcal {M}}_N = 4\pi {\mathfrak {a}}_0N^{1+\kappa }-4\pi {\mathfrak {a}}_0N^{\kappa -1}{\mathcal {N}}_+^{\,2}/N + {\mathcal {M}}_N^{(2)}+ {\mathcal {M}}_N^{(3)} + {\mathcal {M}}_N^{(4)} \end{aligned}$$

Collecting the results of Propositions 7.9, 7.10 and Corollary 7.13, we deduce that

7.80 $$\begin{aligned} {\mathcal {M}}_N&\ge 4\pi {\mathfrak {a}}_0N^{1+\kappa }-4\pi {\mathfrak {a}}_0N^{\kappa -1}{\mathcal {N}}_+^{\,2} + 8\pi {\mathfrak {a}}_0N^\kappa \sum _{p\in P_H^c}\Big [ b^*_pb_p +\frac{1}{2} b^*_pb^*_{-p} + \frac{1}{2} b_pb_{-p}\Big ]\nonumber \\&\quad + \frac{8\pi {\mathfrak {a}}_0N^\kappa }{\sqrt{N}}\sum _{\begin{array}{c} p\in P_H^c,q\in P_L:\nonumber \\ p+q\ne 0 \end{array}} \big [ b^*_{-p}a^*_{p+q}a_q +\text {h.c.}\big ]+\frac{1}{2}{\mathcal {K}}\nonumber \\&\quad +{\mathcal {V}}_N - \frac{1}{2N}\sum _{\begin{array}{c} r\in \Lambda ^*, v,w\in P_L: \nonumber \\ v+r, w-r\ne 0 \end{array}} {\widehat{V}}(r/N^{1-\kappa })\big (a^*_{v+r}a^*_{w-r}a_va_w+\text {h.c.}\big )\nonumber \\&\quad -\frac{1}{2N}\sum ^*_{\begin{array}{c} r\in \Lambda ^*, v, w \in P_L:\nonumber \\ v+r, w-r\in P_L \end{array}} N^{\kappa } ({\widehat{V}}(./N^{1-\kappa })*\eta /N)(r) a^*_{v+r} a^*_{w-r}a_va_w \nonumber \\&\quad +\frac{1}{2N}\sum _{\begin{array}{c} r\in P_H^c\cup \{0\}, v, w\in P_L: \nonumber \\ v+r,w-r\ne 0 \end{array}} N^\kappa \big ({\widehat{V}}(./N^{1-\kappa })*{\widehat{f}}_N\big )(r) \big (a^*_{v+r}a^*_{w-r}a_v a_w+\text {h.c.}\big )\nonumber \\&\quad - {\widetilde{C}} N^{-\beta -\kappa }{\mathcal {V}}_{N,L} +{\mathcal {E}}'_{{\mathcal {M}}_N}, \end{aligned}$$

where ${\mathcal {E}}'_{{\mathcal {M}}_N}$ satisfies the lower bound

7.81 $$\begin{aligned} \begin{aligned} e^Ae^D {\mathcal {E}}'_{{\mathcal {M}}_N} e^{-D}e^{-A}&\ge - C N^{-\beta } {\mathcal {K}}- C N^{-\beta -\kappa }{\mathcal {V}}_N -C N^{ \alpha +\beta +2\kappa -1}{\mathcal {K}}{\mathcal {N}}_{\ge \frac{1}{2} N^{\lfloor m_0 \rfloor \beta }} \\&\quad - C \sum _{j=3}^{2\lfloor m_0\rfloor -1}N^{ j\beta /2 + \beta /2+2\kappa -1}{\mathcal {K}}{\mathcal {N}}_{\ge \frac{1}{2} N^{j\beta /2}}- CN^{\alpha + \beta /2 +2\kappa } \end{aligned} \end{aligned}$$

for all $N\in {\mathbb {N}}$ sufficiently large.

We combine next the terms on the third, fourth and fifth lines in (7.3). We first notice that

7.82 $$\begin{aligned}&\frac{1}{2N}\sum _{\begin{array}{c} r\in \Lambda ^*, v,w\in P_L: \nonumber \\ v+r, w-r\ne 0 \end{array}} {\widehat{V}}(r/N^{1-\kappa })\big (a^*_{v+r}a^*_{w-r}a_va_w+a^*_{v}a^*_{w}a_{w-r}a_{v+r}\big ) \nonumber \\&\quad = \frac{1}{2N}\sum _{\begin{array}{c} r\in \Lambda ^*, v,w\in \Lambda _+^* : \nonumber \\ v,w\in P_L, \nonumber \\ v+r, w-r\ne 0 \end{array}}\!\!\! {\widehat{V}}(r/N^{1-\kappa })a^*_{v+r}a^*_{w-r}a_va_w \nonumber \\&\qquad + \frac{1}{2N}\!\!\sum _{\begin{array}{c} r\in \Lambda ^*, v,w\in \Lambda _+^* : \nonumber \\ v+r,w-r\in P_L \end{array}} \!\!\!\!\! {\widehat{V}}(r/N^{1-\kappa })a^*_{v+r}a^*_{w-r}a_va_w \nonumber \\&\quad = \frac{1}{2N}\sum ^*_{\begin{array}{c} r\in \Lambda ^*, v,w\in \Lambda _+^* : \nonumber \\ (v,w)\in P_L^2\text { or }( v+r, w-r)\in P_L^2 \end{array}} {\widehat{V}}(r/N^{1-\kappa })a^*_{v+r}a^*_{w-r}a_va_w \nonumber \\&\qquad + \frac{1}{2N}\sum ^*_{\begin{array}{c} r\in \Lambda ^*, v,w\in \Lambda _+^* : \nonumber \\ (v,w, v+r, w-r)\in P_L^4 \end{array}} {\widehat{V}}(r/N^{1-\kappa })a^*_{v+r}a^*_{w-r}a_va_w\nonumber \\ \end{aligned}$$

Arguing in the same way for the contribution on the fifth line in (7.3), using that $({\widehat{f}}_N-\eta /N)(p)=\delta _{p,0}$ for all $p\in \Lambda _+^*$, and using that $v\in P_L$ and $v+r\in P_L$ implies in particular that $r\in P_H^c$, we therefore obtain that

7.83 $$\begin{aligned}&{\mathcal {V}}_N - \frac{1}{2N}\sum ^*_{\begin{array}{c} r\in \Lambda ^*, v,w\in \Lambda _+^*: \nonumber \\ (v,w)\in P_L^2\text { or }(v+r, w-r)\in P_L^2 \end{array}} {\widehat{V}}(r/N^{1-\kappa }) a^*_{v+r}a^*_{w-r}a_va_w \nonumber \\&\qquad - \frac{1}{2N}\sum _{\begin{array}{c} r\in \Lambda ^*, v,w\in \Lambda _+^*: \nonumber \\ (v,w,v+r, w-r)\in P_L^4 \end{array}} {\widehat{V}}(r/N^{1-\kappa }) a^*_{v+r}a^*_{w-r}a_va_w \nonumber \\&\qquad -\frac{1}{2N}\sum ^*_{\begin{array}{c} r\in \Lambda ^*, v, w \in P_L: \nonumber \\ v+r, w-r\in P_L \end{array}} N^{\kappa } ({\widehat{V}}(./N^{1-\kappa })*\eta /N)(r) a^*_{v+r} a^*_{w-r}a_va_w \nonumber \\&\qquad +\frac{1}{2N}\sum _{\begin{array}{c} r\in P_H^c\cup \{0\}, v, w\in P_L: \nonumber \\ v+r, w-r\ne 0 \end{array}} N^\kappa \big ({\widehat{V}}(/N^{1-\kappa })*{\widehat{f}}_N\big )(r) \big (a^*_{v+r}a^*_{w-r}a_v a_w+\text {h.c.}\big ) \nonumber \\&\quad = {\mathcal {V}}_N - \frac{1}{2N}\sum ^*_{\begin{array}{c} r\in \Lambda ^*, v,w\in \Lambda _+^*: \nonumber \\ (v,w)\in P_L^2\text { or }(v+r, w-r)\in P_L^2 \end{array}} {\widehat{V}}(r/N^{1-\kappa }) a^*_{v+r}a^*_{w-r}a_va_w \nonumber \\&\qquad +\frac{1}{2N}\sum _{\begin{array}{c} r\in P_H^c\cup \{0\}, v, w\in P_L: \nonumber \\ (v,w)\in P_L^2\text { or }(v+r, w-r)\in P_L^2 \end{array}}^* N^\kappa \big ({\widehat{V}}(/N^{1-\kappa })*{\widehat{f}}_N\big )(r) a^*_{v+r}a^*_{w-r}a_v a_w.\nonumber \\ \end{aligned}$$

Now, notice furthermore that

$$\begin{aligned}&{\mathcal {V}}_N - \frac{1}{2N}\sum ^*_{\begin{array}{c} r\in \Lambda ^*, v,w\in \Lambda _+^*: \\ (v,w)\in P_L^2\text { or }(v+r, w-r)\in P_L^2 \end{array}} {\widehat{V}}(r/N^{1-\kappa }) a^*_{v+r}a^*_{w-r}a_va_w\\&\quad =\frac{1}{2N}\sum ^*_{\begin{array}{c} r\in \Lambda ^*, v,w\in \Lambda _+^*: \\ (v,w)\in (P_L^2)^c \text { and }\\ (v+r, w-r)\in (P_L^2)^c \end{array}} {\widehat{V}}(r/N^{1-\kappa }) a^*_{v+r}a^*_{w-r}a_va_w, \end{aligned}$$

such that, after switching to position space, the pointwise positivity $V\ge 0$ implies

7.84 $$\begin{aligned} \begin{aligned}&{\mathcal {V}}_N - \frac{1}{2N}\sum ^*_{\begin{array}{c} r\in \Lambda ^*, v,w\in \Lambda _+^*: \\ (v,w)\in P_L^2\text { or }(v+r, w-r)\in P_L^2 \end{array}} {\widehat{V}}(r/N^{1-\kappa }) a^*_{v+r}a^*_{w-r}a_va_w\\&\quad = \int _{\Lambda ^2}dxdy\; N^{2-2\kappa }V (N^{1-\kappa }(x-y))\\&\qquad \times \Big [ a^*\big ( (\check{\chi }_{P_L^c})_x\big )a^*\big ( (\check{\chi }_{P_L^c})_y\big ) + a^*\big ( (\check{\chi }_{P_L})_x\big ) a^*\big ( (\check{\chi }_{P_L^c})_y\big ) + a^*\big ( (\check{\chi }_{P_L^c})_x\big )a^*\big ( (\check{\chi }_{P_L})_y\big )\Big ] \\&\qquad \times \Big [ a\big ( (\check{\chi }_{P_L^c})_x\big )a\big ( (\check{\chi }_{P_L^c})_y\big ) + a\big ( (\check{\chi }_{P_L})_x\big ) a\big ( (\check{\chi }_{P_L^c})_y\big ) + a\big ( (\check{\chi }_{P_L^c})_x\big )a\big ( (\check{\chi }_{P_L})_y\big )\Big ]\\&\quad \ge 0. \end{aligned}\end{aligned}$$

Here, we used that $\Lambda _+^* = P_L \cup P_L^c$ and we denote by $\check{\chi }_{S}$ the distribution which has Fourier transform $\chi _S$, the characteristic function of the set $S\subset \Lambda _+^*$.

Combining (7.3), (7.3), (7.3) and (7.84), it follows that

7.85 $$\begin{aligned} {\mathcal {M}}_N&\ge 4\pi {\mathfrak {a}}_0N^{1+\kappa }-4\pi {\mathfrak {a}}_0N^{\kappa -1}{\mathcal {N}}_+^{\,2} + 8\pi {\mathfrak {a}}_0N^\kappa \sum _{p\in P_H^c}\Big [ b^*_pb_p +\frac{1}{2} b^*_pb^*_{-p} + \frac{1}{2} b_pb_{-p}\Big ]\nonumber \\&\quad + \frac{8\pi {\mathfrak {a}}_0N^\kappa }{\sqrt{N}}\sum _{\begin{array}{c} p\in P_H^c,q\in P_L:\\ p+q\ne 0 \end{array}} \big [ b^*_{-p}a^*_{p+q}a_q +\text {h.c.}\big ]+\frac{1}{2}{\mathcal {K}}\nonumber \\&\quad +\frac{1}{2N}\sum _{\begin{array}{c} r\in P_H^c\cup \{0\}, v, w\in P_L: \\ (v,w)\in P_L^2\text { or }(v+r, w-r)\in P_L^2 \end{array}}^* N^\kappa \big ({\widehat{V}}(./N^{1-\kappa })*{\widehat{f}}_N\big )(r) a^*_{v+r}a^*_{w-r}a_v a_w\nonumber \\&\quad - {\widetilde{C}} N^{-\beta -\kappa }{\mathcal {V}}_{N,L} +{\mathcal {E}}'_{{\mathcal {M}}_N} \end{aligned}$$

Using Lemma 3.1, part ii), we have $\big ({\widehat{V}}(./N^{1-\kappa })*{\widehat{f}}_N\big )(0) = 8\pi {\mathfrak {a}}_0 + {\mathcal {O}}(N^{\kappa -1})$. This implies

7.86 $$\begin{aligned} \begin{aligned} {\mathcal {M}}_N&\ge 4\pi {\mathfrak {a}}_0N^{1+\kappa } + 8\pi {\mathfrak {a}}_0N^\kappa \sum _{p\in P_H^c}\Big [ b^*_pb_p +\frac{1}{2} b^*_pb^*_{-p} + \frac{1}{2} b_pb_{-p}\Big ]\\&\quad + \frac{8\pi {\mathfrak {a}}_0N^\kappa }{\sqrt{N}}\sum _{\begin{array}{c} p\in P_H^c,q\in P_L:\\ p+q\ne 0 \end{array}} \big [ b^*_{-p}a^*_{p+q}a_q +\text {h.c.}\big ]+\frac{1}{2}{\mathcal {K}}\\&\quad +\frac{1}{2N}\sum _{\begin{array}{c} r\in P_H^c , v, w\in P_L: \\ (v,w)\in P_L^2\text { or }(v+r, w-r)\in P_L^2 \end{array}}^* N^\kappa \big ({\widehat{V}}(. /N^{1-\kappa })*{\widehat{f}}_N\big )(r) a^*_{v+r}a^*_{w-r}a_v a_w\\&\quad - {\widetilde{C}} N^{-\beta -\kappa } {\mathcal {V}}_{N,L} + {\mathcal {E}}''_{{\mathcal {M}}_N}, \end{aligned} \end{aligned}$$

where, by (7.81) and Lemmas 4.2 and 7.1,

7.87 $$\begin{aligned} \begin{aligned} e^{A}e^D {\mathcal {E}}''_{{\mathcal {M}}_N} e^{-D}e^{-A}&\ge - C N^{-\beta } {\mathcal {K}}- C N^{-\beta -\kappa }{\mathcal {V}}_N -C N^{ \alpha +\beta +2\kappa -1}{\mathcal {K}}{\mathcal {N}}_{\ge \frac{1}{2} N^{\lfloor m_0 \rfloor \beta }} \\&\quad - C \sum _{j=3}^{2\lfloor m_0\rfloor -1}N^{ j\beta /2 + \beta /2+2\kappa -1}{\mathcal {K}}{\mathcal {N}}_{\ge \frac{1}{2} N^{j\beta /2}}- CN^{\alpha + \beta /2 +2\kappa } \end{aligned} \end{aligned}$$

Similarly, for $r\in P_H^c$, we know that

$$\begin{aligned} \big | \big ({\widehat{V}}(./N^{1-\kappa })*{\widehat{f}}_N\big )(r) - 8\pi {\mathfrak {a}}_0 \big | \le CN^{\alpha +\kappa -1}. \end{aligned}$$

Therefore, proceeding exactly as between (7.27) and (7.30), with $\big ({\widehat{V}}(./N^{1-\kappa })*{\widehat{f}}_N\big )(r)$ replaced by $\big | \big ({\widehat{V}}(/N^{1-\kappa })*{\widehat{f}}_N\big )(r) - 8\pi {\mathfrak {a}}_0 \big |$, we deduce that

7.88 $$\begin{aligned} {\mathcal {M}}_N&\ge 4\pi {\mathfrak {a}}_0N^{1+\kappa } +\frac{1}{2}{\mathcal {K}}+ 8\pi {\mathfrak {a}}_0N^\kappa \sum _{p\in P_H^c}\Big [ b^*_pb_p +\frac{1}{2} b^*_pb^*_{-p} + \frac{1}{2} b_pb_{-p}\Big ]\nonumber \\&\quad + \frac{8\pi {\mathfrak {a}}_0N^\kappa }{\sqrt{N}}\sum _{\begin{array}{c} p\in P_H^c,q\in P_L:\nonumber \\ p+q\ne 0 \end{array}} \big [ b^*_{-p}a^*_{p+q}a_q +\text {h.c.}\big ]\nonumber \\&\quad +\frac{4\pi {\mathfrak {a}}_0N^\kappa }{N}\sum _{\begin{array}{c} r\in P_H^c , v, w\in P_L: \\ (v,w)\in P_L^2\\ \text { or }(v+r, w-r)\in P_L^2 \end{array}}^* a^*_{v+r}a^*_{w-r}a_v a_w - {\widetilde{C}} N^{-\beta -\kappa } {\mathcal {V}}_{N,L} +{\mathcal {E}}'''_{{\mathcal {M}}_N}, \end{aligned}$$

with ${\mathcal {E}}'''_{{\mathcal {M}}_N}$ satisfying the same bound (7.87) as ${\mathcal {E}}''_{{\mathcal {M}}_N}$. Here we used Lemmas 4.2, 4.3, 7.1 and 7.2, as well as the assumption (5.6).

Finally, recalling the definition (5.1) and the identity (5.2), we find

7.89 $$\begin{aligned} \begin{aligned} {\mathcal {M}}_N&\ge 4\pi {\mathfrak {a}}_0N^{1+\kappa } +\frac{1}{2}{\mathcal {K}}+ 8\pi {\mathfrak {a}}_0N^\kappa \sum _{p\in P_H^c}\Big [ b^*_pb_p +\frac{1}{2} b^*_pb^*_{-p} + \frac{1}{2} b_pb_{-p}\Big ]\\&\quad + 8\pi {\mathfrak {a}}_0N^\kappa \sum _{p\in P_H^c } \Big [ b^*_{-p}e_{-p} + e^*_{-p}b_{-p} + b^*_{-p}e^*_{p} + e_{p}b_{-p} + b^*_{-p}c^*_{p} + c_p b_{-p} \Big ]\\&\quad +\frac{4\pi {\mathfrak {a}}_0N^\kappa }{N}\sum _{\begin{array}{c} r\in P_H^c , v, w\in P_L: \\ (v,w)\in P_L^2\\ \text { or }(v+r, w-r)\in P_L^2 \end{array}}^* a^*_{v+r}a^*_{w-r}a_v a_w - {\widetilde{C}} N^{-\beta -\kappa } {\mathcal {V}}_{N,L} + {\mathcal {E}}'''_{{\mathcal {M}}_N}\, . \end{aligned} \end{aligned}$$

To express also the first term in the third line of (7.89) in terms of the modified creation and annihilation fields defined in (5.1), we first observe that

$$\begin{aligned} \begin{aligned}&\frac{4\pi {\mathfrak {a}}_0N^\kappa }{N}\sum _{\begin{array}{c} r\in P_H^c , v, w\in P_L: \\ (v,w)\in P_L^2\\ \text { or }(v+r, w-r)\in P_L^2 \end{array}}^* a^*_{v+r}a^*_{w-r}a_v a_w\\&\quad = \frac{4\pi {\mathfrak {a}}_0N^\kappa }{N}\sum _{r\in P_H^c}\sum _{\begin{array}{c} v, w\in P_L: \\ (v,w)\in P_L^2\\ \text { or }(v+r, w-r)\in P_L^2 \end{array}}^* a^*_{v+r}a_v a^*_{w-r}a_{w} - \frac{4\pi {\mathfrak {a}}_0N^\kappa }{N}\sum _{\begin{array}{c} r\in P_H^c , v\in P_L: \\ (v,v+r)\in P_L^2 \end{array}}^* a^*_{v+r} a_{v+r}\\&\quad \ge \frac{4\pi {\mathfrak {a}}_0N^\kappa }{N}\sum _{r\in P_H^c}\sum _{\begin{array}{c} v, w\in P_L: \\ (v,w)\in P_L^2\\ \text { or }(v+r, w-r)\in P_L^2 \end{array}}^* a^*_{v+r}a_v a^*_{w-r}a_{w}- CN^{3\beta +\kappa -1}{\mathcal {N}}_+-C. \end{aligned}\end{aligned}$$

Then, for a fixed $r\in P_H^c$, we have that

$$\begin{aligned} \big \{ (v,w)\in \Lambda _+^*\times \Lambda _+^*:(v,w)\in P_L^2\text { or }(v+r,w-r)\in P_L^2 \big \}=\bigcup _{j=1}^7S_j, \end{aligned}$$

where

$$\begin{aligned} \begin{aligned} S_1&= \big \{ (v,w)\in \Lambda _+^*\times \Lambda _+^*: v\in P_L, w\in P_L, v+r\in P_L, w-r\in P_L \big \},\\ S_2&=\big \{ (v,w)\in \Lambda _+^*\times \Lambda _+^*: v\in P_L, w\in P_L, v+r\in P_L, w-r\in P_L^c \big \},\\ S_3&= \big \{ (v,w)\in \Lambda _+^*\times \Lambda _+^*: v\in P_L, w\in P_L, v+r\in P_L^c, w-r\in P_L \big \},\\ S_4&= \big \{ (v,w)\in \Lambda _+^*\times \Lambda _+^*: v\in P_L, w\in P_L, v+r\in P_L^c, w-r\in P_L^c \big \},\\ S_5&= \big \{ (v,w)\in \Lambda _+^*\times \Lambda _+^*: v\in P_L^c, w\in P_L, v+r\in P_L, w-r\in P_L \big \},\\ S_6&= \big \{ (v,w)\in \Lambda _+^*\times \Lambda _+^*: v\in P_L, w\in P_L^c, v+r\in P_L, w-r\in P_L \big \},\\ S_7&= \big \{ (v,w)\in \Lambda _+^*\times \Lambda _+^*: v\in P_L^c, w\in P_L^c, v+r\in P_L, w-r\in P_L \big \}. \end{aligned}\end{aligned}$$

In particular, the union $\bigcup _{j=1}^7S_j$ is a disjoint union. As a consequence, we find that

$$\begin{aligned} \begin{aligned}&\frac{4\pi {\mathfrak {a}}_0N^\kappa }{N}\sum _{r\in P_H^c}\sum _{\begin{array}{c} v, w\in P_L: \\ (v,w)\in P_L^2\\ \text { or }(v+r, w-r)\in P_L^2 \end{array}}^* a^*_{v+r}a_v a^*_{w-r}a_{w}\\&\quad = 8\pi {\mathfrak {a}}_0 N^\kappa \sum _{r\in P_H^c} \Big [ e^*_r c^*_{-r} + c_{-r}e_r+ \frac{1}{2}d^*_{r}e^*_{-r}+ \frac{1}{2}e_{-r}e_r +\frac{1}{2} c^*_{r}c^*_{-r} + \frac{1}{2} c_{-r}c_r \Big ] \\&\qquad + 8\pi {\mathfrak {a}}_0 N^\kappa \sum _{r\in P_H^c} \Big [ e^*_r e_r +c^*_r e_r + e^*_r c_r\Big ]. \end{aligned}\end{aligned}$$

Inserting in (7.88), we obtain

7.90 $$\begin{aligned} \begin{aligned} {\mathcal {M}}_N&\ge 4\pi \mathfrak {a}_0 N^{1+\kappa }+ \frac{1}{2} {\mathcal {K}}+ 8\pi {\mathfrak {a}}_0N^\kappa \sum _{r\in P_H^c} \big ( b^*_r + c^*_r +e^*_r \big )\big ( b_r + c_r +e_r \big )\\&\quad +4 \pi {\mathfrak {a}}_0N^\kappa \sum _{r\in P_H^c}\Big [ \big ( b^*_r + c^*_r +e^*_r \big )\big ( b^*_{-r} + c^*_{-r} +e^*_{-r} \big ) + \text {h.c.}\Big ] \\&\quad - 8\pi \mathfrak {a}_0 N^\kappa \sum _{r \in P_H^c} \left[ c_r^* c_r + b_r^* c_r + c_r^* b_r \right] - {\widetilde{C}} N^{-\beta - \kappa } {\mathcal {V}}_{N,L} + {\mathcal {E}}'''_{{\mathcal {M}}_N} \end{aligned} \end{aligned}$$

with

$$\begin{aligned}\begin{aligned} e^{A}e^D {\mathcal {E}}'''_{{\mathcal {M}}_N} e^{-D}e^{-A}&\ge - C N^{-\beta } {\mathcal {K}}- C N^{-\beta -\kappa }{\mathcal {V}}_N -C N^{ \alpha +\beta +2\kappa -1}{\mathcal {K}}{\mathcal {N}}_{\ge \frac{1}{2} N^{\lfloor m_0 \rfloor \beta }} \\&\quad - C \sum _{j=3}^{2\lfloor m_0\rfloor -1}N^{ j\beta /2 + \beta /2+2\kappa -1}{\mathcal {K}}{\mathcal {N}}_{\ge \frac{1}{2} N^{j\beta /2}}- CN^{\alpha + \beta /2 +2\kappa } \end{aligned} \end{aligned}$$

Let us now estimate the remaining terms on the last line of (7.90). For $\xi \in {\mathcal {F}}_+^{\le N}$, we have

7.91 $$\begin{aligned} \begin{aligned}&\bigg | \,8\pi {\mathfrak {a}}_0N^\kappa \sum _{r\in P_H^c} \langle \xi , c^*_r c_r \xi \rangle \bigg | \\&\quad \le \frac{CN^\kappa }{N} \!\!\!\!\sum ^*_{\begin{array}{c} r\in P_H^c, v,w\in P_L:\\ v\in P_L, r+v\in P_L^c,\\ w\in P_L, w+r\in P_L^c \end{array}} \Big ( |w||v|^{-1} \Vert a_{r+v}a _{w} \xi \Vert \Big ) \Big (|v||w|^{-1}\Vert a_va_{w+r} \xi \Vert \Big ) \\&\quad \le CN^{\beta + \kappa -1} \langle \xi , {\mathcal {K}}_L({\mathcal {N}}_{\ge N^\beta }+1) \xi \rangle , \end{aligned} \end{aligned}$$

and

7.92 $$\begin{aligned} \begin{aligned} \bigg | \,8\pi {\mathfrak {a}}_0N^\kappa \sum _{r\in P_H^c} \langle \xi , (b^*_r c_r + c^*_r b_r)\xi \rangle \bigg |&\le \frac{1}{4} \sum _{r\in P_H^c} \langle \xi , b^*_r b_r \xi \rangle +CN^{2\kappa } \sum _{r\in P_H^c} \langle \xi , c^*_r c_r \xi \rangle \\&\le \frac{1}{4} {\mathcal {K}}+ C N^{\beta + 2\kappa -1} \langle \xi , {\mathcal {K}}_L({\mathcal {N}}_{\ge N^\beta }+1) \xi \rangle , \end{aligned}\end{aligned}$$

Similarly, we can bound

$$\begin{aligned} \begin{aligned} N^{-\beta - \kappa } \langle \xi , {\mathcal {V}}_{N,L} \xi \rangle \le \;&C N^{-\beta -1} \sum _{\begin{array}{c} u\in \Lambda ^*, p,q \in \Lambda _+^*: \\ p+u, q+u, p,q \in P_L \end{array}} \Vert a_{p+u} a_{q} \xi \Vert \Vert a_p a_{q+u} \xi \Vert \\ \le \;&C N^{-\beta -1} \sum _{\begin{array}{c} u\in \Lambda ^*, p,q \in \Lambda _+^*: \\ p+u, q+u, p,q \in P_L \end{array}} \frac{|q|^2}{|p|^2} \Vert a_{p+u} a_{q} \xi \Vert ^2 \\ \le \;&C N^{-1} \Vert {\mathcal {K}}^{1/2} {\mathcal {N}}_+^{1/2} \xi \Vert ^2 \le C \Vert {\mathcal {K}}^{1/2} \xi \Vert ^2 \end{aligned} \end{aligned}$$

Thus, choosing the constant ${\widetilde{C}} > 0$ small enough and applying Lemmas 7.2, 4.3 and 4.2 to the r.h.s. of (7.91) and to the second term on the r.h.s. of (7.92), we conclude that

7.93 $$\begin{aligned} \begin{aligned} {\mathcal {M}}_N&\ge 4\pi \mathfrak {a}_0 N^{1+\kappa }+ \frac{1}{4} {\mathcal {K}}+ 8\pi {\mathfrak {a}}_0N^\kappa \sum _{r\in P_H^c} \big ( b^*_r + c^*_r +e^*_r \big )\big ( b_r + c_r +e_r \big )\\&\quad +4 \pi {\mathfrak {a}}_0N^\kappa \sum _{r\in P_H^c}\Big [ \big ( b^*_r + c^*_r +e^*_r \big )\big ( b^*_{-r} + c^*_{-r} +e^*_{-r} \big ) + \text {h.c.}\Big ] + {\mathcal {E}}''''_{{\mathcal {M}}_N} \end{aligned} \end{aligned}$$

where ${\mathcal {E}}''''_{{\mathcal {M}}_N}$ is such that

7.94 $$\begin{aligned} \begin{aligned} e^Ae^D {\mathcal {E}}''''_{{\mathcal {M}}_N} e^{-A} e^{-D} \ge&- C N^{-\beta } {\mathcal {K}}- C N^{-\beta -\kappa }{\mathcal {V}}_N \\&- C N^{\beta + 2\kappa - 1} {\mathcal {K}}{\mathcal {N}}_{\ge N^\beta } - C N^{ \alpha +\beta +2\kappa -1}{\mathcal {K}}{\mathcal {N}}_{\ge \frac{1}{2} N^{\lfloor m_0 \rfloor \beta }} \\&- C \sum _{j=3}^{2\lfloor m_0\rfloor -1}N^{ j\beta /2 + \beta /2+2\kappa -1}{\mathcal {K}}{\mathcal {N}}_{\ge \frac{1}{2} N^{j\beta /2}} - CN^{\alpha + \beta /2 +2\kappa } \end{aligned} \end{aligned}$$

We introduce the operators

$$\begin{aligned} g^*_r = b^*_r + c^*_r + e^*_r , \qquad g_r = b_r+c_r+e_r.\end{aligned}$$

With the algebraic identity

$$\begin{aligned} \begin{aligned} \sum _{r\in P_H^c }\Big [ g^*_rg_r + \frac{1}{2} g^*_r g^*_{-r} +\frac{1}{2} g_{-r} g_{r}\Big ]&= \frac{1}{2} \sum _{r\in P_H^c }\big ( g^*_r +g_{-r}\big ) \big ( g_r +g^*_{-r}\big ) - \frac{1}{2} \sum _{r\in P_H^c } [g_{r} , g^*_{r}], \end{aligned} \end{aligned}$$

we conclude that

$$\begin{aligned}\begin{aligned} {\mathcal {M}}_{N}&\ge 4\pi \mathfrak {a}_0 N^{1+\kappa }+ \frac{1}{4} {\mathcal {K}}- 4\pi {\mathfrak {a}}_0N^\kappa \sum _{r\in P_H^c} [g_{r} , g^*_{r}] + {\mathcal {E}}''''_{{\mathcal {M}}_N} \end{aligned}\end{aligned}$$

Since

$$\begin{aligned}\begin{aligned} {[}b_{r} , c^*_{r}] = [b_r, e^*_r] = [c_r, b^*_r] = [e_r, b^*_r] = [c_r, e^*_r] = [e_r,c^*_r] = 0, \end{aligned}\end{aligned}$$

we obtain that

$$\begin{aligned}\begin{aligned} {[}g_r, g^*_r]&= \frac{N-{\mathcal {N}}_+}{N} - \frac{1}{N}a^*_ra_r + \frac{1}{N} \sum _{\begin{array}{c} v\in \Lambda _+^*: v\in P_L, \\ v+r\in P_L^c \end{array} }a^*_va_v- \frac{1}{N} \sum _{\begin{array}{c} v\in \Lambda _+^*: v\in P_L, \\ v+r\in P_L^c \end{array} }a^*_{v+r}a_{v+r}\\&\quad + \frac{1}{4N} \sum _{\begin{array}{c} v\in \Lambda _+^*: v\in P_L, \\ v+r\in P_L \end{array} }a^*_va_v - \frac{1}{4N} \sum _{\begin{array}{c} v\in \Lambda _+^*: v\in P_L, \\ v+r\in P_L \end{array} }a^*_{v+r}a_{v+r}. \end{aligned} \end{aligned}$$

A straightforward computation then shows that

$$\begin{aligned} -4\pi {\mathfrak {a}}_0N^\kappa \sum _{p\in P_H^c} [g_{r} , g^*_{r}]\ge - C N^{3\alpha +\kappa } (1-{\mathcal {N}}_+/N) - C N^{3\alpha +\kappa } {\mathcal {N}}_+/N\ge - C N^{3\alpha +\kappa }. \end{aligned}$$

Thus

$$\begin{aligned} {\mathcal {M}}_{N} \ge 4\pi \mathfrak {a}_0 N^{1+\kappa }+ \frac{1}{4} {\mathcal {K}}+ {\mathcal {E}}_{{\mathcal {M}}_N} \end{aligned}$$

where $${\mathcal {E}}_{{\mathcal {M}}_N}$$ satisfies

$$\begin{aligned} \begin{aligned} e^Ae^D {\mathcal {E}}_{{\mathcal {M}}_N} e^{-A} e^{-D} \ge&- C N^{-\beta } {\mathcal {K}}- C N^{-\beta -\kappa }{\mathcal {V}}_N \\&- C N^{\beta +2\kappa -1} {\mathcal {K}}{\mathcal {N}}_{\ge N^\beta } - C N^{ \alpha +\beta +2\kappa -1} {\mathcal {K}}{\mathcal {N}}_{\ge \frac{1}{2} N^{\lfloor m_0 \rfloor \beta }} \\&- C \sum _{j=3}^{2\lfloor m_0\rfloor -1}N^{ j\beta /2 + \beta /2+2\kappa -1}{\mathcal {K}}{\mathcal {N}}_{\ge \frac{1}{2} N^{j\beta /2}}- CN^{3\alpha + \kappa } \end{aligned}\end{aligned}$$

This concludes the proof of Proposition 5.1. $\square$

## Footnotes

1 Going through the proof of [18, Theorem 5.1], one can observe that the authors actually show that $1 - \langle \varphi _0 , \gamma _N \varphi _0 \rangle \le C N^{-2/17}$.

2 For $\kappa > 0$, the rate (1.6) is not expected to be optimal. Bogoliubov theory predicts that the number of excitations of the Bose–Einstein condensate in a Bose gas with density $\rho$ is of the order $N \rho ^{1/2}$; see [5]. In our regime, this corresponds to $N^{3\kappa /2}$ excitations.

3 Observe that the renormalized potential with Fourier transform $8\pi \mathfrak {a}_0N^{-1+\kappa } \mathbf{1 } (|p| < N^\alpha )$ that emerges in our rigorous analysis after a series of unitary transformations is reminiscent of the interaction that appears through an ad hoc substitution in the pseudo-potential method of [12, 13].
